# Heterocyclic chemistry meets wound care: novel *p*-methyl-*m*-trifluoromethyl-phenyl-substituted pyrrol-2-one derivatives with antimicrobial and antibiofilm activity

**DOI:** 10.1039/d6ra05763b

**Published:** 2026-08-07

**Authors:** Ioana C. Marinas, Alina Nicolescu, Cristina M. Al-Matarneh, Rafaela Vrabie, Sergiu Shova, Mihaela Silion, Mădălina-Diana Gaboreanu, Miruna Stan, Mariana C. Chifiriuc, Mariana Pinteala

**Affiliations:** a Research Institute of the University of Bucharest-ICUB 91-95 Spl. Independentei 050095 Bucharest Romania; b NMR Laboratory, “Petru Poni” Institute of Macromolecular Chemistry of Romanian Academy 41A Grigore Ghica Voda Alley Iasi 700487 Romania; c Centre of Advanced Research in Bionanoconjugates and Biopolymers, “Petru Poni” Institute of Macromolecular Chemistry of Romanian Academy 41A Grigore Ghica Voda Alley Iasi 700487 Romania almatarneh.cristina@icmpp.ro +40748683402; d “Cristofor Simionescu” Faculty of Chemical Engineering and Environmental Protection, “Gheorghe Asachi” Technical University of Iasi 73 D. Mangeron Blvd. 700050 Iasi Romania; e Department of Inorganic Polymers, “Petru Poni” Institute of Macromolecular Chemistry of Romanian Academy 41A Grigore Ghica Voda Alley Iasi 700487 Romania; f Physics of Polymers and Polymeric Materials Department, “Petru Poni” Institute of Macromolecular Chemistry 41A Grigore Ghica Voda Alley Iasi 700487 Romania; g Department of Botany and Microbiology, Faculty of Biology Splaiul Indeopenentei 91-95 Bucharest R-050095 Romania; h Faculty of Biology, Department of Biochemistry and Molecular Biology, University of Bucharest 91-95 Splaiul Independentei Bucharest 050095 Romania; i The Romanian Academy 25, Calea Victoriei, Sector 1, District 1 Bucharest 010071 Romania

## Abstract

Biofilm-associated infections represent a major challenge in the treatment of antimicrobial-resistant pathogens, particularly in chronic and wound-related infections where bacterial adherence and persistence reduce therapeutic efficacy. To address this issue, we report a rapid, economical, and efficient one-pot three-component synthesis of 19 novel bis(trifluoromethyl)-dimethyl-substituted dihydro-pyrrol-2-one derivatives, providing a promising scaffold for the development of new antimicrobial and anti-biofilm agents. The structures of the synthesized compounds were confirmed by NMR, FT-IR, MALDI-MS, and X-ray diffraction analyses. ADMET prediction revealed highly similar pharmacokinetic profiles across the series, supporting the suitability of this scaffold for further medicinal chemistry optimization. Biological evaluation demonstrated that antimicrobial activity was strongly influenced by the nature of the substituents. Compounds 4i, 4j, 4l, 4n, 4o, 4p, and 4t exhibited the highest activity against *Staphylococcus aureus*, while compounds 4o and 4s were particularly effective against *Pseudomonas aeruginosa*. Importantly, most derivatives significantly inhibited microbial adherence, highlighting their potential to prevent biofilm formation. Furthermore, all compounds exhibited low hemolytic activity (<5%), and more than 70.59% displayed acceptable cytocompatibility. Among the series, compounds 4i, 4l, and 4s showed the most favorable biological profile, combining potent antimicrobial activity with good compatibility toward HaCaT keratinocytes and blood cells. These findings identify bis(trifluoromethyl)-dimethyl-substituted dihydro-pyrrol-2-ones as promising candidates for the development of novel therapeutics targeting biofilm-associated and drug-resistant infections.

## Abstract 2

Biofilm-associated infections represent a major challenge in the treatment of antimicrobial-resistant pathogens, particularly in chronic and wound-related infections where bacterial adherence and persistence reduce therapeutic efficacy.

## Graphical abstract

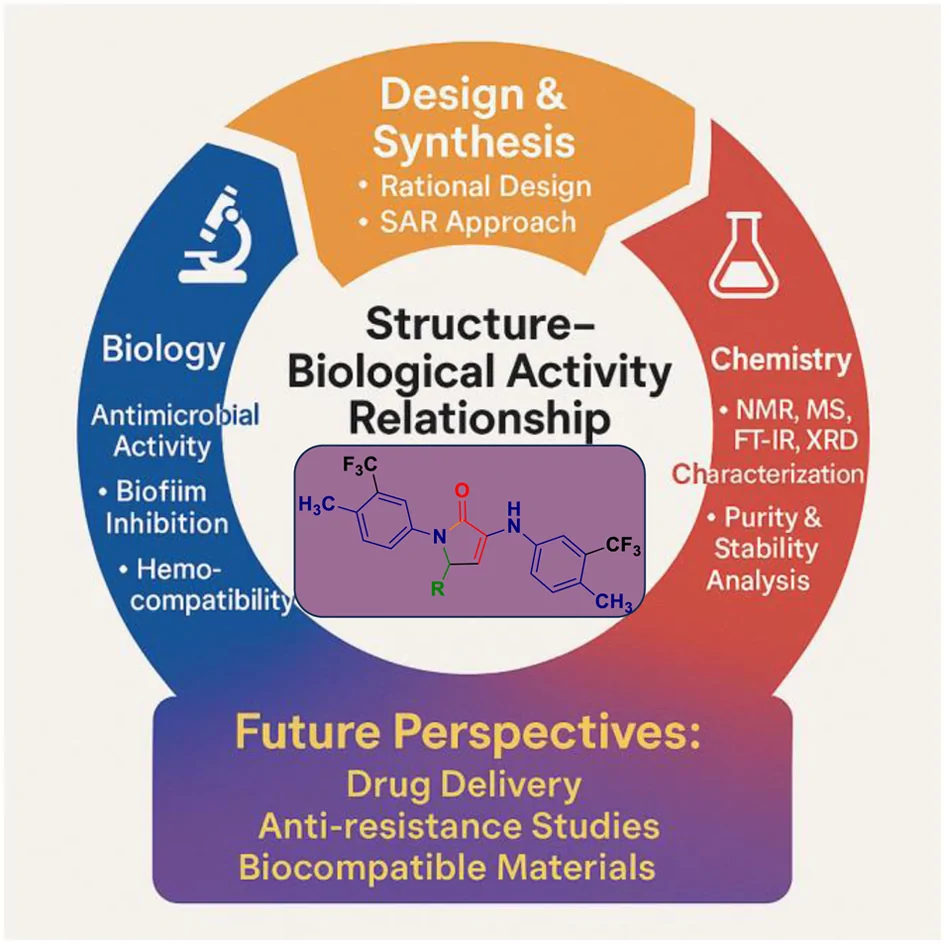

## Introduction

The increasing issue of antimicrobial resistance (AMR), principally driven by the widespread and often indiscriminate use of antimicrobial drugs in human healthcare, veterinary medicine, animal husbandry, and aquaculture is affecting nearly every field,^1,2^ rendering the fight against harmful germs a collective obligation of all nations.^3^ Public health, the economy, and healthcare systems all suffer when antibiotics lose effectiveness, raising an acute need for medical treatments that remain effective in the long run, consisting either of the development of new antimicrobial drugs or optimizing the existent treatments through synergisms, targeted delivery systems or inhibiting the resistance mechanisms.^4^

Pathogenic microorganisms have developed many resistance strategies, including altered permeability that limits intracellular drug accumulation, overexpression of efflux transporters that expel xenobiotics from the cell, target modification/inaccessibility, antimicrobial agents enzymatic inactivation/modification,^3^ bypassing certain metabolic pathways or shifting from planktonic to biofilm growth.^5^ Biofilms represent one of the so-called phenotypic resistance strategies, in which microbial communities adhere to an interface and secrete an extracellular polymeric substance matrix (EPS).^6^ The biofilm microbial cells are protected, becoming highly resistant to different types of stressors, including anti-infective immune defense mechanisms, antimicrobials and antiseptics. Biofilm-associated resistance is particularly important for chronic infections, such as chronic wounds, which are a large and growing problem globally, affecting millions of individuals and imposing substantial costs in healthcare and other sectors.^7^ More than half of chronic wounds are frequently associated with biofilm-related infections, this being a major factor contributing to treatment failure, impaired healing process and persistence over months or even years. Chronic wounds might be colonized and infected with different resistant microorganisms, including two common opportunistic pathogens, *Staphylococcus aureus*^8^ and *Pseudomonas aeruginosa*,^9^ which are on the WHO priority lists for the development of novel therapeutic strategies. These bacteria are among the most potent biofilm producers and possess a large spectrum of virulence factors that impede tissue repair and perpetuate inflammation. Among fungal species, *Candida albicans*^10^ is often involved in polymicrobial biofilms and acts synergistically to reduce treatment efficacy.^11^

The search for new antimicrobial agents has increasingly shifted toward molecules exhibiting alternative mechanisms of action and enhanced activity against resistant microorganisms. Recent studies emphasize that structurally diverse compounds capable of interfering with microbial survival and persistence represent promising candidates for combating antimicrobial resistance.^3,4,12^ Such approaches are particularly relevant for biofilm-associated infections, where conventional antimicrobial therapies frequently fail and innovative anti-biofilm strategies are urgently needed.^10^

Taking into account the important role of biofilms in protecting pathogens for the immune system and drugs, new treatments for persistent wound infections should target breaking up these sessile structures. In this respect, small heterocyclic compounds have attracted considerable interest due to their antimicrobial and antibiofilm characteristics.

Nitrogen-containing heterocycles are highly regarded as superior scaffolds in medicinal chemistry owing to their capacity to engage with diverse biological targets and augment pharmacological activity.^3,4,6,7^ Among them, substituted dihydro-2*H*-pyrrol-2-ones have pronounced antimicrobial characteristics, with the five-membered heterocyclic core facilitating precise modifications to electronic, steric, and lipophilic characteristics, which are necessary for biofilm penetration and killing microbes.^13^

Parallel with heterocycle development, fluorine incorporation has become a strategic tool in medicinal chemistry for enhancing drug-like properties. The small size, high electronegativity, and unique electronic effects of fluorine can dramatically influence target binding affinity, metabolic stability, membrane permeability, and overall pharmacokinetics of lead compounds. As a result, fluorinated small molecules, encompassing antibacterial agents like fluoroquinolones and other fluorine-containing heterocycles, feature prominently in modern antimicrobial drug discovery and clinical use, due to their enhanced efficacy against pathogens and anti-biofilm activity.^14^

Fluorine chemistry continues to develop novel synthetic methodologies for the production of antimicrobial pharmaceuticals, including those highly performant in eliminating biofilms associated to chronic wounds.^15^

Building on our previous studies in Doebner synthesis^16–19^ and *N*-heterocycle construction,^20,21^ we present the synthesis and biological evaluation of a new series of *p*-methyl-*m*-trifluoromethyl pyrrol-2-one compounds. Through the integration of synthetic chemistry and biological profiling, this study seeks to develop biocompatible heterocyclic compounds with antimicrobial activity and the ability to interfere with early-stage microbial adhesion, as preliminary candidates for further investigation in the context of biofilm-associated wound infections.

## Results and discussions

### Chemistry

The trifluoromethyl group is a privileged structural motif in medicinal chemistry due to its ability to modulate the electronic, lipophilic, and metabolic properties of drug-like molecules. As a result, fluorinated compounds are extensively explored in pharmaceutical research and are represented in numerous clinically used therapeutics.^22^ Encouraged by our previous report on trifluoromethyl-substituted pyrrol-2-one derivatives displaying promising antimicrobial activity,^17^ we designed and synthesized a new series of analogues bearing both trifluoromethyl and methyl substituents. The introduction of the methyl moiety was aimed at assessing its impact on the chemical and biological profiles of the scaffold, as well as providing additional structure–activity relationship (SAR) information for this class of pyrrol-2-one derivatives.

**Scheme 1:**
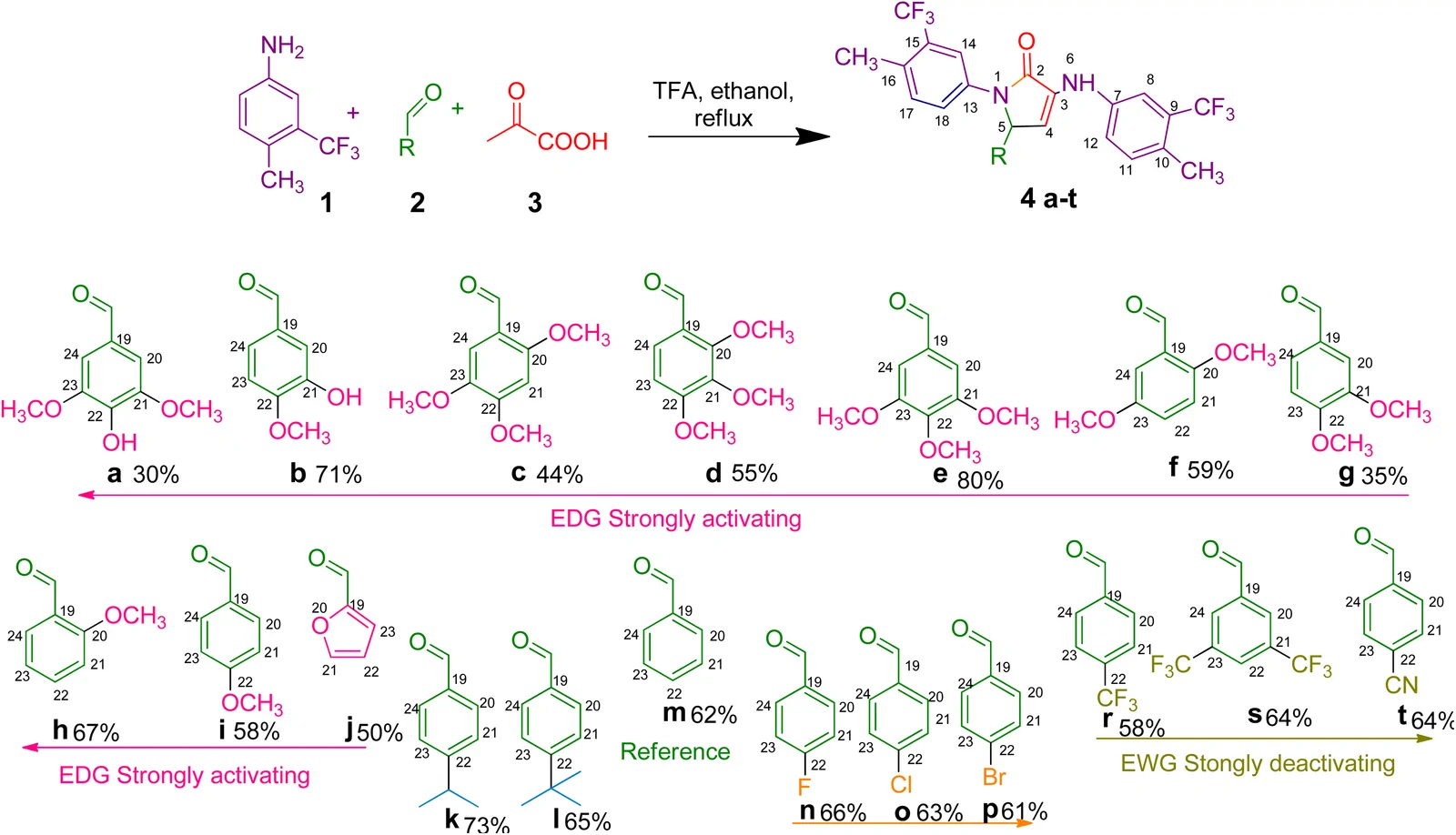
Reaction pathway for compounds 4a–t.

Briefly, a series of novel pyrrol-2-one derivatives was synthesized through a one-pot, three-component reaction involving 4-methyl-3-(trifluoromethyl)aniline, appropriately selected aldehydes, and pyruvic acid in ethanol, using trifluoroacetic acid (TFA) as catalyst. The methodology provided efficient access to structurally diverse fluorinated pyrrol-2-one scaffolds under mild reaction conditions. Given the increasing emphasis in medicinal chemistry on step-economical, operationally simple, and sustainable synthetic approaches, the present protocol represents a practical strategy for the rapid generation of compound libraries suitable for biological evaluation (Scheme 1).

All compounds were obtained as racemic mixtures in good isolated yields (30–80%) and were used as such in all subsequent analyses, computational studies, and biological investigations. Their structures were unambiguously confirmed by one-dimensional (^1^H and ^13^C) and two-dimensional NMR spectroscopy, together with FT-IR, MALDI-MS analyses and for compounds 4e, 4g, 4n, 4o, 4p, 4r, 4s and 4t confirmed by single crystal X-ray diffraction.

While the previously reported series afforded isolated yields ranging from 32 to 92%,^17^ the current fluorinated scaffold was obtained in moderate to good isolated yields (30–80%) across a diverse range of substituents, demonstrating the broad applicability of the synthetic methodology. Notably, electron-withdrawing groups that negatively impacted the yields in the earlier study were better tolerated in the present system, suggesting that the reaction pathway leading to the bis(trifluoromethyl)-substituted dihydro-pyrrol-2-ones is less sensitive to electronic perturbations. This improved substrate tolerance represents an advantage for the rapid generation of compound libraries intended for biological screening.

One of the most significant synthetic and mechanistic observations emerging from the present study concerns the pronounced effect of aniline substitution on the reaction outcome. Encouraged by our previous results, we investigated the impact of introducing an additional methyl substituent into the trifluoromethylated aniline precursor. Interestingly, in contrast to the previously reported system bearing only a CF_3_ substituent,^17^ the use of methyl-trifluoromethyl-substituted aniline led exclusively to the formation of pyrrol-2-one derivatives, whereas no furan-type products or other by-products were detected under identical reaction conditions.

This finding indicates that even small structural modifications of the aniline component can substantially alter the reaction pathway and product distribution. The exclusive formation of pyrrol-2-one derivatives, accompanied by the absence of quinoline products, suggests that the electron-withdrawing influence of the trifluoromethyl substituent remains dominant despite the presence of the electron-donating methyl group. This observation indicates that the introduction of the methyl substituent is insufficient to alter the overall electronic character of the aniline precursor to an extent that would favor alternative cyclization pathways. At the same time, the complete suppression of furan formation points to a significant effect of methyl substitution on reaction selectivity. While the precise origin of this behavior remains to be elucidated, steric and electronic factors introduced by the methyl group may influence the stability and reactivity of key intermediates, ultimately directing the transformation preferentially toward the pyrrol-2-one scaffold and enhancing the chemoselectivity of the process. When considered alongside our previous investigations^16,17,23^ of related electron-withdrawing-substituted aniline systems, the present findings further emphasize the pivotal role of both the aniline and aldehyde components in determining the outcome of this multicomponent transformation. The cumulative experimental evidence indicates that relatively small variations in substrate electronic properties can markedly influence reaction selectivity, leading to distinct heterocyclic scaffolds under otherwise identical reaction conditions.

Although the detailed reaction mechanism remains to be fully elucidated, the results obtained across these studies provide valuable mechanistic insight into the factors governing product formation. In particular, they suggest that the electronic characteristics of electron-deficient anilines, together with the nature of the aldehyde partner, influence the stability and reactivity of key intermediates, ultimately directing the reaction toward different cyclization pathways. The exclusive formation of pyrrol-2-one derivatives observed in the present work further supports the notion that subtle changes in substrate structure can substantially affect chemoselectivity.

Collectively, these studies represent important incremental advances toward a more comprehensive understanding of the reaction mechanism and establish a foundation for the rational development of future synthetic methodologies based on selectively substituted aniline precursors.

The analytical data (both FT-IR and MALDI-MS) are in excellent agreement with the suggested structures, thereby confirming the integrity of the synthesized compounds and validating the proposed molecular frameworks.^17–19^ All the obtained compounds 4a–t were analyzed through NMR spectroscopy and their proton NMR spectra contained the two doublets with 3 Hz coupling constant, visible in the interval 6.00–6.40 ppm, previously assigned by us as “markers” for the formation of dihydro-pyrrol-2-one cycle.^7–10^ The fluorine atom from trifluoromethyl residue gives rise to additional signals splitting for carbons situated in its vicinity. These carbon-fluorine scalar couplings are identified in the ^13^C-NMR spectra after the quartet pattern with coupling constants ranging from 270 to 4 Hz and were described by us in a previous paper.^7^ The unambiguous proton and carbon signals assignments for all newly compounds, obtained from bidimensional correlation experiments, together with fluorine and nitrogen chemical shifts, are included in the Experimental section.

**Fig. 1:**
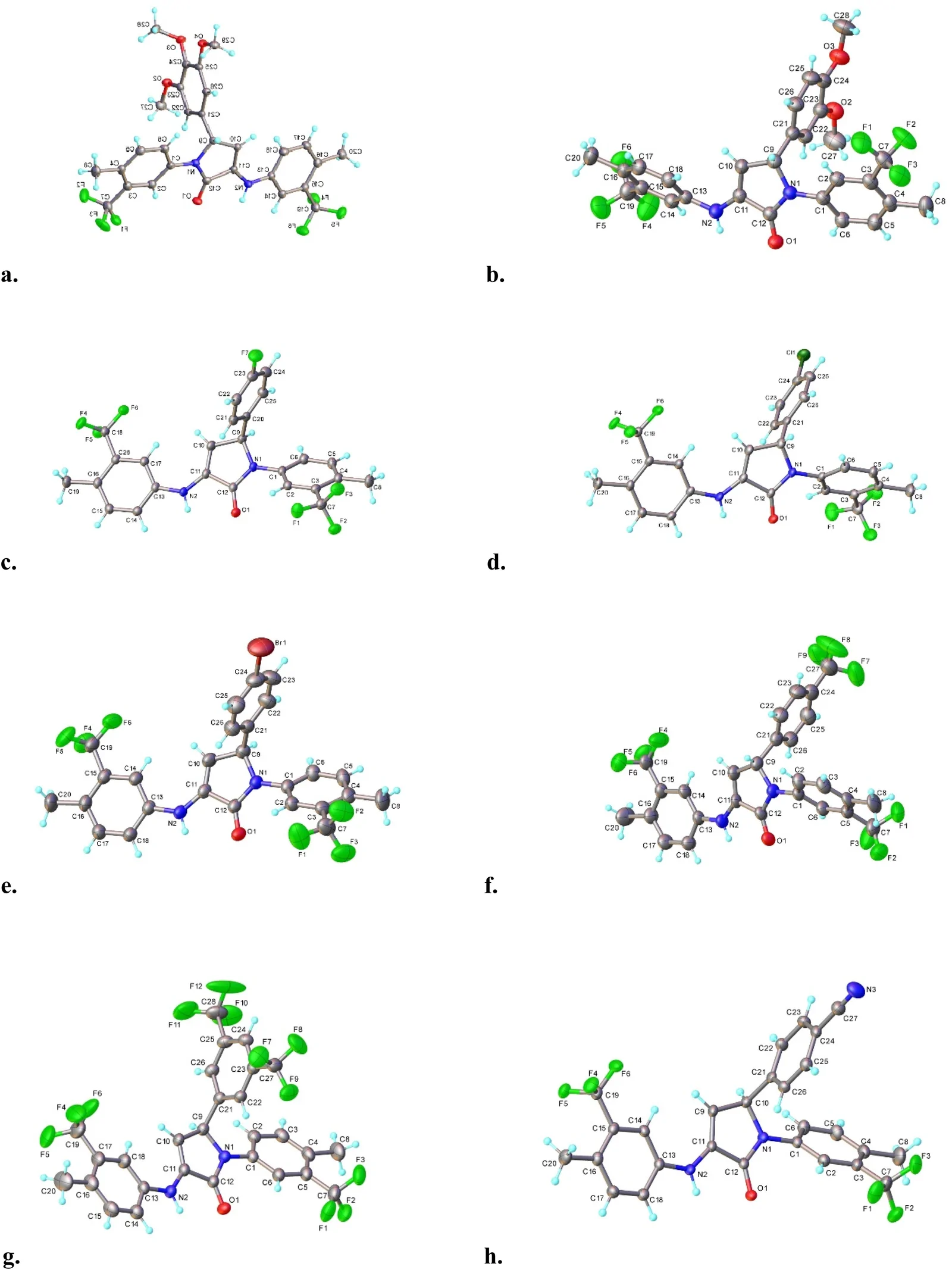
X-ray molecular structure with atom labeling for compounds 4e (a), 4g (b), 4n (c), 4o (d), 4p (e), 4r (f), 4s (g), 4t (h).

According to X-ray crystallography, all the compounds exhibit a molecular crystal structure with one neutral unit in the asymmetric part of the unit cell. The results of X-ray diffraction study for 4e, 4g, 4n, 4o, 4p, 4r, 4s and 4t are illustrated in Fig. 1a–h. The geometric parameters are summarized in Table 2.

The structure 4o was refined as a 2-component non-merohedral twin accounted by introducing the instruction TWIN 1̄0001̄0001. The refinement showed that the fractional contributions of the two twin domains are of 0.58 : 0.42. There were no co-crystallized solvent molecules in the crystals except compound contains two interstitial molecules of ethanol per asymmetric unit.

### *In silico* ADMET evaluation of compounds 4a–4t

**Fig. 2:**
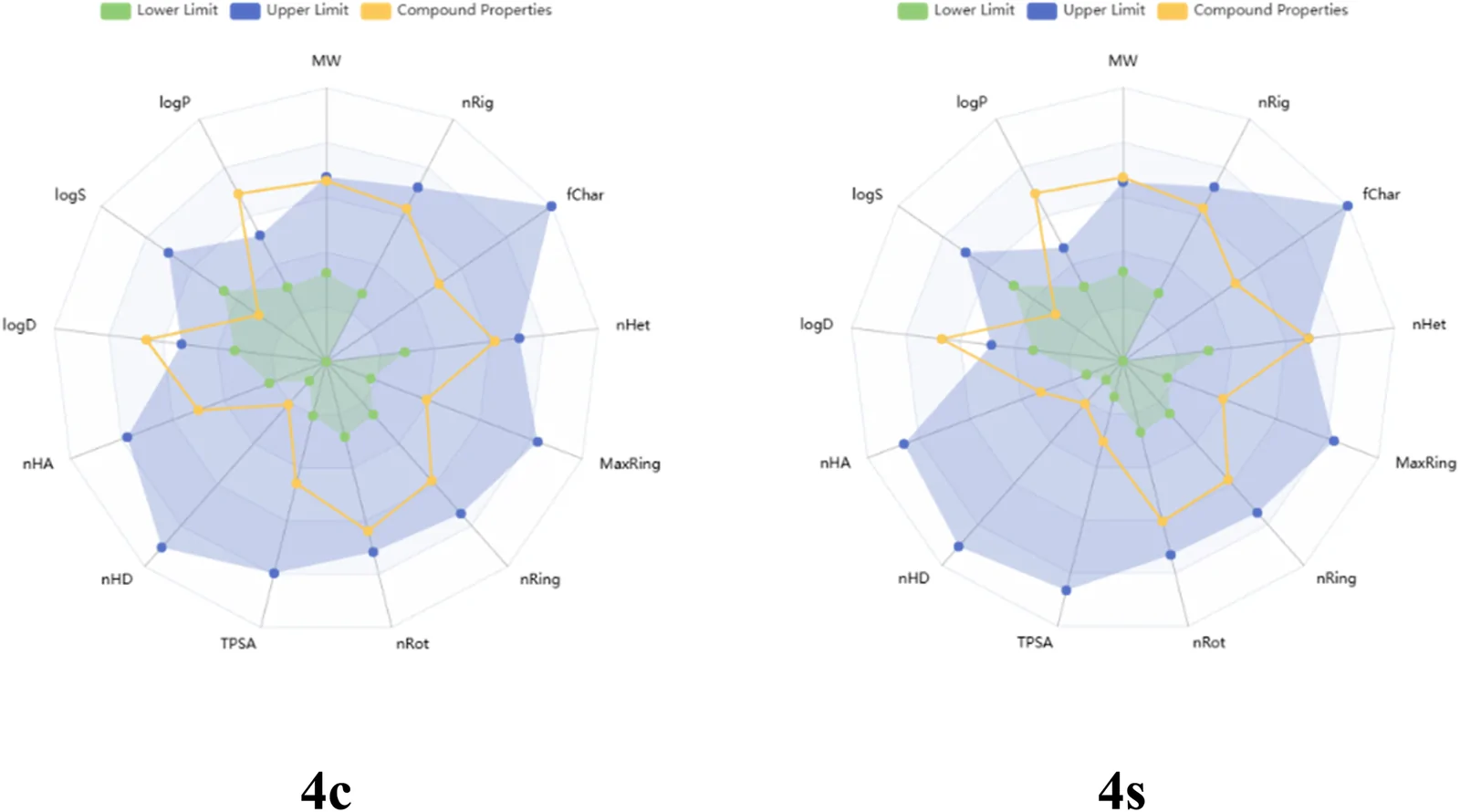
The radar generated from the ADMET web tool, regarding the accessibility of compounds 4c and 4s to be bioavailable.

*In silico* evaluation of compounds 4a–4t using the ADMET (Absorption, Distribution, Metabolism, Excretion and Toxicity) platform revealed an overall acceptable pharmacokinetic profile, with most parameters falling within the recommended ranges for drug-like molecules (Fig. 2).

However, the entire series exhibited log *P* and log *D* values above the optimal threshold, indicating increased lipophilicity, which is associated with good membrane permeability but may also lead to limitations in solubility and bioavailability. Consistent with this, log *S* values were below the recommended limit, suggesting poor aqueous solubility. The relatively high lipophilicity may contribute to membrane interactions and permeability properties relevant for antimicrobial activity. The predicted permeability across biological membranes is generally acceptable, supporting the potential for intracellular accumulation; however, the high plasma protein binding may limit the fraction of free, active compounds in systemic circulation. Additionally, the molecular weight of the compounds was at the upper limit of the acceptable range, which may influence distribution and permeability. An exception was compound 4s, which also displayed a nHET value at the upper limit, indicating slightly higher polarity compared to the rest of the series. Overall, ADMET platform testing provides a useful preliminary insight into the pharmacokinetic behavior of the investigated compounds and highlights aspects that may guide further structural optimization.

Further ADMET evaluation of compounds 4a–4t suggests that the entire series shares a broadly similar pharmacokinetic profile, with only small variations among individual derivatives. The compounds are predicted to undergo CYP-mediated metabolism, indicating that metabolic stability and potential drug–drug interactions should be considered in further development. Overall, while all members of the 4a–4t series behave more or less identically in the ADMET assessment, the small observed variations could be leveraged in future structural optimization to achieve a better balance between antimicrobial activity, pharmacokinetics, and safety (see SI file).

### Antimicrobial investigations

The study of antimicrobial activity against *S. aureus*, *P. aeruginosa*, and *C. albicans* is crucial for topical applications due to their frequent association with skin infections and wounds. *S. aureus* is a Gram-positive bacteria found in skin and soft tissue infections,^24^ while *P. aeruginosa* is a Gram-negative bacteria involved in burn wounds and chronic ulcers.^25^ Addressing these pathogens is essential for wound care, as they can cause delayed healing, persistent infection, and an increased risk of sepsis. *C. albicans* could also be involved in wound infections, especially in areas prone to humidity.^26^ Given the polymicrobial nature of wound infections, combined topical therapy is essential for their effective management.

The trifluoromethyl group (–CF_3_) is an essential functional group in medicinal chemistry as it enhances the bioactivity of molecules, including antibacterial effect and inhibiting bacterial adherence on inert surface.^27^ Its high electron attraction can influence molecular features such as affinity to bacterial targets, lipophilicity, metabolic stability, and membrane permeability, all of which being important determinants of the antimicrobial effect.^27^

The minimum inhibitory concentration (MIC) values were determined using the twofold microdilution method. The antimicrobial activity of the *p*-methyl-*m*-trifluoromethyl compounds was compared with the base structure and the solvent (as shown in Table 1). According to the data in Table 1, all the compounds demonstrated strong antimicrobial activity against *S. aureus*, moderate against *P. aeruginosa*, and weak against *C. albicans*, depending on the different substituents attached to the core structure. The order of the compounds efficacy against *S. aureus* was 4i = 4j = 4l = 4n = 4o = 4p = 4t < 4b = 4s < 4a = 4c = 4d < 4e = 4f = 4m = 4r = DMSO<4h = 4k, for *P. aeruginosa*4o < 4s < 4a = 4b = 4c = 4d = 4e = 4f = 4h = 4i = 4l = 4r = 4t = DMSO<4k = 4m = 4n < 4j < 4p, and for *C. albicans*4c = 4d = 4j = 4l = 4n = 4s = DMSO<4a = 4b = 4e = 4m = 4r = 4t < 4h = 4k < 4i = 4o = 4p. The compounds 4c, 4d, 4j, 4l, 4n, and 4s have shown a weak antimicrobial activity, the MIC being similar to that of the solvent. Some derivatives did not inhibit or, in certain cases, even promoted microbial growth, leading to higher values compared to those attributed solely to solvent control (DMSO). While in the case of *S. aureus*, nine of the obtained compounds exhibited both MIC and MMC inferior to those of the solvent, in case of the *P. aeruginosa*, only one compound (4o) and none in case of *C. albicans* showed this performance (Table 1).

**Table 1:** Antimicrobial activity parameters: MIC (mg mL^−1^), MMC (mg mL^−1^), and MICMA (mg mL^−1^). The MIC and MICMA values lower than the solvent (DMSO) were highlighted chromatically: yellow for MIC and blue for MICMA^a^

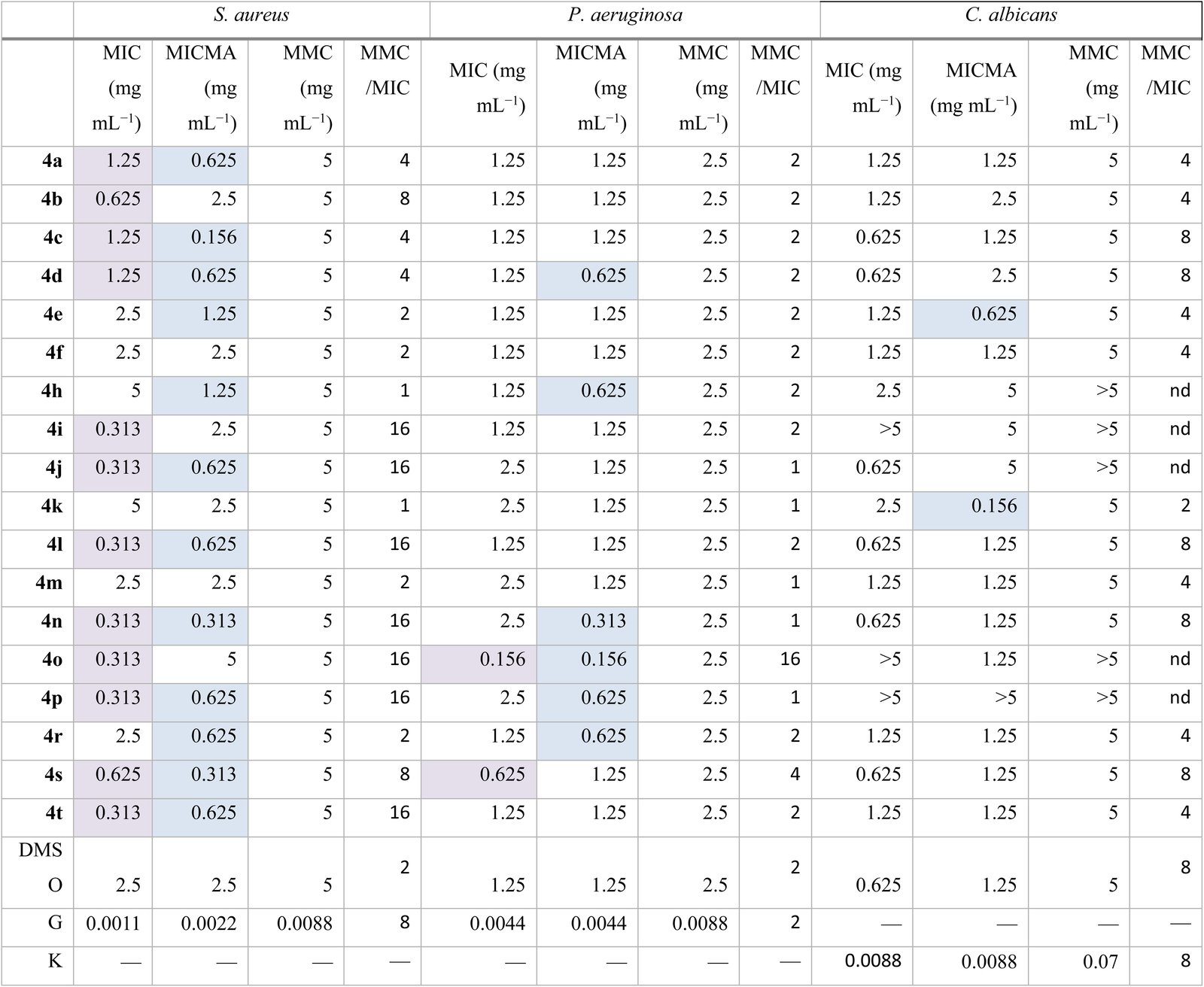

a MIC: minimum inhibitory concentration at which bacterial growth was 99% inhibited; MMC: minimum concentration that leads to the absence of growth on solid medium (the inability to form colonies) after subculturing from a liquid medium; MICMA: minimum inhibitory concentrations of microbial adherence; G: gentamicin; K: ketoconazol.

Crystal data and details of structure refinement for compounds 4e, 4g, 4n, 4o, 4p, 4r, 4s and 4t

**Table:** 

Compound	4e	4g	4n	4o
Emp. formula	C_33_H_38_F_6_N_2_O_6_	C_30.25_H_29.25_F_6_N_2.75_O_3.75_	C_26_H_19_F_7_N_2_O	C_26_H_19_ClF_6_N_2_O
*F*_w_	672.65	605.31	508.43	524.88
*T* [K]	100	297	100	100
Space group	*P*1̄	*P*1̄	*P*1̄	*P*1̄
*a* [Å]	10.7483(2)	7.9436(2)	6.5744(2)	6.46412(12)
*b* [Å]	12.1927(3)	13.0011(3)	12.2959(4)	12.4306(2)
*c* [Å]	13.3457(2)	15.6299(4)	13.9486(4)	14.5129(6)
*α* [°]	109.9769(18)	113.268(2)	78.086(3)	79.960(3)
*β* [°]	100.7301(17)	94.583(2)	86.079(3)	86.776(3)
*γ* [°]	97.6036(19)	97.137(2)	85.783(3)	84.8709(15)
*V* [Å^3^]	1578.21(6)	1456.60(7)	1098.67(6)	1142.71(6)
*Z*	2	2	2	2
*ρ*_calcd_ [g cm^−3^]	1.415	1.380	1.537	1.525
*µ* [mm^−1^]	1.033	1.008	1.181	2.140
Crystal size [mm]	0.25 × 0.10 × 0.04	0.10 × 0.05 × 0.05	0.12 × 0.05 × 0.03	0.35 × 0.10 × 0.03
2*θ* range	7.28 to 154.10	6.218 to 133.198	6.49 to 153.90	6.19 to 133.18
Refls. collected	19 699	17 403	12 501	3954
Indep. refls., *R*_int_	6318, 0.0325	5128, 0.0214	4346, 0.0446	3954
Data/rests./params.	6318/4/454	5128/0/356	4346/0/327	3954/0/328
GOF	1.068	1.057	1.054	1.079
*R*_1,_ *wR*_2_(all data)	0.0308, 0.1038	0.0430, 0.1309	0.0379, 0.0988	0.0497, 0.1347
CCDC no.	2372610	2372614	2372611	2372612

**Table:** 

Compound	4p	4r	4s	4t
Emp. formula	C_26_H_19_BrF_6_N_2_O	C_27_H_19_F_9_N_2_O	C_28_H_18_F_12_N_2_O	C_27_H_19_F_6_N_3_O
*F*_w_	569.34	558.44	626.44	515.45
*T* [K]	297	295	200	100
Space group	*P*1̄	*P*1̄	*P*2_1_/*n*	*P*1̄
*a* [Å]	6.82452(14)	6.8330(3)	15.67450(10)	6.4780(2)
*b* [Å]	12.5300(3)	12.5294(5)	7.99180(10)	12.3216(3)
*c* [Å]	15.0423(4)	15.3063(6)	21.3342(2)	14.6974(2)
*α* [°]	103.286(2)	103.324(3)	90	81.708(2)
*β* [°]	102.438(2)	102.349(4)	95.6500(10)	85.521(2)
*γ* [°]	93.6268(17)	93.983(3)	90	84.685(2)
*V* [Å^3^]	1213.85(5)	1235.88(9)	2659.50(5)	1153.38(5)
*Z*	2	2	4	2
*ρ*_calcd_ [g cm^−3^]	1.558	1.501	1.565	1.484
*µ* [mm^−1^]	2.931	1.225	1.377	1.084
Crystal size [mm]	0.20 × 0.10 × 0.03	0.30 × 0.15 × 0.02	0.25 × 0.10 × 0.03	0.20 × 0.10 × 0.02
2*θ* range	6.214 to 133.2	6.108 to 133.19	6.69 to 154.29	6.09 to 133.17
Refls. collected	13 152	11 949	18 033	12 745
Indep. refls., *R*_int_	4277, 0.0245	4247, 0.0794	5236, 0.0147	4064, 0.0207
Data/rests./params.	4277/0/327	4247/0/354	5236/18/390	4064/0/336
GOF	1.171	1.082	1.071	1.051
*R*_1,_ *wR*_2_(all data)	0.0523, 0.1654	0.0895, 0.2802	0.0593, 0.1730	0.0389, 0.0987
CCDC no.	2372613	2372615	2372608	2372609

**Fig. 3:**
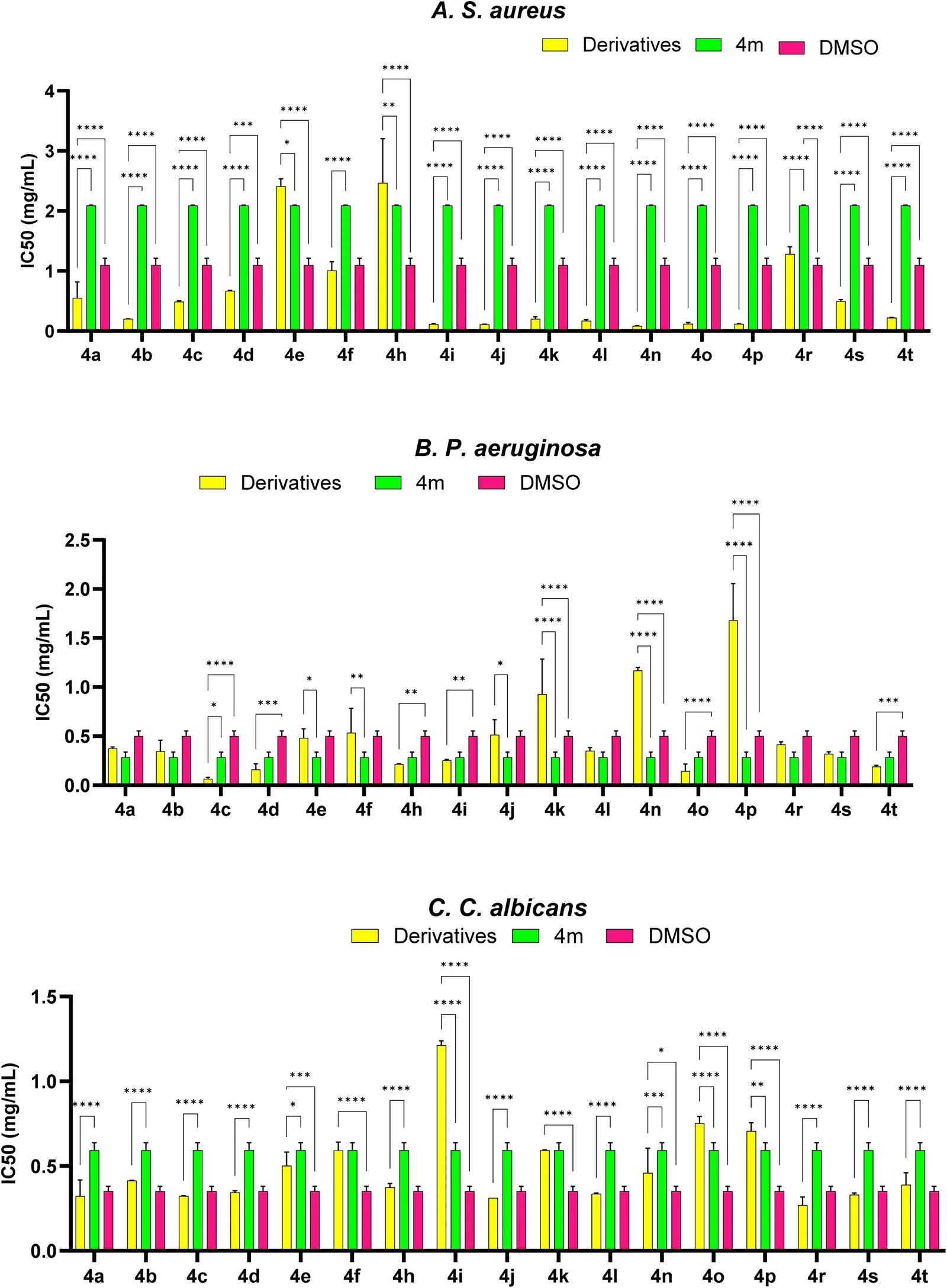
IC_50_ values (mg mL^−1^) for each synthesized derivative were determined in comparison with the parent scaffold (compound 4m) and the solvent control against *S. aureus* (A), *P. aeruginosa* (B) and *C. albicans* (C). Compound 4m represents the unsubstituted core structure of the series and was included as a reference for the preliminary structure–activity relationship analysis. Statistical significance is indicated as *p < 0.05, **p < 0.01, ***p < 0.001 and ****p < 0.0001.

To assess the structure–activity relationship, the IC_50_ values (the concentration at which 50% inhibition occurs) were calculated statistically for each compound and microbial strain, to quantify their effectiveness (Fig. 3).

The antimicrobial activity model based on IC_50_ against Gram-positive bacteria (*S. aureus*) can be observed in Fig. 3A. The best antimicrobial activity against the *S. aureus* strain was obtained for 4n (4-fluorophenyl), 4j (furan-2-yl), and 4i (4-methoxyphenyl). The influence of the 4-fluorophenyl group on the antimicrobial activity against Gram-positive bacteria may be related to physicochemical and electronic effects described for similar fluorinated aromatic derivatives in the literature.^28^ The core structure (4m) significantly increased the IC_50_ value compared to the solvent control (*p* < 0.0001). Halogens and methoxy radicals have been reported to influence lipophilicity and interactions with biological targets in related classes of compounds.^29^

The Gram-negative bacteria, including the tested *P. aeruginosa* strain, are generally more resistant than Gram-positive bacteria due to the presence of an additional barrier called the outer membrane. The weak activity against *P. aeruginosa* observed in the MIC and MMC assays was confirmed by the IC_50_ analysis (Fig. 3B) indicating that the base structure exhibited an IC_50_ value similar to most of its derivatives, suggesting that the synthesized derivatives were largely ineffective against the tested Gram-negative strain, except for variants. This may be due to the unique outer membrane of Gram-negative cells, which can limit compounds accumulation inside the cell.^30^

The best antimicrobial activity against the *P. aeruginosa* strain was obtained for 4c (2,4,5-trimethoxyphenyl), 4o (4-chlorophenyl), and 4d (2,3,4-trimethoxyphenyl). The trimethoxyphenyl group may contribute to the antimicrobial activity against Gram-negative bacteria, possibly through effects reported in the literature for methoxy-substituted aromatic derivatives.^31,32^ One of the important consequences of the present study was the improved effect of compound 4o against *P. aeruginosa* as compared with the related compounds from our previous series of *p*-methyl-*m*-trifluoromethyl [https://doi.org/10.1080/14756366.2025.2513406]. Although the structural modifications employed in this series resulted in improved antibacterial efficacy, the present data set did not allow us to conclude whether this effect was mainly due to the modified heterocyclic framework or the *p*-chlorophenyl group or the synergistic effect of both these structural features. Therefore, more analogues will be required to delineate a more comprehensive structure–activity relationship.

The antifungal activity against the *C. albicans* strain (Fig. 3C), measured by IC_50_, did not show significant variations between the derivatives and the solvent used (*p* > 0.05), thus the antifungal activity was practically generated by the solvent used. The base compound led to cell proliferation compared to the solvent control. The best antimicrobial activity against the *C. albicans* strain was obtained for 4r (4-(trifluoromethyl)phenyl), 4j (furan-2-yl), and 4c (2,4,5-trimethoxyphenyl), even though the values were not significantly different from the solvent, the IC_50_ values were significantly lower compared to the base structure (*p* < 0.0001).

The ratio between the minimum microbicidal (MBC) and inhibitory (MIC) concentration could indicate if a certain compound exhibits a microbiostatic (>4) or a microbicidal (≤4) effect.^33^

In case of *S. aureus*, the ratio between the MBC and MIC values were ≤4 for the compounds 4a, 4c, 4d, 4e, 4f, 4k, 4m and 4r, indicating a bactericidal effect, while for the compounds 4b, 4i, 4j, 4l, 4n, 4o, 4p, 4s and 4t were >4, suggesting a bacteriostatic effect of the obtained compounds.

In case of *P, aeruginosa*, the compounds 4a–n and 4p–4t, were bactericidal effect, while the compound 4o was bacteriostatic.

For *C. albicans*, the compounds 4a, 4b, 4e, 4f, 4k, 4m, 4r and 4t, were fungicidal, while the compounds 4c, 4d, 4l, 4n and 4s fungistatic.

However, according to the Table 1 the MMC values of all the compounds are similar to those of DMSO, which means that the microbicidal activity could be also due to DMSO.

Microorganisms have the ability to evolve through self-conservation mechanisms, leading to the formation of biofilms, consisting of adherent microbial communities, protected by a complex, self-produced matrix from host defenses and antimicrobial treatments. They are frequently present in both chronic and acute wounds, representing a factor for delayed wound healing.^34,35^ This biofilm formation can lead to persistent and recurrent skin infections.^36^

The development of new compounds capable of inhibiting microbial adherence could be one of the solutions for both increasing antibiotic resistance and healing chronic wounds. In this regard, the influence of newly synthesized compounds on microbial adherence, the initial stage of microbial biofilm development, was evaluated. The values of the minimum inhibitory concentrations for microbial adherence (MICMA) obtained are presented in Table 1. Compounds with MICMA values lower than MIC are considered active in inhibiting microbial adherence, without interfering with the microbial growth. Most substances prevent the adherence of *S. aureus* cells. The compounds with higher activity in relation to both the base structure (4m) and DMSO include 4c, 4n, 4s, 4a, 4d, 4j, 4l, 4p, 4r, 4t, 4e, 4h. Even though the antimicrobial activity was weak against the *P. aeruginosa* strain, the newly synthesized compounds 4o, 4n, 4d, 4h, 4p, 4r significantly inhibited microbial adherence. Compounds with higher activity against *C. albicans* adherence, both in terms of the base structures (4m) and DMSO, include 4k, 4e, and 4a.

Although the values of the obtained antimicrobial quantitative parameters are in the mg mL^−1^ range and are higher compared to those reported for conventional systemic antibiotics, these results must be interpreted in the context of potential topical applications. In the case of local administration, such as wound treatment, significantly higher concentrations can be achieved directly at the site of application, without the pharmacokinetic constraints associated with systemic therapy.^37–40^ In this context, the obtained results support the utility of the compounds for further studies on optimisation and evaluation of the structure–activity relationship. The proposed mechanisms are literature-based and were not experimentally investigated in the present study, these aspects will be explored in future studies.

A comparison with our previously reported trifluoromethylated pyrrol-2-one derivatives^17^ indicates that the introduction of an additional methyl substituent on the aniline precursor did not substantially modify the overall antimicrobial spectrum of the scaffold. In both series, the highest activity was observed against *Staphylococcus aureus*, whereas activity against *Pseudomonas aeruginosa* was more limited and antifungal effects against *Candida albicans* remained weak. These findings suggest that the trifluoromethylated pyrrol-2-one core constitutes the main structural determinant of antimicrobial activity, while peripheral substituents primarily modulate potency.

Nevertheless, the present series exhibited a distinct structure–activity profile, indicating that the additional methyl group subtly alters the electronic and lipophilic properties of the scaffold and influences the contribution of the aldehyde-derived substituent to biological activity. Moreover, several compounds from both studies inhibited microbial adherence at concentrations lower than those required for growth inhibition, suggesting a potential effect on early stages of biofilm formation independent of direct antimicrobial action.

Although the structural diversity of the two libraries precludes a direct compound-to-compound comparison, the combined results demonstrate that small modifications in the substitution pattern of the aniline precursor can influence both the biological profile and the overall performance of this class of trifluoromethyl-containing pyrrol-2-one derivatives.

### Hemolytic activity

**Fig. 4:**
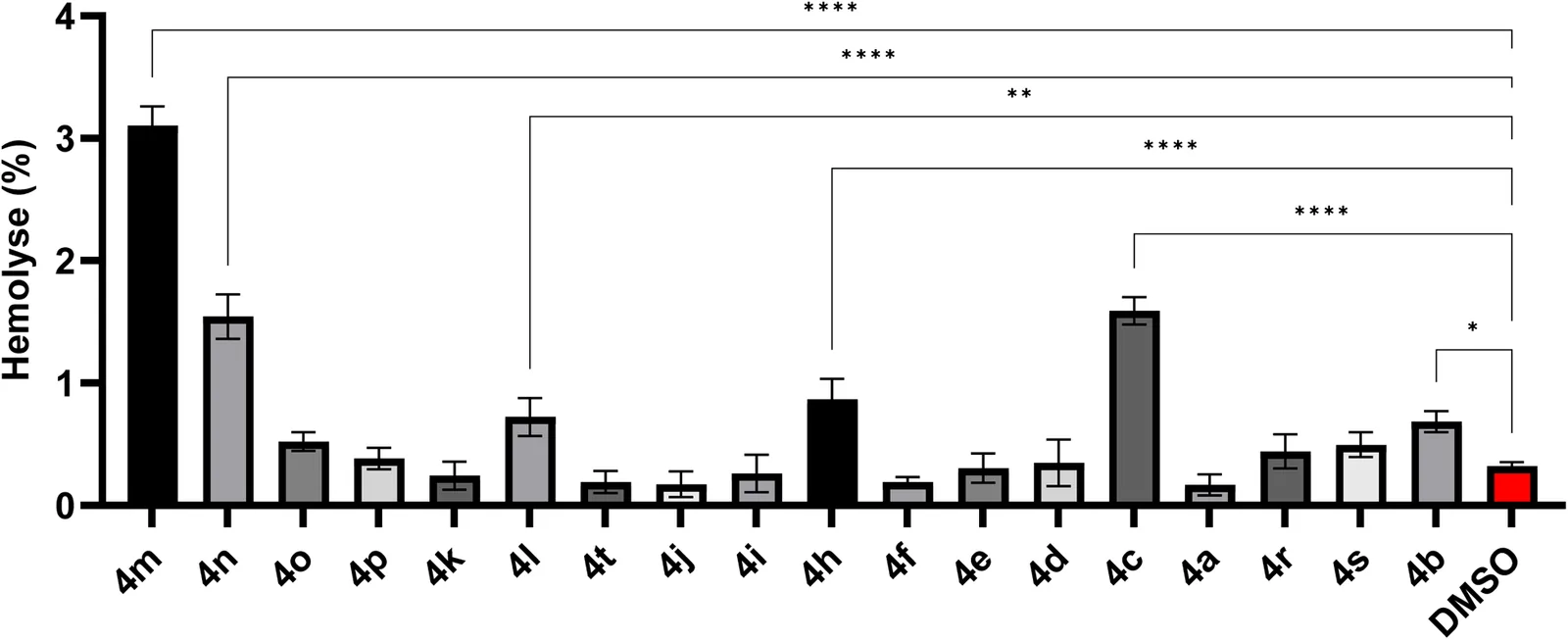
Hemolysis of the erythrocyte suspension generated by 2.5 mg mL^−1^ for all *p*-methyl-*m*-trifluoromethyl compounds. The results are expressed as: mean ± SD, each experiment was performed in four replicates. The standard two-way ANOVA test – the Sidak multiple comparisons test highlighted significant differences between the derivatives and DMSO (*p < 0.05, **p < 0.01, ***p < 0.001, ****p < 0.0001).

The *p*-methyl-*m*-trifluoromethyl compounds generally showed significantly lower hemolytic activity (<5%) than the base structure (4m). Since a hemolysis of up to 5% is allowed for biomaterials, both the base structure and its derivatives are safe for use as active principles in formulations with topical applications, demonstrating their biocompatibility within the evaluated bioactive concentration range. From Fig. 4, it can be observed that some of the compounds generated significantly higher hemolysis compared to the solvent used: 4b (*p* < 0.05), 4l (*p* < 0.01), 4c, 4h, 4n, and 4m (*p* < 0.0001), but the values are still significantly below the 5% threshold.

From Fig. 4 it can be observed that hemolysis decreases in the following order: 4m > 4c > 4n > 4h > 4l > 4b > 4o > 4s > 4r > 4p > 4d > DMSO > 4e > 4i > 4k > 4f > 4t > 4j > 4a.

### Biocompatibility

The cytotoxicity of the newly synthesized compounds was assessed by examining the integrity of HaCaT cell membranes. The MTT colorimetric assay was used as an indirect indicator of cell viability, which evaluates the capacity of viable cells to convert a colorless tetrazolium salt into blue formazan. This method is commonly employed *in vitro* to predict toxicity and skin irritation.^41^ Keratinocytes are particularly relevant for skin irritation studies because they are the first living cells exposed to external compounds. HaCaT cells provide a consistent supply of uniform cells and are known for their good correlation with *in vivo* irritability data in humans.^42^ Therefore, it is essential to thoroughly investigate cellular responses to chemical compound exposure. Our approach is consistent with previous studies that demonstrate the ability of keratinocytes to predict dermal irritation in humans.^43,44^

**Fig. 5:**
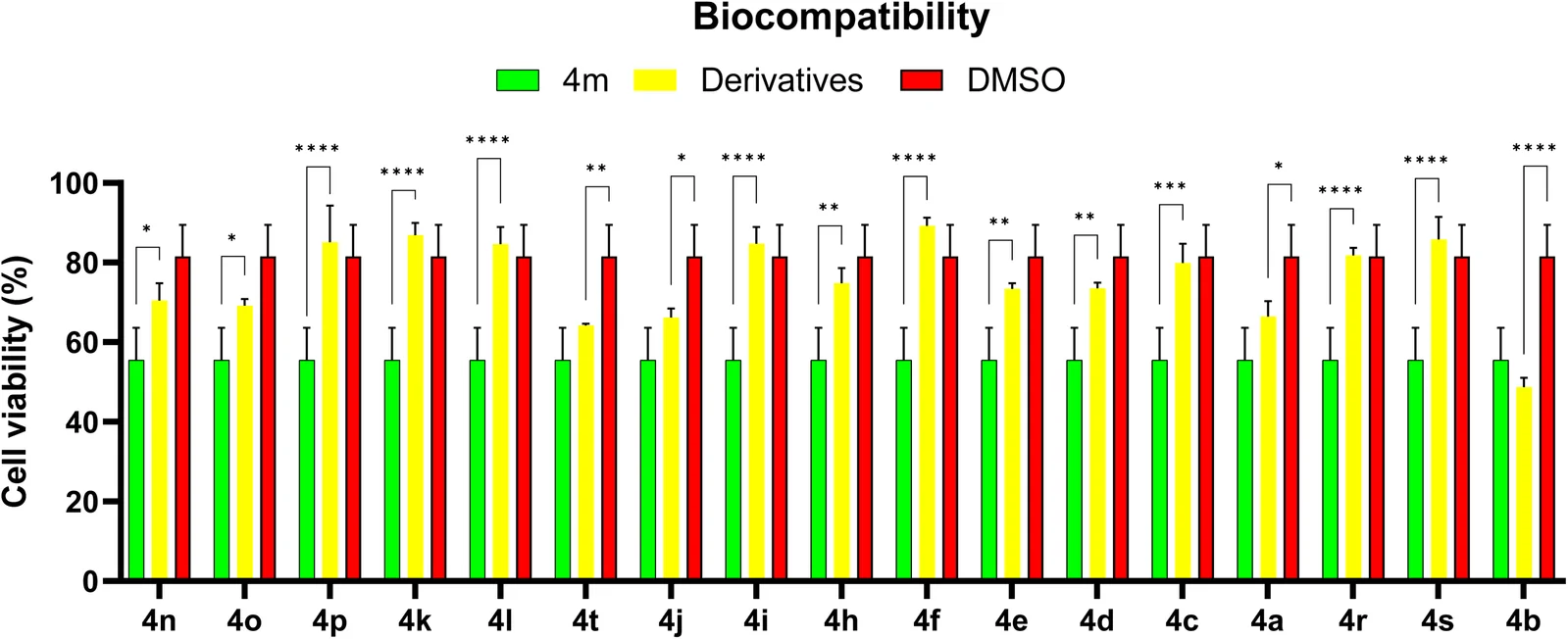
Biocompatibility evaluation by the MTT assay under standard cultivation conditions of the HaCaT human keratinocyte cell line.

The most cytotoxic compounds on HaCaT keratinocytes were 4b, 4m, 4t, 4j, 4a, and 4o. The rest of the derivatives were more biocompatible compared to the compound with the basic structure (4m) (Fig. 5).

The order of the cytotoxicity depending on the radical groups linked to the core structure is the following: 4b > 4m > 4t > 4j > 4a > 4o > 4n > 4e > 4d > 4h > 4c > DMSO > 4r > 4l > 4i > 4p > 4s > 4k > 4f. The compounds 4r (4-(trifluoromethyl)phenyl), 4l (4-(*tert*-butyl)phenyl), 4i (4-methoxyphenyl), 4p (4-bromophenyl), 4s (3,5-bis(trifluoromethyl)phenyl), 4k (4-*iso*propylphenyl) and 4f (2,5-dimethoxyphenyl) have shown the best cytocompatibility, although statistically insignificant compared to DMSO (*p* > 0.05).

Remarkably, the highly biocompatible compounds, 4i, 4n, 4p, 4l, 4o, 4s, and 4t have also shown significant antimicrobial activity against *S. aureus*. Additionally, 4s and 4o have also displayed enhanced antimicrobial effectiveness against *P. aeruginosa.* Therefore, we can conclude that the compounds 4s, and 4o demonstrated a broad spectrum of antibacterial activity including both Gram-positive and Gram-negative strains, while maintaining low cytotoxicity. These compounds proved also hemocompatible, making them suitable for use in topical treatments aimed to prevent infections in acute or chronic wounds.

**Fig. 6:**
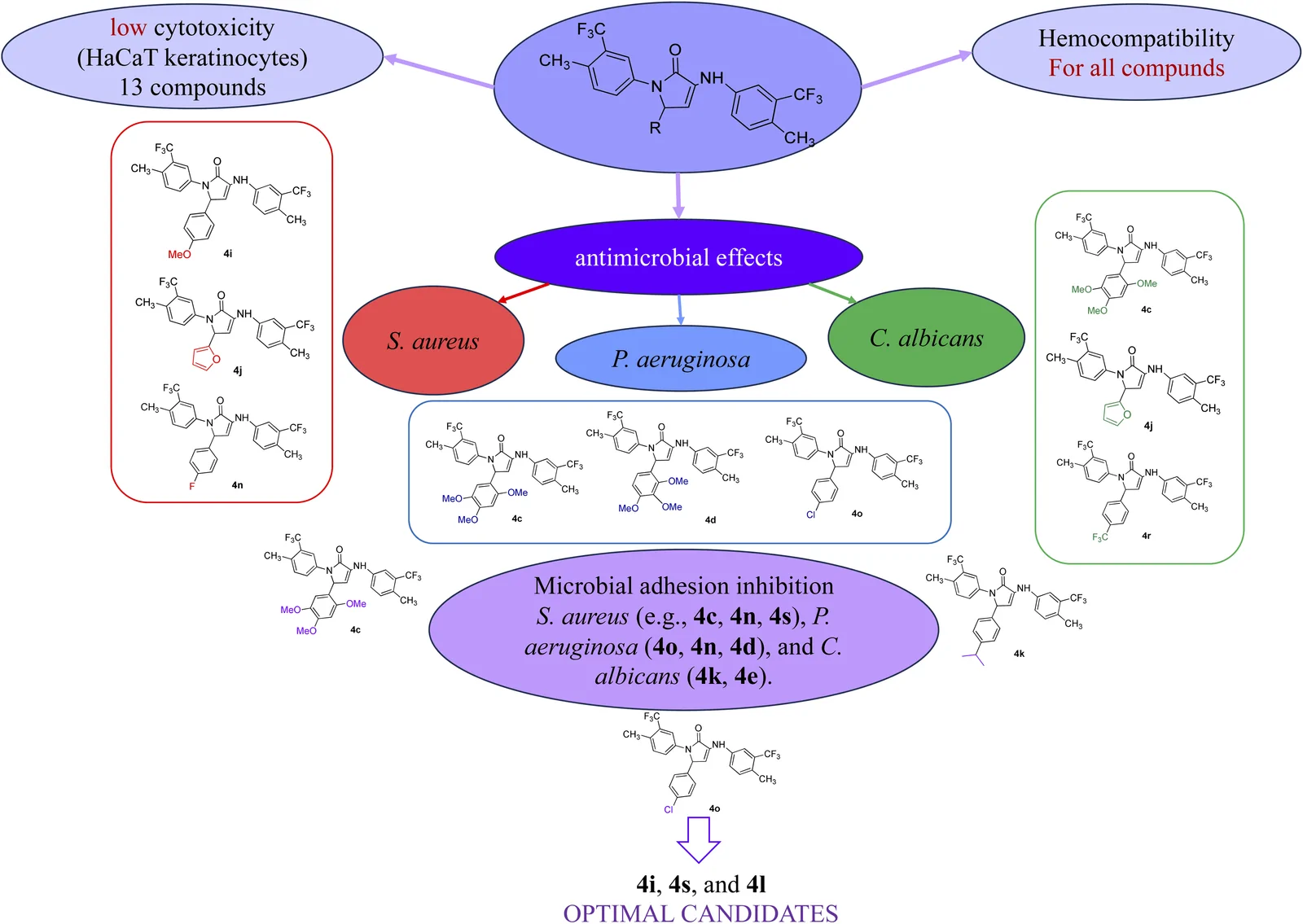
Most significant results representation.

The summary of the obtained results is presented in Fig. 6.

## Conclusions

In conclusion, a series of nineteen new trifluoromethyl- and methyl-substituted dihydro-pyrrol-2-one derivatives was successfully synthesized through a one-pot three-component reaction involving substituted anilines, aldehydes, and pyruvic acid. The methodology proved to be operationally simple and efficient, providing rapid access to structurally diverse heterocyclic scaffolds of potential medicinal interest.

One of the most significant findings of this study concerns the influence of the aniline substitution pattern on the reaction outcome. In contrast to our previously reported trifluoromethylated systems, which afforded both pyrrol-2-one and furan-type products depending on the aldehyde employed, the introduction of an additional methyl group on the trifluoromethyl-substituted aniline resulted exclusively in the formation of pyrrol-2-one derivatives. These observations indicate that, besides the aldehyde component, the electronic and steric characteristics of the aniline precursor also contribute to directing the reaction pathway and chemoselectivity. When considered together with our previous investigations on related electron-withdrawing substituted aniline systems, the present results provide additional insight into the factors governing this multicomponent transformation and contribute to the gradual elucidation of its underlying mechanism.

The structures of all synthesized compounds were confirmed by spectroscopic and spectrometric methods, complemented by single-crystal X-ray diffraction analysis. *In silico* ADMET evaluation revealed generally comparable pharmacokinetic and safety profiles across the series, identifying the pyrrol-2-one scaffold as a suitable platform for further optimization. The molecular docking predictions should be interpreted cautiously because they do not fully capture protein flexibility, solvent effects, or the complexity of biological systems and therefore require experimental validation.

Biological evaluation demonstrated moderate to good antibacterial activity, particularly against Gram-positive strains, together with low cytotoxicity and excellent hemocompatibility. Several derivatives also displayed the ability to inhibit microbial adherence at concentrations below those required for growth inhibition, suggesting a potential antibiofilm effect. Among the investigated compounds, derivatives 4s and 4o emerged as the most promising overall candidates by combining antibacterial activity, adherence inhibition, and favorable biocompatibility profiles. Collectively, these findings support the continued exploration of trifluoromethylated pyrrol-2-one derivatives as potential leads for the development of new antimicrobial agents, particularly for topical anti-infective applications. Future studies will focus on the most promising compounds and assess their antimicrobial efficacy against clinically relevant multidrug-resistant isolates to better define their therapeutic potential.

## Experimental

Analytical thin-layer chromatography was performed with commercial silica gel plates 60 F254 (Merck Darmstadt, Germany) and visualized with UV light (*λ*_max_ = 254 or 365 nm).

The NMR spectra included in this study were recorded on Bruker Avance NEO 400 spectrometer equipped with either 5 mm four nuclei direct detection *z*-gradient probe (H,C,F,Si-QNP) and 5 mm inverse detection multinuclear *z*-gradient probe respectively. Proton and carbon chemical shifts are reported in *δ* units (ppm) relative to the residual solvent signal (ref: DMSO-*d*_6_^1^H, 2.51 ppm and ^13^C, 39.47 ppm). ^1^H, ^13^C, ^19^F, H,H-COSY, H,C-HSQC and H,C-HMBC experiments were recorded using standard pulse sequences as delivered by Bruker with TopSpin 4.0.8 spectrometer control and processing software. The ^15^N chemical shifts were obtained as projections from the 2D indirectly detected H,N-HMBC spectra and are referred to external liquid ammonia (0.0 ppm) using as external standard nitromethane (380.2 ppm).

IR spectra were recorded on a Shimadzu IRTracer-100 instrument (Shimadzu U.S.A. Manufacturing, Inc., Canby, OR, USA). The melting point of the compounds measured on a MEL-TEMP capillary melting point apparatus from ambient temperature up to 400 °C. All commercially available products were used without further purification unless otherwise specified.

Mass spectra were acquired using a MALDI-TOF/TOF mass spectrometer (rapifleX™, Bruker Daltonics). Sample preparation was carried out as previously described.^13^ Briefly, a mixture of α-cyano-4-hydroxycinnamic acid (CHCA) matrix and sample solution at a 2 : 1 ratio was deposited onto a ground steel MALDI target plate (1 µL per spot) using the dried-droplet method. The loaded plate was allowed to dry at room temperature prior to MALDI-MS analysis. Data acquisition and instrument optimization were performed using FlexControl 4.0 software (Bruker Daltonics) under the following conditions: positive reflector mode, acceleration voltage of 20 kV, and a mass range of *m*/*z* 100–1600 Da. Laser power was set to 60–80% of the maximum, and 500 laser shots were accumulated per acquisition to generate the final mass spectrum. Spectra were processed with FlexAnalysis 4.0 software (Bruker Daltonics). External calibration was conducted using a peptide mixture standard supplied by Bruker Daltonics.

### General procedure for the syntheses of compounds 4a–t

Aldehydes 2a–t (1 mmol) were solubilized in a minimum amount of ethanol. *p*-CH_3_, *m*-CF_3_-aniline 1 (1 mmol) was dissolved in a minimum amount of ethanol and added to the system. A mixture containing pyruvic acid 3 (1.3 mmol) and TFA (20 µL) as catalyst, in ethanol was then added and the resulting mixture was then left to reflux for 24 hours. The required products were obtained by filtering out the resulting suspension and washing the solid with ethanol. Dichloromethane and ethanol were used to facilitate recrystallization.

High-performance liquid chromatography (HPLC) analyses were performed using an Agilent 1260 Infinity Series system equipped with a UV-vis diode-array detector (DAD). Separation was achieved on a Bischoff ProntoSIL EuroBOND C18 column (5 µm, 4 × 125 mm). The mobile phase consisted of (A) 0.1% aqueous formic acid and (B) acetonitrile, applied under gradient elution at a flow rate of 1 mL min^−1^. All compounds exhibited a purity greater than 95%, as determined by HPLC at 280 nm. Chromatograms of representative compounds are provided in the SI.

### 5-(4-Hydroxy-3,5-dimethoxyphenyl)-1-(4-methyl-3-(trifluoromethyl)phenyl)-3-((4-methyl-3-(trifluoromethyl)phenyl)amino)-1*H*-pyrrol-2(5*H*)-one (4a)

Yellow solid; *η* = 30%, mp = 181–182 °C; IR(ATR) √(cm^−1^): 3672, 3317, 3259, 2904, 2357, 2337, 1682, 1647, 1593, 1508, 1459, 1385, 1323, 1219, 1153, 1103, 1072, 1045, 813, 640, 536.

^1^H NMR (DMSO-*d*_6_, 400.1 MHz, *δ* (ppm)): 2.36 (6H, bs, 2×CH_3_), 3.67 (6H, s, 2×OCH_3_-21), 6.01 (1H, d, ^3^*J* = 3 Hz, H-5), 6.38 (1H, d, ^3^*J* = 3 Hz, H-4), 6.54 (2H, s, H-20, 24), 7.31 (1H, d, ^3^*J* = 8 Hz, H-9), 7.38 (1H, d, ^3^*J* = 8 Hz, H-15), 7.46 (1H, dd, ^3^*J* = 8.3 Hz, ^4^*J* = 2 Hz, H-8), 7.66 (1H, d, ^4^*J* = 2 Hz, H-12), 7.75 (1H, dd, ^3^*J* = 8.3 Hz, ^4^*J* = 2 Hz, H-14), 8.03 (1H, d, ^4^*J* = 2 Hz, H-18), 8.38 (1H, s, OH), 8.46 (1H, s, NH).

^13^C NMR (DMSO-*d*_6_, 100.6 MHz, *δ* (ppm)): 17.8 (q, ^4^*J*_C,F_ = 1.5 Hz, CH_3_-10), 18.1 (q, ^4^*J*_C,F_ = 1.5 Hz, CH_3_-16), 55.9 (OCH_3_-21, 23), 62.7 (CH-5), 104.5 (CH-20, 24), 110.9 (CH-4), 114.4 (q, ^3^*J*_C,F_ = 6.0 Hz, CH-12), 118.6 (q, ^3^*J*_C,F_ = 6.0 Hz, CH-18), 119.3 (CH-8), 124.2 (q, ^1^*J*_C,F_ = 274 Hz, CF_3_-11), 124.4 (q, ^1^*J*_C,F_ = 274 Hz, CF_3_-17), 124.9 (CH-14), 126.6 (C-10), 126.7 (C-19), 127.4 (q, ^1^*J*_C,F_ = 30 Hz, C-17), 127.8 (q, ^1^*J*_C,F_ = 30 Hz, C-11), 131.5 (C-3), 131.7 (C-16), 132.5 (CH-15), 132.9 (CH-9), 135.2 (C-13), 135.3 (C-22), 140.3 (C-7), 148.1 (C-21, 23), 166.3 (CO-2).

^15^N NMR (DMSO-*d*_6_, 40.5 MHz, *δ* (ppm)): 84.8 (NH), 142.2 (N).

^19^F NMR (DMSO-*d*_6_, 376.5 MHz, *δ* (ppm)): −60.47, −60.42.

HRMS (MALDI-TOF/TOF) *m*/*z*: [M + H]^+^ calcd for C_28_H_25_F_6_N_2_O_4_ 567.1718; found 567.1718.

### 5-(3-Hydroxy-4-methoxyphenyl)-1-(4-methyl-3-(trifluoromethyl)phenyl)-3-((4-methyl-3-(trifluoromethyl)phenyl)amino)-1*H*-pyrrol-2(5*H*)-one (4b)

White solid; *η* = 71%, mp = 211–212 °C; IR(ATR) √(cm^−1^): 3850, 3726, 3625, 3527, 3448, 3321, 2970, 1735, 1674, 1647, 1545, 1507, 1414, 1366, 1284, 1217, 1168, 1117, 1101, 1048, 1030, 890, 842, 829, 780, 763, 715, 661, 617, 540, 518, 457.

^1^H NMR (DMSO-*d*_6_, 400.1 MHz, *δ* (ppm)): 2.35 (6H, bs, 2×CH_3_), 3.69 (3H, s, OCH_3_-22), 6.00 (1H, d, ^3^*J* = 3 Hz, H-5), 6.35 (1H, d, ^3^*J* = 2 Hz, H-4), 6.60 (1H, d, ^4^*J* = 2 Hz, H-20), 6.75 (1H, dd, ^3^*J* = 8.0 Hz, ^4^*J* = 2.0 Hz, H-24), 6.83 (1H, d, ^3^*J* = 8.0 Hz, H-23), 7.30 (1H, d, ^3^*J* = 8 Hz, H-9), 7.38 (1H, d, ^3^*J* = 8 Hz, H-15), 7.44 (1H, dd, ^3^*J* = 8.3 Hz, ^4^*J* = 2 Hz, H-8), 7.64 (1H, d, ^4^*J* = 2 Hz, H-12), 7.68 (1H, dd, ^3^*J* = 8.3 Hz, ^4^*J* = 2 Hz, H-14), 8.09 (1H, d, ^4^*J* = 2 Hz, H-18), 8.46 (1H, s, NH), 8.99 (1H, s, OH).

^13^C NMR (DMSO-*d*_6_, 100.6 MHz, *δ* (ppm)): 17.8 (q, ^4^*J*_C,F_ = 1.5 Hz, CH_3_-10), 18.1 (q, ^4^*J*_C,F_ = 1.5 Hz, CH_3_-16), 55.4 (OCH_3_-22), 62.0 (CH-5), 111.2 (CH-4), 112.2 (CH-23), 113.2 (CH-20), 114.5 (q, ^3^*J*_C,F_ = 6.0 Hz, CH-12), 118.1 (q, ^3^*J*_C,F_ = 6.0 Hz, CH-18), 118.1 (CH-24), 119.2 (CH-8), 124.2 (q, ^1^*J*_C,F_ = 274 Hz, CF_3_-11), 124.4 (q, ^1^*J*_C,F_ = 274 Hz, CF_3_-17), 124.5 (CH-14), 126.6 (C-10), 127.4 (q, ^1^*J*_C,F_ = 30 Hz, C-17), 127.8 (q, ^1^*J*_C,F_ = 30 Hz, C-11), 129.2 (C-19), 131.2 (C-3), 131.6 (C-16), 132.5 (CH-15), 132.9 (CH-9), 135.3 (C-13), 140.3 (C-7), 146.7 (C-21), 147.4 (C-21), 166.4 (CO-2).

^15^N NMR (DMSO-*d*_6_, 40.5 MHz, *δ* (ppm)): 84.6 (NH), 142.2 (N).

^19^F NMR (DMSO-*d*_6_, 376.5 MHz, *δ* (ppm)): −60.48.

HRMS (MALDI-TOF/TOF) *m*/*z*: [M + H]^+^ calcd for C_27_H_23_F_6_N_2_O_3_ 537.1612; found 537.1606.

### 1-(4-Methyl-3-(trifluoromethyl)phenyl)-3-((4-methyl-3-(trifluoromethyl)phenyl)amino)-5-(2,4,5-trimethoxyphenyl)-1*H*-pyrrol-2(5*H*)-one (4c)

Orange solid; *η* = 44%, mp = 183–184 °C; IR(ATR) √(cm^−1^): 3675, 3362, 3271, 2971, 2907, 2360, 2340, 1693, 16 653, 1593, 1540, 1516, 1379, 1322, 1303, 1260, 1145, 1050, 908, 817, 743, 652, 539.

^1^H NMR (DMSO-*d*_6_, 400.1 MHz, *δ* (ppm)): 2.34 (6H, bs, 2×CH_3_), 3.52 (3H, s, OCH_3_-22), 3.75 (3H, s, OCH_3_-23), 3.83 (3H, s, OCH_3_-20), 6.23 (1H, d, ^3^*J* = 3 Hz, H-5), 6.29 (1H, d, ^3^*J* = 3 Hz, H-4), 6.49 (1H, s, H-21), 6.68 (1H, s, H-24), 7.30 (1H, d, ^3^*J* = 8 Hz, H-9), 7.38 (1H, d, ^3^*J* = 8 Hz, H-15), 7.44 (1H, dd, ^3^*J* = 8.3 Hz, ^4^*J* = 2 Hz, H-8), 7.64 (1H, d, ^4^*J* = 2 Hz, H-12), 7.73 (1H, dd, ^3^*J* = 8.3 Hz, ^4^*J* = 2 Hz, H-14), 7.96 (1H, d, ^4^*J* = 2 Hz, H-18), 8.42 (1H, s, NH).

^13^C NMR (DMSO-*d*_6_, 100.6 MHz, *δ* (ppm)): 17.9 (q, ^4^*J*_C,F_ = 1.5 Hz, CH_3_-10), 18.1 (q, ^4^*J*_C,F_ = 1.5 Hz, CH_3_-16), 55.7 (OCH_3_-22), 56.2 (OCH_3_-23), 56.6 (OCH_3_-20), 56.7 (CH-5), 98.4 (CH-24), 110.0 (CH-4), 111.2 (CH-21), 114.4 (q, ^3^*J*_C,F_ = 6.0 Hz, CH-12), 114.6 (C-19), 117.9 (q, ^3^*J*_C,F_ = 6.0 Hz, CH-18), 119.2 (CH-8), 124.3 (q, ^1^*J*_C,F_ = 274 Hz, CF_3_-11), 124.2 (CH-14), 124.4 (q, ^1^*J*_C,F_ = 274 Hz, CF_3_-17), 126.6 (C-10), 127.4 (q, ^1^*J*_C,F_ = 30 Hz, C-17), 127.8 (q, ^1^*J*_C,F_ = 30 Hz, C-11), 131.4 (C-16), 132.0 (C-3), 132.6 (CH-15), 132.9 (CH-9), 135.4 (C-13), 140.4 (C-7), 143.0 (C-23), 149.6 (C-22), 151.8 (C-20), 166.5 (CO-2).

^15^N NMR (DMSO-*d*_6_, 40.5 MHz, *δ* (ppm)): 84.9 (NH), 141.8 (N).

^19^F NMR (DMSO-*d*_6_, 376.5 MHz, *δ* (ppm)): −60.53, −60.48.

HRMS (MALDI-TOF/TOF) *m*/*z*: [M + H]^+^ calcd for C_29_H_27_F_6_N_2_O_4_ 581.1874; found 581.1870.

### 1-(4-Methyl-3-(trifluoromethyl)phenyl)-3-((4-methyl-3-(trifluoromethyl)phenyl)amino)-5-(2,3,4-trimethoxyphenyl)-1*H*-pyrrol-2(5*H*)-one (4d)

Yellow solid; *η* = 55%, mp = 161–163 °C; IR(ATR) √(cm^−1^): 3730, 3625, 3529, 3503, 3317, 2970, 1734, 1673, 1495, 1432, 1369, 1218, 1131, 1095, 1049, 1006, 901, 829, 774, 691, 669, 527, 453, 400.

^1^H NMR (DMSO-*d*_6_, 400.1 MHz, *δ* (ppm)): 2.34 (6H, bs, 2×CH_3_), 3.70 and 3.71 (6H, s, OCH_3_-21 and OCH_3_-22), 3.89 (3H, s, OCH_3_-20), 6.19 (1H, d, ^3^*J* = 3 Hz, H-5), 6.35 (1H, d, ^3^*J* = 3 Hz, H-4), 6.69 (2H, s, H-23 and H-24), 7.29 (1H, d, ^3^*J* = 8 Hz, H-9), 7.38 (1H, d, ^3^*J* = 8 Hz, H-15), 7.45 (1H, dd, ^3^*J* = 8.3 Hz, ^4^*J* = 2 Hz, H-8), 7.66 (1H, d, ^4^*J* = 2 Hz, H-12), 7.74 (1H, dd, ^3^*J* = 8.3 Hz, ^4^*J* = 2 Hz, H-14), 7.98 (1H, d, ^4^*J* = 2 Hz, H-18), 8.45 (1H, s, NH).

^13^C NMR (DMSO-*d*_6_, 100.6 MHz, *δ* (ppm)): 17.8 (q, ^4^*J*_C,F_ = 1.5 Hz, CH_3_-10), 18.1 (q, ^4^*J*_C,F_ = 1.5 Hz, CH_3_-16), 55.7 (OCH_3_-21), 57.2 (CH-5), 60.1 (OCH_3_-22), 61.5 (OCH_3_-20), 108.4 (CH-23), 110.2 (CH-4), 114.5 (q, ^3^*J*_C,F_ = 6.0 Hz, CH-12), 117.7 (q, ^3^*J*_C,F_ = 6.0 Hz, CH-18), 119.2 (CH-8), 121.6 (CH-24), 124.2 (q, ^1^*J*_C,F_ = 274 Hz, CF_3_-11), 124.1 (CH-14), 124.4 (q, ^1^*J*_C,F_ = 274 Hz, CF_3_-17), 126.6 (C-10), 127.4 (q, ^1^*J*_C,F_ = 30 Hz, C-17), 127.8 (q, ^1^*J*_C,F_ = 30 Hz, C-11), 131.5 (C-3), 131.9 (C-16), 132.6 (CH-15), 132.9 (CH-9), 135.4 (C-13), 140.3 (C-7), 141.6 (C-21), 151.7 (C-20), 153.1 (C-22), 166.5 (CO-2).

^15^N NMR (DMSO-*d*_6_, 40.5 MHz, *δ* (ppm)): 85.1 (NH), 141.4 (N).

^19^F NMR (DMSO-*d*_6_, 376.5 MHz, *δ* (ppm)): −60.61, −60.50.

HRMS (MALDI-TOF/TOF) *m*/*z*: [M + H]^+^ calcd for C_29_H_27_F_6_N_2_O_4_ 581.1874; found 581.1881.

### 1-(4-Methyl-3-(trifluoromethyl)phenyl)-3-((4-methyl-3-(trifluoromethyl)phenyl)amino)-5-(3,4,5-trimethoxyphenyl)-1*H*-pyrrol-2(5*H*)-one (4e)

Yellow crystals; *η* = 80%, mp = 156–159 °C; IR(ATR) √(cm^−1^): 3729, 3626, 3528, 3504, 3448, 3319, 3005, 2970, 2940, 1749, 1734, 1657, 1592, 1537, 1505, 1463, 1381, 1322, 1277, 1229, 1105, 1050, 1006, 889, 823, 776, 673, 518, 457, 419, 401.

^1^H NMR (DMSO-*d*_6_, 400.1 MHz, *δ* (ppm)): 2.36 (6H, bs, 2×CH_3_), 3.59 (3H, s, OCH_3_-22), 3.69 (6H, s, 2×OCH_3_-21), 6.07 (1H, d, ^3^*J* = 3 Hz, H-5), 6.40 (1H, d, ^3^*J* = 3 Hz, H-4), 6.61 (2H, s, H-20), 7.30 (1H, d, ^3^*J* = 8 Hz, H-9), 7.38 (1H, d, ^3^*J* = 8 Hz, H-15), 7.46 (1H, dd, ^3^*J* = 8.3 Hz, ^4^*J* = 2 Hz, H-8), 7.66 (1H, d, ^4^*J* = 2 Hz, H-12), 7.74 (1H, dd, ^3^*J* = 8.3 Hz, ^4^*J* = 2 Hz, H-14), 8.08 (1H, d, ^4^*J* = 2 Hz, H-18), 8.47 (1H, s, NH).

^13^C NMR (DMSO-*d*_6_, 100.6 MHz, *δ* (ppm)): 17.8 (q, ^4^*J*_C,F_ = 1.5 Hz, CH_3_-10), 18.1 (q, ^4^*J*_C,F_ = 1.5 Hz, CH_3_-16), 55.8 (OCH_3_-21, 23), 59.8 (OCH_3_-22), 62.6 (CH-5), 104.2 (CH-20, 24), 110.6 (CH-4), 114.5 (q, ^3^*J*_C,F_ = 6.0 Hz, CH-12), 118.6 (q, ^3^*J*_C,F_ = 6.0 Hz, CH-18), 119.3 (CH-8), 124.2 (q, ^1^*J*_C,F_ = 274 Hz, CF_3_-11), 124.4 (q, ^1^*J*_C,F_ = 274 Hz, CF_3_-17), 124.8 (CH-14), 126.6 (C-10), 127.4 (q, ^1^*J*_C,F_ = 30 Hz, C-17), 127.8 (q, ^1^*J*_C,F_ = 30 Hz, C-11), 131.5 (C-3), 131.6 (C-16), 132.6 (C-19), 132.8 (CH-15), 132.9 (CH-9), 135.3 (C-13), 136.9 (C-22), 140.3 (C-7), 153.1 (C-21, 23), 166.4 (CO-2).

^15^N NMR (DMSO-*d*_6_, 40.5 MHz, *δ* (ppm)): 84.9 (NH), 141.4 (N).

^19^F NMR (DMSO-*d*_6_, 376.5 MHz, *δ* (ppm)): −60.52, −60.50.

HRMS (MALDI-TOF/TOF) *m*/*z*: [M + H]^+^ calcd for C_29_H_27_F_6_N_2_O_4_ 581.1874; found 581.1929.

### 5-(2,5-Dimethoxyphenyl)-1-(4-methyl-3-(trifluoromethyl)phenyl)-3-((4-methyl-3-(trifluoromethyl)phenyl)amino)-1*H*-pyrrol-2(5*H*)-one (4f)

White solid; *η* = 59%, mp = 166–168 °C; IR(ATR) √(cm^−1^): 3730, 3705, 3626, 3503, 3448, 3330, 3016, 2970, 2945, 1744, 1687, 1620, 1502, 1439, 1366, 1320, 1217, 1146, 1116, 1044, 879, 828, 776, 692, 670, 540, 517, 457, 418.

^1^H NMR (DMSO-*d*_6_, 400.1 MHz, *δ* (ppm)): 2.35 (6H, bs, 2×CH_3_), 3.57 (3H, s, OCH_3_-23), 3.82 (3H, s, OCH_3_-20), 6.28 (1H, d, ^3^*J* = 3 Hz, H-5), 6.32 (1H, d, ^3^*J* = 3 Hz, H-4), 6.46 (1H, d, ^4^*J*_H,H_ = 3 Hz, H-24), 6.79 (1H, dd, ^3^*J*_H,H_ = 9 Hz, ^4^*J*_H,H_ = 3 Hz, H-22), 6.98 (1H, d, ^3^*J*_H,H_ = 9 Hz, H-21), 7.30 (1H, d, ^3^*J* = 8 Hz, H-9), 7.39 (1H, d, ^3^*J* = 8 Hz, H-15), 7.43 (1H, dd, ^3^*J* = 8.3 Hz, ^4^*J* = 2 Hz, H-8), 7.64 (1H, d, ^4^*J* = 2 Hz, H-12), 7.72 (1H, dd, ^3^*J* = 8.3 Hz, ^4^*J* = 2 Hz, H-14), 7.98 (1H, d, ^4^*J* = 2 Hz, H-18), 8.45 (1H, s, NH).

^13^C NMR (DMSO-*d*_6_, 100.6 MHz, *δ* (ppm)): 17.8 (q, ^4^*J*_C,F_ = 1.5 Hz, CH_3_-10), 18.0 (q, ^4^*J*_C,F_ = 1.5 Hz, CH_3_-16), 55.2 (OCH_3_-23), 56.2 (OCH_3_-20), 56.8 (CH-5), 109.5 (CH-4), 112.6 (CH-24), 112.8 (CH-21), 113.5 (CH-22), 114.5 (q, ^3^*J*_C,F_ = 6.0 Hz, CH-12), 117.4 (q, ^3^*J*_C,F_ = 6.0 Hz, CH-18), 119.2 (CH-8), 123.8 (CH-14), 124.2 (q, ^1^*J*_C,F_ = 274 Hz, CF_3_-11), 124.4 (q, ^1^*J*_C,F_ = 274 Hz, CF_3_-17), 125.3 (C-19), 126.6 (C-10), 127.4 (q, ^1^*J*_C,F_ = 30 Hz, C-17), 127.7 (q, ^1^*J*_C,F_ = 30 Hz, C-11), 131.4 (C-16), 132.1 (C-3), 132.6 (CH-15), 132.9 (CH-9), 135.4 (C-13), 140.3 (C-7), 151.1 (C-20), 153.3 (C-23), 166.5 (CO-2).

^15^N NMR (DMSO-*d*_6_, 40.5 MHz, *δ* (ppm)): 85.0 (NH), 140.4 (N).

^19^F NMR (DMSO-*d*_6_, 376.5 MHz, *δ* (ppm)): −60.60, −60.50.

HRMS (MALDI-TOF/TOF) *m*/*z*: [M + H]^+^ calcd for C_28_H_25_F_6_N_2_O_3_ 551.1769; found 551.1761.

### 5-(3,4-Dimethoxyphenyl)-1-(4-methyl-3-(trifluoromethyl)phenyl)-3-((4-methyl-3-(trifluoromethyl)phenyl)amino)-1*H*-pyrrol-2(5*H*)-one (4g)

Yellow crystals; *η* = 35%, mp = 168–169 °C; IR(ATR) √(cm^−1^): 3732, 3706, 3626, 3500, 3448, 3326, 3016, 2972, 2946, 1746, 1685, 1620, 1500, 1440, 1364, 1322, 1215, 1146, 1116, 1045, 880, 825, 777, 692, 671, 540, 517, 457, 418.

^1^H NMR (DMSO-*d*_6_, 400.1 MHz, *δ* (ppm)): 2.35 (6H, bs, 2×CH_3_), 3.68 (3H, s, OCH_3_-22), 3.69 (3H, s, OCH_3_-21), 6.07 (1H, d, ^3^*J* = 3 Hz, H-5), 6.38 (1H, d, ^3^*J* = 3 Hz, H-4), 6.78 (1H, dd, ^3^*J*_H,H_ = 8 Hz, ^4^*J*_H,H_ = 2 Hz, H-24), 6.85 (1H, d, ^3^*J*_H,H_ = 8 Hz, H-21), 6.90 (1H, d, ^4^*J*_H,H_ = 2 Hz, H-20), 7.30 (1H, d, ^3^*J* = 8 Hz, H-9), 7.38 (1H, d, ^3^*J* = 8 Hz, H-15), 7.45 (1H, dd, ^3^*J* = 8.3 Hz, ^4^*J* = 2 Hz, H-8), 7.65 (1H, d, ^4^*J* = 2 Hz, H-12), 7.71 (1H, dd, ^3^*J* = 8.3 Hz, ^4^*J* = 2 Hz, H-14), 8.07 (1H, d, ^4^*J* = 2 Hz, H-18), 8.46 (1H, s, NH).

^13^C NMR (DMSO-*d*_6_, 100.6 MHz, *δ* (ppm)): 17.9 (q, ^4^*J*_C,F_ = 1.5 Hz, CH_3_-10), 18.1 (q, ^4^*J*_C,F_ = 1.5 Hz, CH_3_-16), 55.3 (OCH_3_-21), 54.4 (OCH_3_-22), 62.1 (CH-5), 110.7 (CH-20), 110.9 (CH-4), 111.9 (CH-23), 114.5 (q, ^3^*J*_C,F_ = 6.0 Hz, CH-12), 118.4 (q, ^3^*J*_C,F_ = 6.0 Hz, CH-18), 119.1 (CH-24), 119.3 (CH-8), 124.2 (q, ^1^*J*_C,F_ = 274 Hz, CF_3_-11), 124.4 (q, ^1^*J*_C,F_ = 274 Hz, CF_3_-17), 124.7 (CH-14), 126.6 (C-10), 127.3 (q, ^1^*J*_C,F_ = 30 Hz, C-17), 127.8 (q, ^1^*J*_C,F_ = 30 Hz, C-11), 129.1 (C-19), 131.5 (C-3), 131.6 (C-16), 132.5 (CH-15), 132.9 (CH-9), 135.3 (C-13), 140.3 (C-7), 148.4 (C-22), 148.8 (C-21), 165.3 (CO-2).

^15^N NMR (DMSO-*d*_6_, 40.5 MHz, *δ* (ppm)): 84.7 (NH), 142.4 (N).

^19^F NMR (DMSO-*d*_6_, 376.5 MHz, *δ* (ppm)): −60.48, −60.47.

HRMS (MALDI-TOF/TOF) *m*/*z*: [M + H]^+^ calcd for C_28_H_25_F_6_N_2_O_3_ 551.1769; found 551.1778.

### 5-(2-Methoxyphenyl)-1-(4-methyl-3-(trifluoromethyl)phenyl)-3-((4-methyl-3-(trifluoromethyl)phenyl)amino)-1*H*-pyrrol-2(5*H*)-one (4h)

White solid; *η* = 67%, mp = 169–170 °C; IR(ATR) √(cm^−1^): 3728, 3527, 3319, 2840, 1769, 1678, 1648, 1620, 1539, 1507, 1437, 1321, 1282, 1240, 1143, 1098, 1050, 1026, 888, 829, 774, 752, 710, 616, 539, 491, 452, 419.

^1^H NMR (DMSO-*d*_6_, 400.1 MHz, *δ* (ppm)): 2.34 (6H, bs, 2×CH_3_), 3.88 (3H, s, OCH_3_), 6.32 (1H, d, ^3^*J* = 3 Hz, H-5), 6.34 (1H, d, ^3^*J* = 3 Hz, H-4), 6.82 (1H, td, ^3^*J*_H,H_ = 8 Hz, ^4^*J*_H,H_ = 1 Hz, H-23), 6.92 (1H, dd, ^3^*J*_H,H_ = 8 Hz, ^4^*J*_H,H_ = 1.5 Hz, H-24), 7.05 (1H, dd, ^3^*J*_H,H_ = 8 Hz, ^4^*J*_H,H_ = 1 Hz, H-21), 6.82 (1H, td, ^3^*J*_H,H_ = 8 Hz, ^4^*J*_H,H_ = 1.5 Hz, H-22), 7.29 (1H, d, ^3^*J* = 8 Hz, H-9), 7.37 (1H, d, ^3^*J* = 8 Hz, H-15), 7.43 (1H, dd, ^3^*J* = 8.3 Hz, ^4^*J* = 2 Hz, H-8), 7.64 (1H, d, ^4^*J* = 2 Hz, H-12), 7.72 (1H, dd, ^3^*J* = 8.3 Hz, ^4^*J* = 2 Hz, H-14), 7.99 (1H, d, ^4^*J* = 2 Hz, H-18), 8.47 (1H, s, NH).

^13^C NMR (DMSO-*d*_6_, 100.6 MHz, *δ* (ppm)): 17.9 (q, ^4^*J*_C,F_ = 1.5 Hz, CH_3_-10), 18.0 (q, ^4^*J*_C,F_ = 1.5 Hz, CH_3_-16), 55.7 (OCH_3_), 56.4 (CH-5), 109.7 (CH-4), 111.5 (CH-21), 114.5 (q, ^3^*J*_C,F_ = 6.0 Hz, CH-12), 117.3 (q, ^3^*J*_C,F_ = 6.0 Hz, CH-18), 119.2 (CH-8), 120.9 (H-23), 123.6 (CH-14), 124.1 (C-19), 124.2 (q, ^1^*J*_C,F_ = 274 Hz, CF_3_-11), 124.4 (q, ^1^*J*_C,F_ = 274 Hz, CF_3_-17), 126.6 (C-10), 126.7 (CH-24), 127.4 (q, ^1^*J*_C,F_ = 30 Hz, C-17), 127.7 (q, ^1^*J*_C,F_ = 30 Hz, C-11), 129.2 (CH-22), 131.3 (C-16), 132.1 (C-3), 132.6 (CH-15), 132.9 (CH-9), 135.4 (C-13), 140.3 (C-7), 156.9 (C-20), 166.6 (CO-2).

^15^N NMR (DMSO-*d*_6_, 40.5 MHz, *δ* (ppm)): 84.7 (NH), 140.6 (N).

^19^F NMR (DMSO-*d*_6_, 376.5 MHz, *δ* (ppm)): −60.63, −60.50.

HRMS (MALDI-TOF/TOF) *m*/*z*: [M + H]^+^ calcd for C_27_H_23_F_6_N_2_O_2_ 521.1663; found 521.1669.

### 5-(4-Methoxyphenyl)-1-(4-methyl-3-(trifluoromethyl)phenyl)-3-((4-methyl-3-(trifluoromethyl)phenyl)amino)-1*H*-pyrrol-2(5*H*)-one (4i)

White solid; *η* = 58%, mp = 166–167 °C; IR(ATR) √(cm^−1^): 3730, 3528, 3321, 1765, 1707, 1648, 1543, 1506, 1392, 1346, 1284, 1243, 1101, 1048, 889, 829, 781, 745, 675, 576, 536, 508, 448, 419.

^1^H NMR (DMSO-*d*_6_, 400.1 MHz, *δ* (ppm)): 2.35 (6H, bs, 2×CH_3_), 3.68 (3H, s, OCH_3_), 6.09 (1H, d, ^3^*J* = 3 Hz, H-5), 6.36 (1H, d, ^3^*J* = 3 Hz, H-4), 6.85 (2H, d, ^3^*J*_H,H_ = 9 Hz, H-21), 7.20 (2H, d, ^3^*J*_H,H_ = 9 Hz, H-20), 7.29 (1H, d, ^3^*J* = 8 Hz, H-9), 7.37 (1H, d, ^3^*J* = 8 Hz, H-15), 7.69 (1H, dd, ^3^*J* = 8.3 Hz, ^4^*J* = 2 Hz, H-8), 7.65 (1H, d, ^4^*J* = 2 Hz, H-12), 7.68 (1H, dd, ^3^*J* = 8.3 Hz, ^4^*J* = 2 Hz, H-14), 8.07 (1H, d, ^4^*J* = 2 Hz, H-18), 8.48 (1H, s, NH).

^13^C NMR (DMSO-*d*_6_, 100.6 MHz, *δ* (ppm)): 17.9 (q, ^4^*J*_C,F_ = 1.5 Hz, CH_3_-10), 18.0 (q, ^4^*J*_C,F_ = 1.5 Hz, CH_3_-16), 54.9 (CH-5), 61.8 (OCH_3_), 110.0 (CH-4), 114.1 (CH-21), 114.5 (q, ^3^*J*_C,F_ = 6.0 Hz, CH-12), 118.3 (q, ^3^*J*_C,F_ = 6.0 Hz, CH-18), 119.2 (CH-8), 124.2 (q, ^1^*J*_C,F_ = 274 Hz, CF_3_-11), 124.4 (q, ^1^*J*_C,F_ = 274 Hz, CF_3_-17), 124.6 (CH-14), 126.9 (C-10), 127.3 (q, ^1^*J*_C,F_ = 30 Hz, C-17), 127.8 (q, ^1^*J*_C,F_ = 30 Hz, C-11), 128.2 (CH-20), 128.7 (C-19), 131.4 (C-3), 131.6 (C-16), 132.5 (CH-15), 132.9 (CH-9), 135.1 (C-13), 140.3 (C-7), 158.9 (C-22), 166.3 (CO-2).

^15^N NMR (DMSO-*d*_6_, 40.5 MHz, *δ* (ppm)): 87.2 (NH), 142.9 (N).

^19^F NMR (DMSO-*d*_6_, 376.5 MHz, *δ* (ppm)): −60.51, −60.50.

HRMS (MALDI-TOF/TOF) *m*/*z*: [M + H]^+^ calcd for C_27_H_23_F_6_N_2_O_2_ 521.1663; found 521.1669.

### 5-(Furan-2-yl)-1-(4-methyl-3-(trifluoromethyl)phenyl)-3-((4-methyl-3-(trifluoromethyl)phenyl)amino)-1*H*-pyrrol-2(5*H*)-one (4j)

White solid; *η* = 50%, mp = 165–166 °C; IR(ATR) √(cm^−1^): 3728, 3524, 3342, 2087, 1742, 1686, 1610, 1383, 1317, 1062, 1010, 825, 741, 601, 495.

^1^H NMR (DMSO-*d*_6_, 400.1 MHz, *δ* (ppm)): 2.37 and 2.40 (6H, bs, 2×CH_3_), 6.32 (1H, d, ^3^*J* = 3 Hz, H-5), 6.36 (1H, dd, ^3^*J* = 3 Hz, ^4^*J* = 1.8 Hz, H-22), 6.40 (1H, d, ^3^*J* = 3 Hz, H-4), 6.52 (1H, dd, ^3^*J* = 3 Hz, ^4^*J* = 0.8 Hz, H-23), 7.33 (1H, d, ^3^*J* = 8 Hz, H-9), 7.44 (1H, d, ^3^*J* = 8 Hz, H-15), 7.49 (1H, dd, ^3^*J* = 8.3 Hz, ^4^*J* = 2 Hz, H-8), 7.54 (1H, dd, ^3^*J* = 1.8 Hz, ^4^*J* = 0.8 Hz, H-21), 7.67 (1H, d, ^4^*J* = 2 Hz, H-12), 7.75 (1H, dd, ^3^*J* = 8.3 Hz, ^4^*J* = 2 Hz, H-14), 8.00 (1H, d, ^4^*J* = 2 Hz, H-18), 8.53 (1H, s, NH).

^13^C NMR (DMSO-*d*_6_, 100.6 MHz, *δ* (ppm)): 17.9 (q, ^4^*J*_C,F_ = 1.5 Hz, CH_3_-10), 18.1 (q, ^4^*J*_C,F_ = 1.5 Hz, CH_3_-16), 56.5 (CH-5), 106.7 (CH-4), 109.8 (CH-23), 110.5 (CH-22), 114.7 (q, ^3^*J*_C,F_ = 6.0 Hz, CH-12), 118.5 (q, ^3^*J*_C,F_ = 6.0 Hz, CH-18), 119.4 (CH-8), 124.2 (q, ^1^*J*_C,F_ = 274 Hz, CF_3_-11), 124.4 (q, ^1^*J*_C,F_ = 274 Hz, CF_3_-17), 124.9 (CH-14), 126.9 (C-10), 127.5 (q, ^1^*J*_C,F_ = 30 Hz, C-17), 127.8 (q, ^1^*J*_C,F_ = 30 Hz, C-11), 132.2 (C-16), 132.6 (CH-15 and C-3), 132.9 (CH-9), 135.0 (C-13), 140.1 (C-7), 143.34 (CH-21), 148.9 (C-19), 166.3 (CO-2).

^15^N NMR (DMSO-*d*_6_, 40.5 MHz, *δ* (ppm)): 85.7 (NH), 138.1 (N).

^19^F NMR (DMSO-*d*_6_, 376.5 MHz, *δ* (ppm)): −60.49, −60.48.

HRMS (MALDI-TOF/TOF) *m*/*z*: [M + H]^+^ calcd for C_24_H_19_F_6_N_2_O_2_ 481.13550; found 481.13550.

### 5-(4-Isopropylphenyl)-1-(4-methyl-3-(trifluoromethyl)phenyl)-3-((4-methyl-3-(trifluoromethyl)phenyl)amino)-1*H*-pyrrol-2(5*H*)-one (4k)

White solid; *η* = 73%, mp = 193–195 °C; IR(ATR) √(cm^−1^): 3730, 3705, 3504, 3319, 3024, 2970, 1740, 1687, 1651, 1622, 1544, 1504, 1427, 1365, 1218, 1121, 1050, 907, 828, 775, 672, 542, 454, 402.

^1^H NMR (DMSO-*d*_6_, 400.1 MHz, *δ* (ppm)): 1.12 (6H, d, ^3^*J* = 7 Hz, 2×CH_3_), 2.34 (6H, bs, 2×CH_3_), 2.81 (1H, septet, ^3^*J* = 7 Hz, CH), 6.10 (1H, d, ^3^*J* = 3 Hz, H-5), 6.39 (1H, d, ^3^*J* = 3 Hz, H-4), 7.17 (2H, d, ^3^*J*_H,H_ = 9 Hz, H-20), 7.20 (2H, d, ^3^*J*_H,H_ = 9 Hz, H-21), 7.28 (1H, d, ^3^*J* = 8 Hz, H-9), 7.38 (1H, d, ^3^*J* = 8 Hz, H-15), 7.43 (1H, dd, ^3^*J* = 8.3 Hz, ^4^*J* = 2 Hz, H-8), 7.65 (1H, d, ^4^*J* = 2 Hz, H-12), 7.68 (1H, dd, ^3^*J* = 8.3 Hz, ^4^*J* = 2 Hz, H-14), 8.11 (1H, d, ^4^*J* = 2 Hz, H-18), 8.47 (1H, s, NH).

^13^C NMR (DMSO-*d*_6_, 100.6 MHz, *δ* (ppm)): 17.8 (q, ^4^*J*_C,F_ = 1.5 Hz, CH_3_-10), 18.1 (q, ^4^*J*_C,F_ = 1.5 Hz, CH_3_-16), 23.6 (2×CH_3_), 32.9 (CH), 62.0 (CH-5), 111.0 (CH-4), 114.5 (q, ^3^*J*_C,F_ = 6.0 Hz, CH-12), 118.2 (q, ^3^*J*_C,F_ = 6.0 Hz, CH-18), 119.2 (CH-8), 124.1 (q, ^1^*J*_C,F_ = 274 Hz, CF_3_-11), 124.4 (q, ^1^*J*_C,F_ = 274 Hz, CF_3_-17), 124.5 (CH-14), 126.7 (CH-20 and CH-21), 126.6 (C-10), 127.5 (q, ^1^*J*_C,F_ = 30 Hz, C-17), 127.7 (q, ^1^*J*_C,F_ = 30 Hz, C-11), 131.4 (C-16), 131.6 (C-3), 132.6 (CH-15), 132.8 (CH-9), 134.5 (C-19), 135.2 (C-13), 140.2 (C-7), 148.1 (C-22), 166.55 (CO-2).

^15^N NMR (DMSO-*d*_6_, 40.5 MHz, *δ* (ppm)): 85.1 (NH), 141.6 (N).

^19^F NMR (DMSO-*d*_6_, 376.5 MHz, *δ* (ppm)): −60.50, −60.45.

HRMS (MALDI-TOF/TOF) *m*/*z*: [M + H]^+^ calcd for C_29_H_27_F_6_N_2_O 533.2027; found 533.2048.

### 5-(4-(*tert*-Butyl)phenyl)-1-(4-methyl-3-(trifluoromethyl)phenyl)-3-((4-methyl-3-(trifluoromethyl)phenyl)amino)-1*H*-pyrrol-2(5*H*)-one (4l)

White solid; *η* = 65%, mp = 203–204 °C; IR(ATR) √(cm^−1^): 3729, 3626, 3528, 3448, 3317, 2969, 1745, 1688, 1654, 1621, 1545, 1506, 1426, 1394, 1283, 1171, 1122, 1050, 908, 828, 776, 673, 647, 562, 457, 400.

^1^H NMR (DMSO-*d*_6_, 400.1 MHz, *δ* (ppm)): 1.20 (9H, s, 3×CH_3_), 2.34 (6H, bs, 2×CH_3_), 6.11 (1H, d, ^3^*J* = 3 Hz, H-5), 6.37 (1H, d, ^3^*J* = 3 Hz, H-4), 7.20 (2H, d, ^3^*J*_H,H_ = 9 Hz, H-20), 7.27 (1H, d, ^3^*J* = 8 Hz, H-9), 7.32 (2H, d, ^3^*J*_H,H_ = 9 Hz, H-21), 7.36 (1H, d, ^3^*J* = 8 Hz, H-15), 7.43 (1H, dd, ^3^*J* = 8.3 Hz, ^4^*J* = 2 Hz, H-8), 7.65 (1H, d, ^4^*J* = 2 Hz, H-12), 7.67 (1H, dd, ^3^*J* = 8.3 Hz, ^4^*J* = 2 Hz, H-14), 8.14 (1H, d, ^4^*J* = 2 Hz, H-18), 8.47 (1H, s, NH).

^13^C NMR (DMSO-*d*_6_, 100.6 MHz, *δ* (ppm)): 17.6 (q, ^4^*J*_C,F_ = 1.5 Hz, CH_3_-10), 17.8 (q, ^4^*J*_C,F_ = 1.5 Hz, CH_3_-16), 30.7 (3×CH_3_), 33.9 (C), 61.9 (CH-5), 110.9 (CH-4), 114.4 (q, ^3^*J*_C,F_ = 6.0 Hz, CH-12), 118.1 (q, ^3^*J*_C,F_ = 6.0 Hz, CH-18), 119.2 (CH-8), 124.0 (q, ^1^*J*_C,F_ = 274 Hz, CF_3_-11), 124.3 (q, ^1^*J*_C,F_ = 274 Hz, CF_3_-17), 124.2 (CH-14), 125.4 (CH-21), 126.3 (CH-20), 126.6 (C-10), 127.4 (q, ^1^*J*_C,F_ = 30 Hz, C-17), 127.7 (q, ^1^*J*_C,F_ = 30 Hz, C-11), 131.4 (C-3), 131.4 (C-16), 132.4 (CH-15), 132.7 (CH-9), 134.0 (C-19), 135.2 (C-13), 140.1 (C-7), 150.2 (C-22), 166.6 (CO-2).

^15^N NMR (DMSO-*d*_6_, 40.5 MHz, *δ* (ppm)): 85.1 (NH), 141.7 (N).

^19^F NMR (DMSO-*d*_6_, 376.5 MHz, *δ* (ppm)): −60.50, −60.48.

HRMS (MALDI-TOF/TOF) *m*/*z*: [M + H]^+^ calcd for C_30_H_29_F_6_N_2_O 547.2183; found 547.2167.

### 1-(4-Methyl-3-(trifluoromethyl)phenyl)-3-((4-methyl-3-(trifluoromethyl)phenyl)amino)-5-phenyl-1*H*-pyrrol-2(5*H*)-one (4m)

Yellow cristals; *η* = 62%, mp = 163–165 °C; IR(ATR) √(cm^−1^): 3319, 3027, 2970, 1740, 1677, 1648, 1536, 1505, 1404, 1319, 1281, 1217, 1163, 1106, 1050, 888, 827, 774, 695, 619, 531, 500, 452, 422.

^1^H NMR (DMSO-*d*_6_, 400.1 MHz, *δ* (ppm)): 2.35 (6H, bs, 2×CH_3_), 6.14 (1H, d, ^3^*J* = 3 Hz, H-5), 6.41 (1H, d, ^3^*J* = 3 Hz, H-4), 7.20–7.25 (1H, m, H-22), 7.27–7.33 (5H, m, H-9, H-20 and H-21), 7.37 (1H, d, ^3^*J* = 8 Hz, H-15), 7.44 (1H, dd, ^3^*J* = 8.3 Hz, ^4^*J* = 2 Hz, H-8), 7.65 (1H, d, ^4^*J* = 2 Hz, H-12), 7.70 (1H, dd, ^3^*J* = 8.3 Hz, ^4^*J* = 2 Hz, H-14), 8.08 (1H, d, ^4^*J* = 2 Hz, H-18), 8.49 (1H, s, NH).

^13^C NMR (DMSO-*d*_6_, 100.6 MHz, *δ* (ppm)): 17.9 (q, ^4^*J*_C,F_ = 1.5 Hz, CH_3_-10), 18.1 (q, ^4^*J*_C,F_ = 1.5 Hz, CH_3_-16), 62.3 (CH-5), 110.8 (CH-4), 114.5 (q, ^3^*J*_C,F_ = 6.0 Hz, CH-12), 118.2 (q, ^3^*J*_C,F_ = 6.0 Hz, CH-18), 119.3 (CH-8), 124.2 (q, ^1^*J*_C,F_ = 274 Hz, CF_3_-11), 124.4 (q, ^1^*J*_C,F_ = 274 Hz, CF_3_-17), 124.5 (CH-14), 126.7 (C-10), 126.9 (CH-20), 127.4 (q, ^1^*J*_C,F_ = 30 Hz, C-17), 127.8 (q, ^1^*J*_C,F_ = 30 Hz, C-11), 127.9 (CH-22), 128.8 (CH-21), 131.6 (C-3), 131.7 (C-16), 132.6 (CH-15), 132.9 (CH-9), 135.2 (C-13), 137.3 (C-19), 140.2 (C-7), 166.4 (CO-2).

^15^N NMR (DMSO-*d*_6_, 40.5 MHz, *δ* (ppm)): 85.1 (NH), 141.8 (N).

^19^F NMR (DMSO-*d*_6_, 376.5 MHz, *δ* (ppm)): −60.49, −60.54.

HRMS (MALDI-TOF/TOF) *m*/*z*: [M + H]^+^ calcd for C_26_H_21_F_6_N_2_O 491.1557; found 491.1545.

### 5-(4-Fluorophenyl)-1-(4-methyl-3-(trifluoromethyl)phenyl)-3-((4-methyl-3-(trifluoromethyl)phenyl)amino)-1*H*-pyrrol-2(5*H*)-one (4n)

Yellow cristals; *η* = 66%, mp = 169–170 °C; IR(ATR) √(cm^−1^): 3326, 3016, 2970, 1740, 1674, 1651, 1549, 1507, 1413, 1366, 1231, 1106, 1049, 887, 837, 778, 746, 661, 553, 535, 509, 451, 420.

^1^H NMR (DMSO-*d*_6_, 400.1 MHz, *δ* (ppm)): 2.34 (6H, bs, 2×CH_3_), 6.17 (1H, d, ^3^*J* = 3 Hz, H-5), 6.40 (1H, d, ^3^*J* = 3 Hz, H-4), 7.12 (2H, t, ^3^*J*_H,H_ = ^3^*J*_H,F_ = 9 Hz, H-21), 7.30 (1H, d, ^3^*J* = 8 Hz, H-9), 7.34 (2H, dd, ^3^*J*_H,H_ = 9 Hz, ^4^*J*_H,F_ = 5 Hz, H-20), 7.37 (1H, d, ^3^*J* = 8 Hz, H-15), 7.44 (1H, dd, ^3^*J* = 8.3 Hz, ^4^*J* = 2 Hz, H-8), 7.65 (1H, d, ^4^*J* = 2 Hz, H-12), 7.68 (1H, dd, ^3^*J* = 8.3 Hz, ^4^*J* = 2 Hz, H-14), 8.06 (1H, d, ^4^*J* = 2 Hz, H-18), 8.51 (1H, s, NH).

^13^C NMR (DMSO-*d*_6_, 100.6 MHz, *δ* (ppm)): 17.8 (q, ^4^*J*_C,F_ = 1.5 Hz, CH_3_-10), 18.1 (q, ^4^*J*_C,F_ = 1.5 Hz, CH_3_-16), 61.6 (CH-5), 110.6 (CH-4), 114.6 (q, ^3^*J*_C,F_ = 6.0 Hz, CH-12), 115.6 (d, ^2^*J*_C,F_ = 22 Hz, CH-21), 118.3 (q, ^3^*J*_C,F_ = 6.0 Hz, CH-18), 119.3 (CH-8), 124.2 (q, ^1^*J*_C,F_ = 274 Hz, CF_3_-11), 124.4 (q, ^1^*J*_C,F_ = 274 Hz, CF_3_-17), 124.6 (CH-14), 126.7 (C-10), 127.4 (q, ^1^*J*_C,F_ = 30 Hz, C-17), 127.8 (q, ^1^*J*_C,F_ = 30 Hz, C-11), 129.0 (d, ^3^*J*_C,F_ = 8 Hz, CH-20), 131.7 (C-3), 131.7 (C-16), 132.6 (CH-15), 132.9 (CH-9), 133.4 (d, ^4^*J*_C,F_ = 3 Hz, C-19), 135.1 (C-13), 140.2 (C-7), 161.6 (d, ^1^*J*_C,F_ = 244 Hz, C-22), 166.3 (CO-2).

^15^N NMR (DMSO-*d*_6_, 40.5 MHz, *δ* (ppm)): 84.8 (NH), 141.8 (N).

^19^F NMR (DMSO-*d*_6_, 376.5 MHz, *δ* (ppm)): −60.49, −60.54, −114.1.

HRMS (MALDI-TOF/TOF) *m*/*z*: [M + H]^+^ calcd for C_26_H_20_F_7_N_2_O 509.1463; found 509.1448.

### 5-(4-Chlorophenyl)-1-(4-methyl-3-(trifluoromethyl)phenyl)-3-((4-methyl-3-(trifluoromethyl)phenyl)amino)-1*H*-pyrrol-2(5*H*)-one (4o)

White crystals; *η* = 63%, mp = 171–172 °C; IR(ATR) √(cm^−1^): 3737, 3467, 3319, 3017, 2970, 2950, 1735, 1649, 1505, 1366, 1218, 1106, 1048, 912, 827, 777, 661, 528, 447, 413.

^1^H NMR (DMSO-*d*_6_, 400.1 MHz, *δ* (ppm)): 2.35 (6H, bs, 2×CH_3_), 6.18 (1H, d, ^3^*J* = 3 Hz, H-5), 6.40 (1H, d, ^3^*J* = 3 Hz, H-4), 7.29 (1H, d, ^3^*J* = 8 Hz, H-9), 7.32 (2H, d, ^3^*J*_H,H_ = 9 Hz, H-20), 7.36 (2H, d, ^3^*J*_H,H_ = 9 Hz, H-21), 7.37 (1H, d, ^3^*J* = 8 Hz, H-15), 7.44 (1H, dd, ^3^*J* = 8.3 Hz, ^4^*J* = 2 Hz, H-8), 7.65 (1H, d, ^4^*J* = 2 Hz, H-12), 7.67 (1H, dd, ^3^*J* = 8.3 Hz, ^4^*J* = 2 Hz, H-14), 8.09 (1H, d, ^4^*J* = 2 Hz, H-18), 8.53 (1H, s, NH).

^13^C NMR (DMSO-*d*_6_, 100.6 MHz, *δ* (ppm)): 17.8 (q, ^4^*J*_C,F_ = 1.5 Hz, CH_3_-10), 18.1 (q, ^4^*J*_C,F_ = 1.5 Hz, CH_3_-16), 61.6 (CH-5), 110.3 (CH-4), 114.6 (q, ^3^*J*_C,F_ = 6.0 Hz, CH-12), 118.2 (q, ^3^*J*_C,F_ = 6.0 Hz, CH-18), 119.4 (CH-8), 124.1 (q, ^1^*J*_C,F_ = 274 Hz, CF_3_-11), 124.4 (q, ^1^*J*_C,F_ = 274 Hz, CF_3_-17), 124.5 (CH-14), 126.8 (C-10), 127.5 (q, ^1^*J*_C,F_ = 30 Hz, C-17), 127.8 (q, ^1^*J*_C,F_ = 30 Hz, C-11), 128.8 (CH-20 and CH-21), 131.8 (C-3), 131.8 (C-16), 132.4 (CH-15), 132.6 (C-22), 132.9 (CH-9), 135.0 (C-13), 136.4 (C-19), 140.1 (C-7), 166.3 (CO-2).

^15^N NMR (DMSO-*d*_6_, 40.5 MHz, *δ* (ppm)): 85.3 (NH), 141.0 (N).

^19^F NMR (DMSO-*d*_6_, 376.5 MHz, *δ* (ppm)): −60.50, −60.54.

HRMS (MALDI-TOF/TOF) *m*/*z*: [M + H]^+^ calcd for C_26_H_20_ClF_6_N_2_O 525.1168; found 525.1149.

### 5-(4-Bromophenyl)-1-(4-methyl-3-(trifluoromethyl)phenyl)-3-((4-methyl-3-(trifluoromethyl)phenyl)amino)-1*H*-pyrrol-2(5*H*)-one (4p)

Yellow crystals; *η* = 61%, mp = 169–170 °C; IR(ATR) √(cm^−1^): 3729, 3321, 3015, 2970, 1735, 1647, 1507, 1366, 1282, 1218, 115, 1048, 1013, 889, 826, 777, 728, 663, 529, 450, 413.

^1^H NMR (DMSO-*d*_6_, 400.1 MHz, *δ* (ppm)): 2.34 (6H, bs, 2×CH_3_), 6.16 (1H, d, ^3^*J* = 3 Hz, H-5), 6.40 (1H, d, ^3^*J* = 3 Hz, H-4), 7.26 (2H, d, ^3^*J*_H,H_ = 9 Hz, H-20), 7.29 (1H, d, ^3^*J* = 8 Hz, H-9), 7.37 (1H, d, ^3^*J* = 8 Hz, H-15), 7.44 (1H, dd, ^3^*J* = 8.3 Hz, ^4^*J* = 2 Hz, H-8), 7.49 (2H, d, ^3^*J*_H,H_ = 9 Hz, H-21), 7.65 (1H, d, ^4^*J* = 2 Hz, H-12), 7.67 (1H, dd, ^3^*J* = 8.3 Hz, ^4^*J* = 2 Hz, H-14), 8.10 (1H, d, ^4^*J* = 2 Hz, H-18), 8.53 (1H, s, NH).

^13^C NMR (DMSO-*d*_6_, 100.6 MHz, *δ* (ppm)): 17.9 (q, ^4^*J*_C,F_ = 1.5 Hz, CH_3_-10), 18.0 (q, ^4^*J*_C,F_ = 1.5 Hz, CH_3_-16), 61.6 (CH-5), 110.2 (CH-4), 114.6 (q, ^3^*J*_C,F_ = 6.0 Hz, CH-12), 118.2 (q, ^3^*J*_C,F_ = 6.0 Hz, CH-18), 119.4 (CH-8), 121.0 (C-22), 124.1 (q, ^1^*J*_C,F_ = 274 Hz, CF_3_-11), 124.4 (q, ^1^*J*_C,F_ = 274 Hz, CF_3_-17), 124.5 (CH-14), 126.8 (C-10), 127.5 (q, ^1^*J*_C,F_ = 30 Hz, C-17), 127.8 (q, ^1^*J*_C,F_ = 30 Hz, C-11), 129.1 (CH-20), 131.7 (CH-21), 131.8 (C-3), 131.8 (C-16), 132.6 (CH-15), 132.9 (CH-9), 135.0 (C-13), 136.8 (C-19), 140.1 (C-7), 166.3 (CO-2).

^15^N NMR (DMSO-*d*_6_, 40.5 MHz, *δ* (ppm)): 85.2 (NH), 141.0 (N).

^19^F NMR (DMSO-*d*_6_, 376.5 MHz, *δ* (ppm)): −60.55, −60.52.

HRMS (MALDI-TOF/TOF) *m*/*z*: [M + H]^+^ calcd for C_26_H_20_BrF_6_N_2_O 569.0663; found 569.0687.

### 1-(4-Methyl-3-(trifluoromethyl)phenyl)-3-((4-methyl-3-(trifluoromethyl)phenyl)amino)-5-(4-(trifluoromethyl)phenyl)-1*H*-pyrrol-2(5*H*)-one (4r)

White crystals; *η* = 58%, mp = 162–163 °C; IR(ATR) √(cm^−1^): 3723, 3705, 3624, 3524, 3448, 3223, 2970, 1746, 1674, 1650, 1619, 1544, 1503, 1407, 1317, 1216, 1116, 1055, 1017, 888, 834, 778, 711, 659, 598, 528, 457, 419.

^1^H NMR (DMSO-*d*_6_, 400.1 MHz, *δ* (ppm)): 2.34 (6H, bs, 2×CH_3_), 6.29 (1H, d, ^3^*J* = 3 Hz, H-5), 6.44 (1H, d, ^3^*J* = 3 Hz, H-4), 7.29 (1H, d, ^3^*J* = 8 Hz, H-9), 7.38 (1H, d, ^3^*J* = 8 Hz, H-15), 7.44 (1H, dd, ^3^*J* = 8.3 Hz, ^4^*J* = 2 Hz, H-8), 7.53 (2H, d, ^3^*J* = 8 Hz, H-20), 7.65–7.69 (4H, m, H-14, H-12, H-21), 8.13 (1H, d, ^4^*J* = 2 Hz, H-18), 8.56 (1H, s, NH).

^13^C NMR (DMSO-*d*_6_, 100.6 MHz, *δ* (ppm)): 17.8 (q, ^4^*J*_C,F_ = 1.5 Hz, CH_3_-10), 18.0 (q, ^4^*J*_C,F_ = 1.5 Hz, CH_3_-16), 61.7 (CH-5), 109.9 (CH-4), 110.8 (C-22), 114.5 (q, ^3^*J*_C,F_ = 6.0 Hz, CH-12), 118.1 (q, ^3^*J*_C,F_ = 6.0 Hz, CH-18), 119.4 (CH-8), 123.9 (q, ^1^*J*_C,F_ = 274 Hz, CF_3_-22), 124.1 (q, ^1^*J*_C,F_ = 274 Hz, CF_3_-11), 124.4 (q, ^1^*J*_C,F_ = 274 Hz, CF_3_-17), 124.4 (CH-14), 125.7 (q, ^3^*J*_C,F_ = 6.0 Hz, CH-21), 126.8 (C-10), 127.6 (q, ^1^*J*_C,F_ = 30 Hz, C-17), 127.7 (CH-20), 127.8 (q, ^1^*J*_C,F_ = 30 Hz, C-11), 128.5 (q, ^1^*J*_C,F_ = 30 Hz, C-12), 131.9 (C-16), 132.0 (C-3), 132.7 (CH-15), 132.9 (CH-9), 135.0 (C-13), 140.1 (C-7), 142.3 (C-19), 166.4 (CO-2).

^15^N NMR (DMSO-*d*_6_, 40.5 MHz, *δ* (ppm)): 85.3 (NH), 140.8 (N).

^19^F NMR (DMSO-*d*_6_, 376.5 MHz, *δ* (ppm)): −61.12, −60.58, −60.52.

HRMS (MALDI-TOF/TOF) *m*/*z*: [M + H]^+^ calcd for C_27_H_20_F_9_N_2_O 559.1431; found 559.1439.

### 5-(3,5-Bis(trifluoromethyl)phenyl)-1-(4-methyl-3-(trifluoromethyl)phenyl)-3-((4-methyl-3-(trifluoromethyl)phenyl)amino)-1*H*-pyrrol-2(5*H*)-one (4s)

White crystals; *η* = 64%, mp = 145–147 °C; IR(ATR) √(cm^−1^): 3850, 3728, 3625, 3338, 3123, 2162, 1981, 1770, 1732, 1681, 1651, 1626, 1537, 1503, 1396, 1315, 1278, 1222, 1186, 1164, 1103, 1051, 896, 828, 776, 753, 732, 682, 649, 600, 507, 452, 419.

^1^H NMR (DMSO-*d*_6_, 400.1 MHz, *δ* (ppm)): 2.34 (6H, bs, 2×CH_3_), 6.42 (1H, d, ^3^*J* = 3 Hz, H-5), 6.53 (1H, d, ^3^*J* = 3 Hz, H-4), 7.29 (1H, d, ^3^*J* = 8 Hz, H-9), 7.39 (1H, d, ^3^*J* = 8 Hz, H-15), 7.46 (1H, dd, ^3^*J* = 8.3 Hz, ^4^*J* = 2 Hz, H-8), 7.67 (1H, d, ^3^*J* = 8 Hz, H-12), 7.74 (1H, dd, ^3^*J* = 8.3 Hz, ^4^*J* = 2 Hz, H-14), 7.97 (1H, bs, H-22), 8.03 (1H, d, ^4^*J* = 2 Hz, H-18), 8.05 (1H, bs, H-20, 24), 8.58 (1H, s, NH).

^13^C NMR (DMSO-*d*_6_, 100.6 MHz, *δ* (ppm)): 17.9 (q, ^4^*J*_C,F_ = 1.5 Hz, CH_3_-10), 18.0 (q, ^4^*J*_C,F_ = 1.5 Hz, CH_3_-16), 61.2 (CH-5), 109.1 (CH-4), 114.7 (q, ^3^*J*_C,F_ = 6.0 Hz, CH-12), 118.7 (q, ^3^*J*_C,F_ = 6.0 Hz, CH-18), 119.6 (CH-8), 121.9 (CH-22), 123.0 (q, ^1^*J*_C,F_ = 274 Hz, CF_3_-21, 23), 124.1 (q, ^1^*J*_C,F_ = 274 Hz, CF_3_-11), 124.4 (q, ^1^*J*_C,F_ = 274 Hz, CF_3_-17), 124.8 (CH-14), 127.0 (C-10), 127.6 (q, ^1^*J*_C,F_ = 30 Hz, C-17), 127.8 (q, ^1^*J*_C,F_ = 30 Hz, C-11), 128.2 (CH-20, 24), 130.6 (q, ^1^*J*_C,F_ = 30 Hz, C-21, 23), 132.2 (C-16), 132.5 (C-3), 132.8 (CH-15), 132.9 (CH-9), 134.7 (C-13), 140.1 (C-7), 141.2 (C-19), 166.3 (CO-2).

^15^N NMR (DMSO-*d*_6_, 40.5 MHz, *δ* (ppm)): 86.3 (NH), 140.6 (N).

^19^F NMR (DMSO-*d*_6_, 376.5 MHz, *δ* (ppm)): −61.40, −60.69, −60.52.

HRMS (MALDI-TOF/TOF) *m*/*z*: [M + H]^+^ calcd for C_28_H_19_F_12_N_2_O 627.1305; found 627.1305.

### 4-(1-(4-Methyl-3-(trifluoromethyl)phenyl)-4-((4-methyl-3-(trifluoromethyl)phenyl)amino)-5-oxo-2,5-dihydro-1*H*-pyrrol-2-yl)benzonitrile (4t)

White solid; *η* = 64%, mp = 180–182 °C; IR(ATR) √(cm^−1^): 3728, 3524, 3324, 2888, 1776, 1709, 1650, 1541, 1503, 1391, 1317, 1245, 1173, 1110, 1049, 827, 777, 707, 675, 559, 518, 447, 418.

^1^H NMR (DMSO-*d*_6_, 400.1 MHz, *δ* (ppm)): 2.35 (6H, bs, 2×CH_3_), 6.28 (1H, d, ^3^*J* = 3 Hz, H-5), 6.43 (1H, d, ^3^*J* = 3 Hz, H-4), 7.29 (1H, d, ^3^*J* = 8 Hz, H-9), 7.38 (1H, d, ^3^*J* = 8 Hz, H-15), 7.44 (1H, dd, ^3^*J* = 8.3 Hz, ^4^*J* = 2 Hz, H-8), 7.52 (2H, d, ^3^*J*_H,H_ = 9 Hz, H-20), 7.65 (1H, d, ^4^*J* = 2 Hz, H-12), 7.67 (1H, dd, ^3^*J* = 8.3 Hz, ^4^*J* = 2 Hz, H-14), 7.77 (2H, d, ^3^*J*_H,H_ = 9 Hz, H-21), 8.11 (1H, d, ^4^*J* = 2 Hz, H-18), 8.57 (1H, s, NH).

^13^C NMR (DMSO-*d*_6_, 100.6 MHz, *δ* (ppm)): 17.8 (q, ^4^*J*_C,F_ = 1.5 Hz, CH_3_-10), 18.1 (q, ^4^*J*_C,F_ = 1.5 Hz, CH_3_-16), 61.8 (CH-5), 109.6 (CH-4), 110.8 (C-22), 114.7 (q, ^3^*J*_C,F_ = 6.0 Hz, CH-12), 118.2 (q, ^3^*J*_C,F_ = 6.0 Hz, CH-18), 118.4 (CN), 119.4 (CH-8), 124.1 (q, ^1^*J*_C,F_ = 274 Hz, CF_3_-11), 124.4 (q, ^1^*J*_C,F_ = 274 Hz, CF_3_-17), 124.4 (CH-14), 126.9 (C-10), 127.6 (q, ^1^*J*_C,F_ = 30 Hz, C-17), 127.8 (q, ^1^*J*_C,F_ = 30 Hz, C-11), 127.9 (CH-20), 131.9 (C-16), 132.1 (C-3), 132.7 (CH-15), 132.8 (CH-21), 132.9 (CH-9), 134.9 (C-13), 140.1 (C-7), 143.3 (C-19), 166.4 (CO-2).

^15^N NMR (DMSO-*d*_6_, 40.5 MHz, *δ* (ppm)): 85.4 (NH), 140.8 (N).

^19^F NMR (DMSO-*d*_6_, 376.5 MHz, *δ* (ppm)): −60.55, −60.50.

HRMS (MALDI-TOF/TOF) *m*/*z*: [M + H]^+^ calcd for C_27_H_20_F_6_N_3_O 516.1510; found 516.1519.

### X-ray crystallography

Single-crystal X-ray diffraction data were collected on XtaLAB Synergy, Dualflex, HyPix diffractometer using Cu Kα radiation. The unit cell determination and data integration were carried out using the CrysAlisPro package from Oxford Diffraction.^45^ The multi-scan correction for absorption was applied. The structures were solved with SHELXT program using the intrinsic phasing method and refined by the full-matrix least-squares method on *F*^2^ with SHELXL.^46,47^ Olex2 was used as an interface to the SHELX programs.^48^ An anisotropic model was used for the refinement of non-hydrogen atoms. Hydrogen atoms were added in idealized positions and refined using a riding model. Selected crystallographic data and structure refinement details are provided in Table 1 and the corresponding CIF files. The supplementary crystallographic data can be obtained free of charge *via*https://www.ccdc.cam.ac.uk/conts/retrieving.html (or from the Cambridge Crystallographic Data Centre, 12 Union Road, Cambridge CB2 1EZ, UK; fax: (+44) 1223-336-033; or deposit@ccdc.ca.ac.uk).

### *In silico* ADMET predictions

The ADMET *in silico* evaluation for the compounds 4a–t was performed using the admetlab3 web tool (https://admetlab3.scbdd.com/server/evaluationCal) in terms of molecular properties, pharmacokinetics, drug-likeness, toxicity and medicinal chemistry.

### Antimicrobial activity

#### Microbial strains

The antimicrobial activity was tested against reference microbial strains, including Gram-positive bacteria (*Staphylococcus aureus* ATCC 25923), Gram-negative bacteria (*Pseudomonas aeruginosa* ATCC 27853), and yeast (*Candida albicans* ATCC 10231).

#### Quantitative evaluation of antimicrobial activity

Quantitative analysis was conducted in a 96-well plate using the serial binary microdilution method in liquid media—Tryptone Soy Broth for bacteria and Sabouraud Broth for yeast. Stock solutions were prepared at a concentration of 10 mg mL^−1^ in DMSO and then diluted to achieve final concentrations ranging from 5 to 0.016 mg mL^−1^. Simultaneously, serial dilutions using DMSO were used to create negative controls under the same conditions. Each well was inoculated with 10 µL of a microbial suspension standardized to 1.5 × 10^8^ CFU mL^−1^ from 18 to 24 h cultures. The minimum inhibitory concentration (MIC) was determined both visually, as the lowest concentration showing no visible microbial growth, and spectrophotometrically by measuring the absorbance of the cultures at 620 nm using a Molecular Devices FlexStation 3 UV-vis spectrophotometer (San Jose, CA, USA). A specific blank was prepared for each concentration. Data were analyzed using the Prism GraphPad 10.0 software's “log(inhibitory) *vs.* response—variable slope (four parameters)” function to calculate the IC_50_, representing the concentration required to inhibit 50% of microbial viability. For determining the minimal microbicidal concentration (MMC), 5 µL from each well was plated on solid media and incubated at 37 °C for 20–24 hours. The MMC was defined as the lowest concentration at which no microbial colonies were observed. The MMC/MIC ratio was calculated to differentiate between a microbicidal *vs.* microbiostatic effect.

#### The effect on microbial adherence

After quantitatively evaluating the antimicrobial activity, microbial adherence was analyzed using the slime assay. This involved methanol fixation and staining with 0.1% crystal violet. The absorbance of the biological material, suspended in 33% acetic acid, was then measured at 490 nm.

### Hemocompatibility

The hemolytic index of the derivatives was assessed using a spectrophotometric method, as previously described.^49^ Nine milliliters of ram blood were collected in ACD tubes and centrifuged at 2000 rpm for 10 minutes. The supernatant was discarded, and the remaining pellet was washed three times with PBS (0.2 M, pH 7.4) before being resuspended in sterile 0.9% saline solution. Next, 100 µL of each sample (2.5 mg mL^−1^ in DMSO) was added to 400 µL of a 10% erythrocyte suspension. The mixtures were incubated for 1 hour at 37 °C and then centrifuged at 2000 rpm for 10 minutes. Absorbance of the supernatant was measured at 540 nm. Controls were prepared to represent 100% hemolysis (*A*+) using 1% Triton X-100 and 0% hemolysis (*A*−) using 0.9% saline solution. A solvent control was also included. All experiments were performed in triplicate, and hemolytic activity was calculated as a percentage of hemolysis inhibition using the following formula:Hemolysis (%) = (*A*_sample_ − *A*−) × 100/(*A*+ − *A*−)

The ram blood used for hemocompatibility tests was purchased from an accredited commercial biobank, SC Sanimed International Impex SRL. The blood was collected by the supplier in accordance with institutional animal welfare regulations, from donor animals maintained exclusively for non-lethal blood collection, without requiring the sacrifice of animals for this study. Since the study did not involve experimental procedures on live animals, obtaining ethical approval was not necessary, in accordance with Directive 2010/63/EU.

### Biocompatibility

The biocompatibility of the compounds was evaluated using HaCaT human keratinocytes (sourced from Cell Line Services (CLS), Eppelheim, Germany, product number: 300493, RRID: CVCL_0038). The cells were cultured in DMEM (Dulbecco's Modified Eagle Medium) supplemented with 10% fetal bovine serum and 1% penicillin/streptomycin (50 µg mL^−1^) (all sourced from Sigma-Aldrich, St. Louis, MO) for 24 hours at 37 °C in a humidified atmosphere of 95% humidity and 5% CO_2_. Following incubation, the cells were washed with saline (Sigma-Aldrich, St. Louis, MO), trypsinized using 0.25% trypsin-EDTA (Thermo Fisher Scientific, Waltham, MA), and counted with a hemocytometer after staining with trypan blue. The compounds were then co-cultured with the cells at a density of 5 × 10^5^ cells per well for another 24 hours under the same conditions (37 °C, 95% humidity, 5% CO_2_). Control cells were maintained in complete cell culture medium.

Cell viability in the presence of the compounds was evaluated using the MTT assay (Vybrant® MTT Cell Proliferation Assay Kit, cat. no. V-13154, Thermo Fisher Scientific, Waltham, MA). The cells were incubated with MTT reagent at 37 °C in a humidified environment of 95% humidity and 5% CO_2_. After incubation, isopropanol was added to solubilize the formazan crystals, and the optical densities were measured at 550 nm using a Multiskan FC UV-vis spectrophotometer (Thermo Fisher Scientific, Waltham, MA).

### Statistical analysis

The data are expressed as mean values ± standard deviation (SD), based on triplicate analysis. Statistical analysis was performed using GraphPad Prism version 10. A two-way ANOVA was employed to compare the IC_50_ and cytotoxicity of derivatives *vs.* general structure and solvent used, followed by Tukey's *post hoc* test for multiple comparisons. Hemocompatibility was analyzed by one-way ANOVA using Dunnett's multiple comparisons test, with a single pooled variance method. Statistical significance was defined as *p* < 0.05.

## Author contributions

The manuscript was written through the contributions of all authors. All authors have approved the final version of the manuscript. Synthesis was conducted by C. M. A.-M., and R. P. V.; physical–chemical characterization was made by C. M. A.-M., A. N., M. S., M. P., and S. S.; cell-based biological assays were conducted by I. C. M., M. D. G., M. S. and M. C. C.; conceptualization was made by C. M. A.-M., M. C. C. and M. P., funding was acquired by M. C. C., M. P. and C. M. A.-M.

## Conflicts of interest

There are no conflicts to declare.

## Supplementary Material

RA-016-D6RA05763B-s001

## Data Availability

CCDC 2372608–2372615 contain the supplementary crystallographic data for this paper.^50*a*–*h*^ The data supporting the findings of this study are available within the article and its supplementary information (SI). Supplementary information is available. See DOI: https://doi.org/10.1039/d6ra05763b.
