# Supplementary material for: Heterocyclic chemistry meets wound care: novel p-methyl-m-trifluoromethyl-phenyl-substituted pyrrol-2-one derivatives with antimicrobial and antibiofilm activity
Source: RSC Adv. 2026 Aug 7;16(40):46074–93. doi: 10.1039/d6ra05763b (PMC13448452; doi:10.1039/d6ra05763b)
Supplement: Supplementary file 1 [file RA-016-D6RA05763B-s001.pdf]

Supplementary Information (SI) for RSC Advances.
This journal is © The Royal Society of Chemistry 2026

Supplementary material for

Heterocyclic Chemistry Meets Wound Care: Novel *p*-Methyl-*m*-Trifluoromethyl-Phenyl-Substituted Pyrrol-2-one Derivatives with Antimicrobial and Antibiofilm Activity

Ioana C. Marinas[1,§], Alina Nicolescu[2,§], Cristina M. Al-Matarneh[1,3*], Rafaela Vrabie[3,4], Sergiu Shova[5], Mihaela Silion[6], Mădălina-Diana Gaboreanu[1,7], Miruna Stan[1,8], Mariana C. Chifiriuc[1,7,9], Mariana Pinteala[3].

[1]*Research Institute of the University of Bucharest-ICUB, 91-95 Spl. Independentei, 050095 Bucharest, Romania*

[2]*NMR Laboratory "Petru Poni" Institute of Macromolecular Chemistry of Romanian Academy, 41A Grigore Ghica Voda Alley, Iasi 700487, Romania*

[3]*Centre of Advanced Research in Bionanoconjugates and Biopolymers, "Petru Poni" Institute of Macromolecular Chemistry of Romanian Academy, 41A Grigore Ghica Voda Alley, Iasi 700487, Romania*

[4]*"Cristofor Simionescu" Faculty of Chemical Engineering and Environmental Protection "Gheorghe Asachi" Technical University of Iasi, 73 D. Mangeron Blvd., 700050 Iasi, Romania.*

[5]*Department of Inorganic Polymers "Petru Poni" Institute of Macromolecular Chemistry of Romanian Academy, 41A Grigore Ghica Voda Alley, Iasi 700487, Romania*

[6]*Physics of Polymers and Polymeric Materials Department, "Petru Poni" Institute of Macromolecular Chemistry, 41A Grigore Ghica Voda Alley, 700487, Iasi, Romania;*

[7]*Department of Botany and Microbiology, Faculty of Biology, Splaiul Indeopenentei 91-95, Bucharest R- 050095, Romania*

[8]*Faculty of Biology, Department of Biochemistry and Molecular Biology, University of Bucharest, 91-95 Splaiul Independentei, 050095, Bucharest, Romania*

[9]*The Romanian Academy, 25, Calea Victoriei, Sector 1, District 1, Bucharest 010071, Romania*

§ authors with equal contributions

[*] almatarneh.cristina@icmpp.ro;

Contents

Figure 1S. 1H-NMR spectrum **4b** recorded in DMSO-d6, at 400.1 MHz. 4

Figure 2S. ^{13}C-NMR spectrum **4b** recorded in DMSO-d_6, at 100.6 MHz. 5

Figure 3S. ^{19}F-NMR spectrum **4b** recorded in DMSO-d_6, at 376.4 MHz. 5

Figure 4S. H,N-HMBC spectrum **4b** recorded in DMSO-d_6, at 40.5 MHz. 6

Figure 6S. FT-IR spectra for compound **4b** 7

Figure 7S. ^{1}H-NMR spectrum **4l** recorded in DMSO-d_6, at 400.1 MHz. 8

Figure 8S. ^{13}C-NMR spectrum **4l** recorded in DMSO-d_6, at 100.6 MHz. 8

Figure 9S. ^{19}F-NMR spectrum **4l** recorded in DMSO-d_6, at 376.4 MHz. 9

Figure 10S. H,N-HMBC spectrum **4l** recorded in DMSO-d_6, at 40.5 MHz. 9

Figure 11S. MALDI-MS spectrum for **4l** 10

Figure 12S. FT-IR spectra for compound **4l** 10

Figure 13S. ^{1}H-NMR spectrum **4o** recorded in DMSO-d_6, at 400.1 MHz. 11

Figure 14S. ^{13}C-NMR spectrum **4o** recorded in DMSO-d_6, at 100.6 MHz. 11

Figure 15S. ^{19}F-NMR spectrum **4o** recorded in DMSO-d_6, at 376.4 MHz. 12

Figure 16S. H,N-HMBC spectrum **4o** recorded in DMSO-d_6, at 40.5 MHz. 12

Figure 17S. MALDI-MS spectrum for **4o** 13

Figure 18S. FT-IR spectra for compound **4o** 13

Figure 19S. ^{1}H-NMR spectrum **4t** recorded in DMSO-d_6, at 400.1 MHz. 14

Figure 20S. ^{13}C-NMR spectrum **4t** recorded in DMSO-d_6, at 100.6 MHz. 14

Figure 21S. ^{19}F-NMR spectrum **4t** recorded in DMSO-d_6, at 376.4 MHz. 15

Figure 22S. H,N-HMBC spectrum **4t** recorded in DMSO-d_6, at 40.5 MHz. 15

Figure 23S. MALDI-MS spectrum for **4t** 16

Figure 24S. FT-IR spectra for compound **4t** 16

Figure S25. HPLC spectra for compound **4d** (280nm) 17

Figure S26. HPLC spectra for compound **4k** (280nm) 17

Figure S27. HPLC spectra for compound **4l** (280nm) 18

Figure S28. HPLC spectra for compound **4n** (280nm) 18

Figure S29. HPLC spectra for compound **4r** (280nm) 19

Figure S30. HPLC spectra for compound **4s** (280nm) 19

Table 1S. Radar diagram for compounds 20

Table S2. Physicochemical Properties 25
Table S3. Hydrogen Bonding and Molecular Flexibility........ 26
Table S4. Absorption and Permeability 26
Table S5. Transporter Interaction........ 27
Table S6. Skin and Ocular Safety Predictions 27
Table S7. Toxicological Alerts........ 28
Table S8. Medicinal Chemistry Descriptors 28
Table S9. Drug-likeness and Developability........ 29
Table S10. Experimental Reliability Risks 29
Table S11. Antimicrobial-Relevant Summary 30
Figure 31S. ^{1}H-NMR spectrum **4a** recorded in DMSO-d6, at 400.1 MHz........ 31
Figure 32S. ^{13}C-NMR spectrum **4a** recorded in DMSO-d_6, at 100.6 MHz. 31
Figure 33S. ^{1}H-NMR spectrum **4c** recorded in DMSO-d6, at 400.1 MHz........ 32
Figure 34S. ^{13}C-NMR spectrum **4c** recorded in DMSO-d_6, at 100.6 MHz........ 32
Figure 35S. ^{1}H-NMR spectrum **4d** recorded in DMSO-d6, at 400.1 MHz. 33
Figure 36S. ^{13}C-NMR spectrum **4d** recorded in DMSO-d_6, at 100.6 MHz. 33
Figure 37S. ^{1}H-NMR spectrum **4e** recorded in DMSO-d6, at 400.1 MHz........ 34
Figure 38S. ^{13}C-NMR spectrum **4e** recorded in DMSO-d_6, at 100.6 MHz........ 34
Figure 39S. ^{1}H-NMR spectrum **4f** recorded in DMSO-d6, at 400.1 MHz. 35
Figure 40S. ^{13}C-NMR spectrum **4f** recorded in DMSO-d_6, at 100.6 MHz. 35
Figure 41S. ^{1}H-NMR spectrum **4g** recorded in DMSO-d6, at 400.1 MHz........ 36
Figure 42S. ^{13}C-NMR spectrum **4g** recorded in DMSO-d_6, at 100.6 MHz. 36
Figure 44S. ^{13}C-NMR spectrum **4h** recorded in DMSO-d_6, at 100.6 MHz. 37
Figure 46S. ^{13}C-NMR spectrum **4i** recorded in DMSO-d_6, at 100.6 MHz. 38
Figure 48S. ^{13}C-NMR spectrum **4j** recorded in DMSO-d_6, at 100.6 MHz. 39
Figure 50S. ^{13}C-NMR spectrum **4k** recorded in DMSO-d_6, at 100.6 MHz. 40
Figure 52S. ^{13}C-NMR spectrum **4m** recorded in DMSO-d_6, at 100.6 MHz. 41
Figure 54S. ^{13}C-NMR spectrum **4n** recorded in DMSO-d_6, at 100.6 MHz. 42
Figure 56S. ^{13}C-NMR spectrum **4p** recorded in DMSO-d_6, at 100.6 MHz. 43

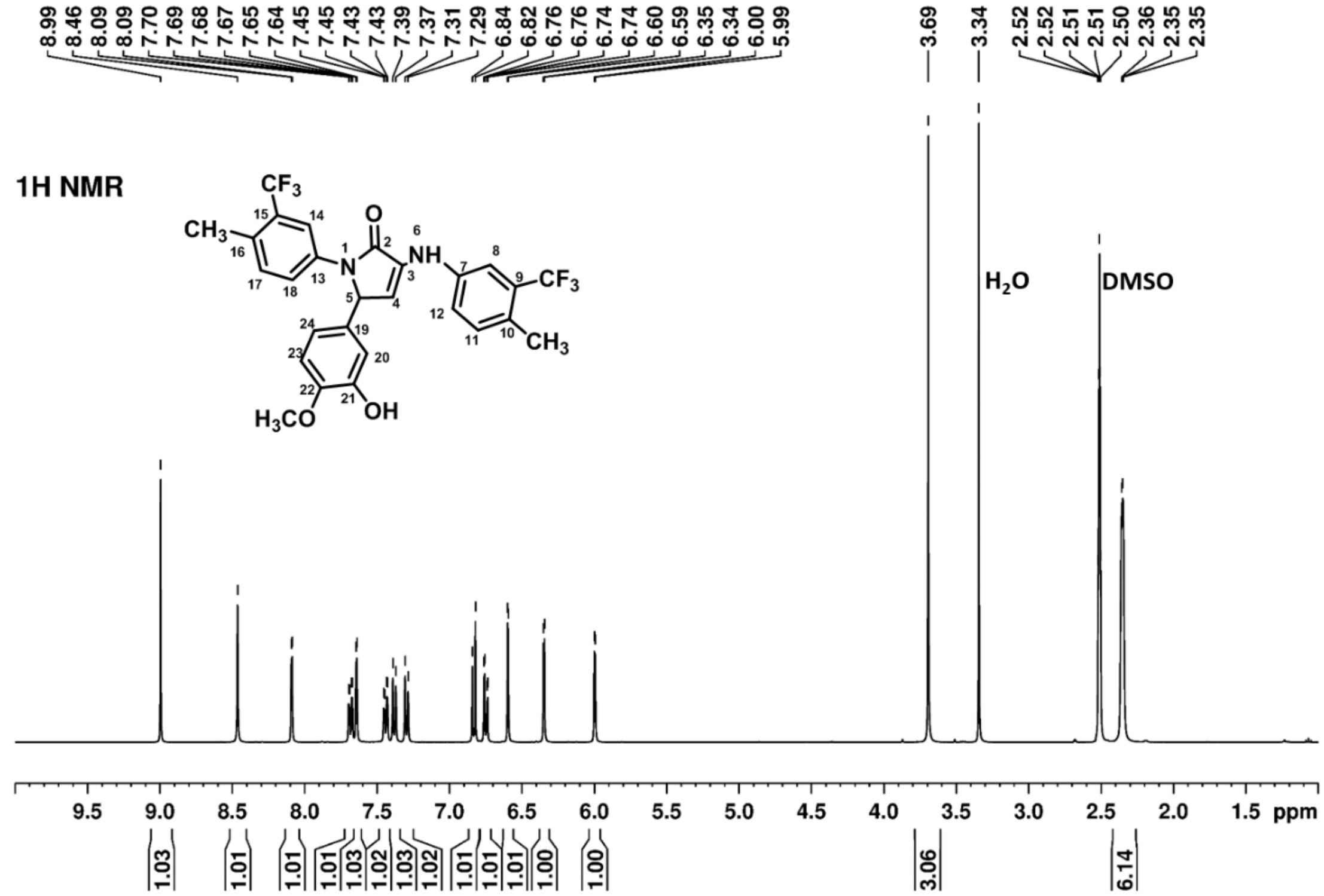

Figure 1S. 1H-NMR spectrum **4b** recorded in DMSO-d6, at 400.1 MHz.

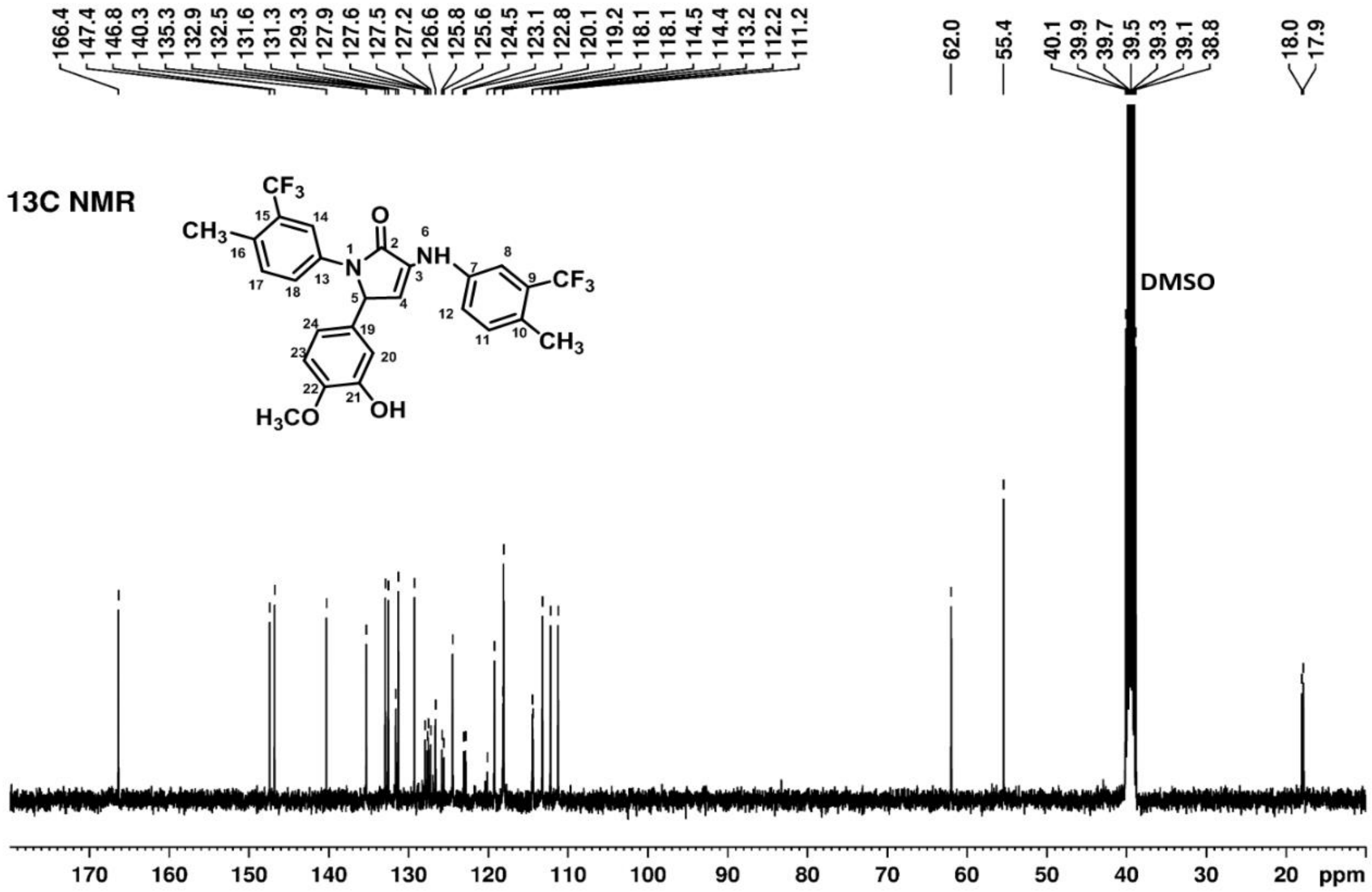

Figure 2S. ^{13}C-NMR spectrum **4b** recorded in DMSO-d$_6$, at 100.6 MHz.

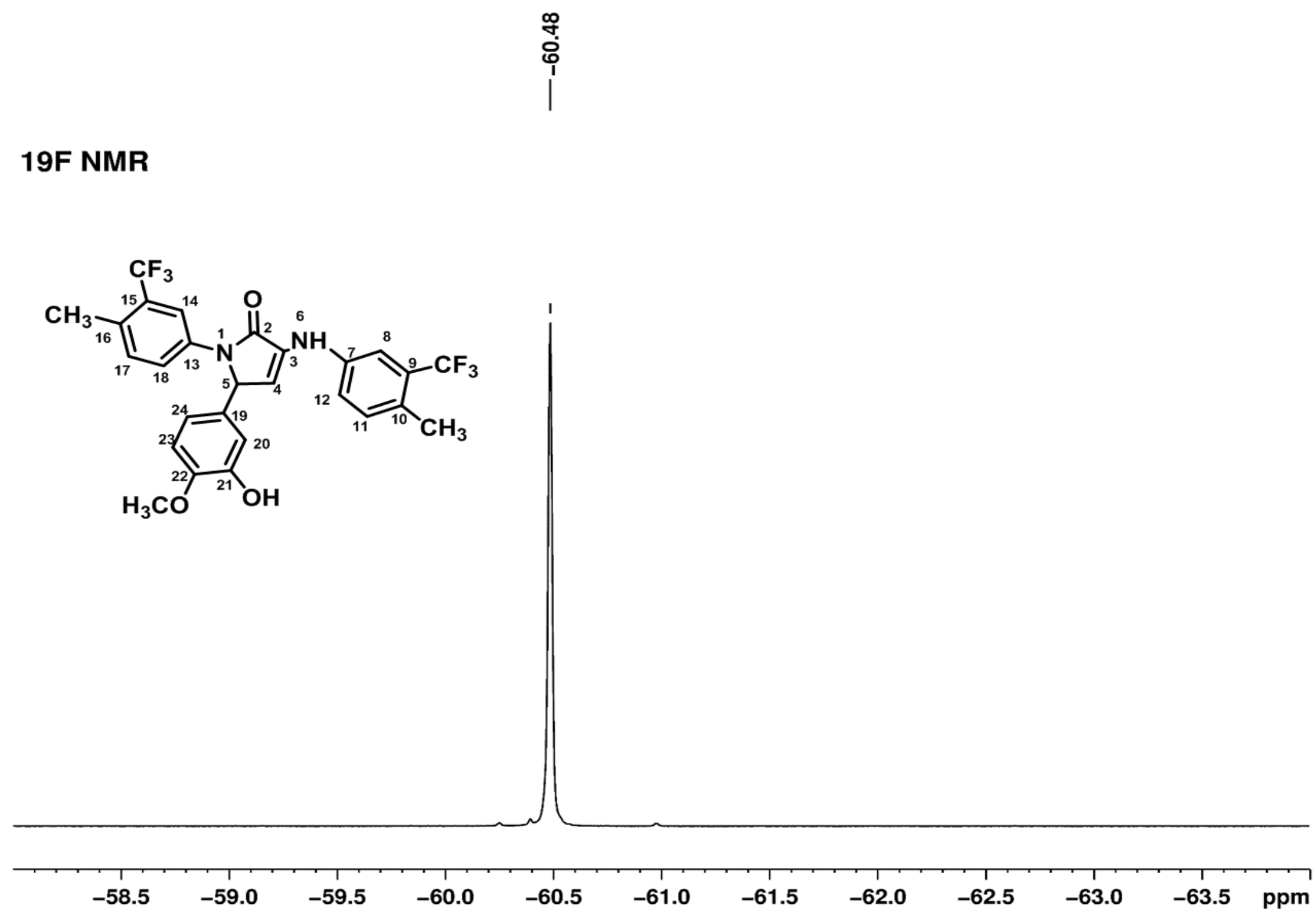

Figure 3S. ^{19}F-NMR spectrum **4b** recorded in DMSO-d$_6$, at 376.4 MHz.

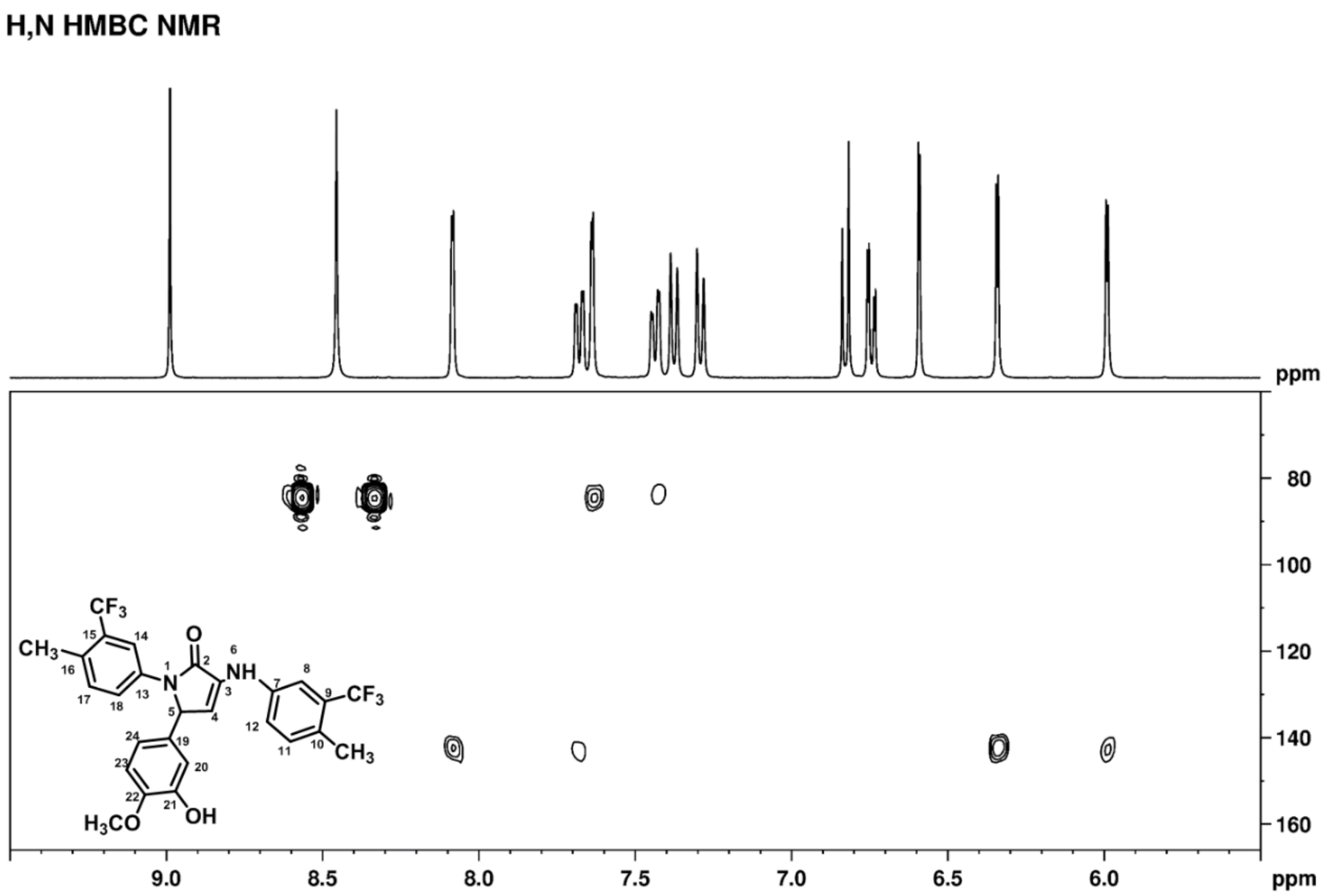

Figure 4S. H,N-HMBC spectrum **4b** recorded in DMSO-d$_6$, at 40.5 MHz.

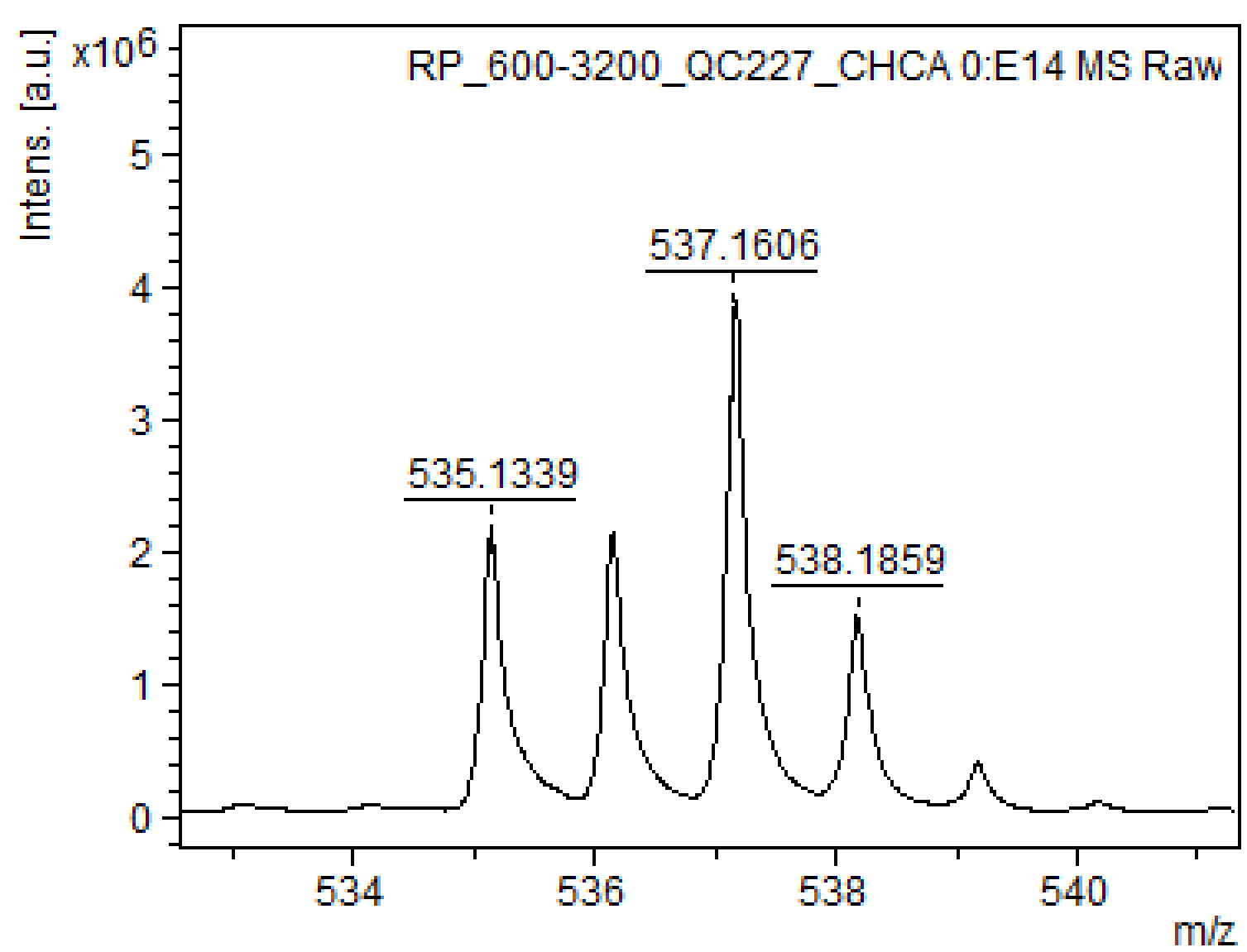

Figure 5S. MALDI-MS spectrum for **4b**

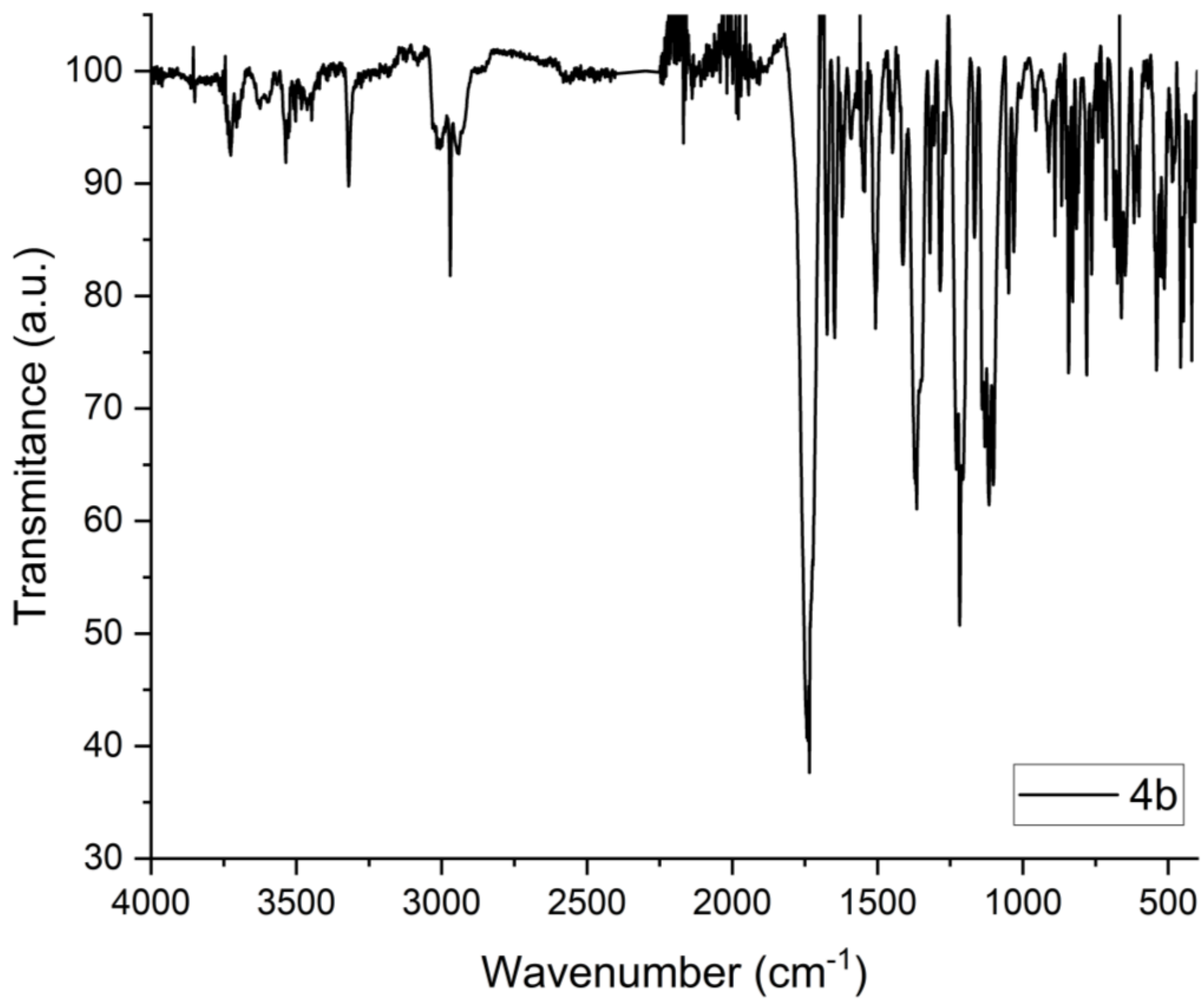

Figure 6S. FT-IR spectra for compound **4b**

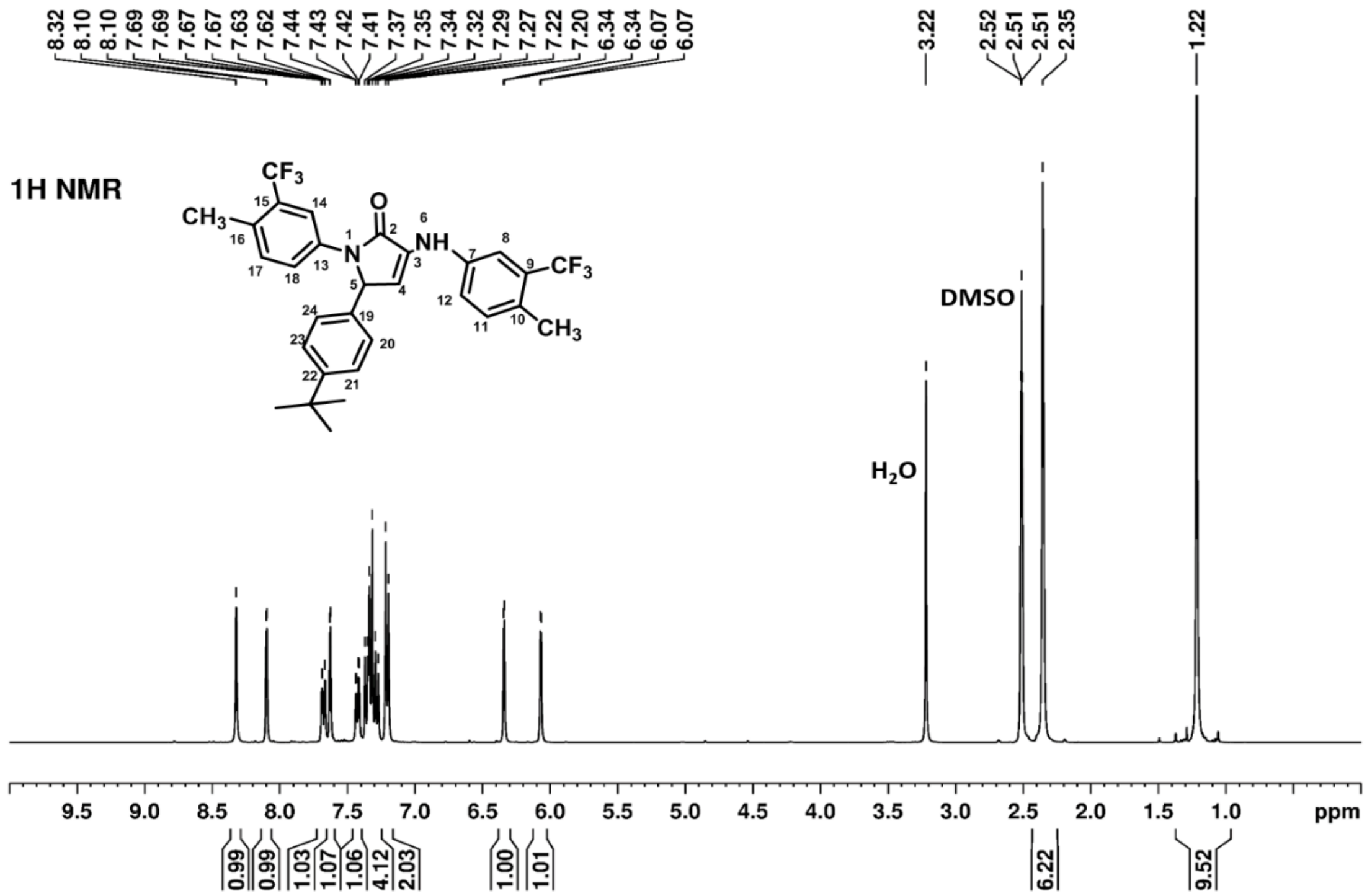

Figure 7S. ^{1}H-NMR spectrum **4l** recorded in DMSO-d$_6$, at 400.1 MHz.

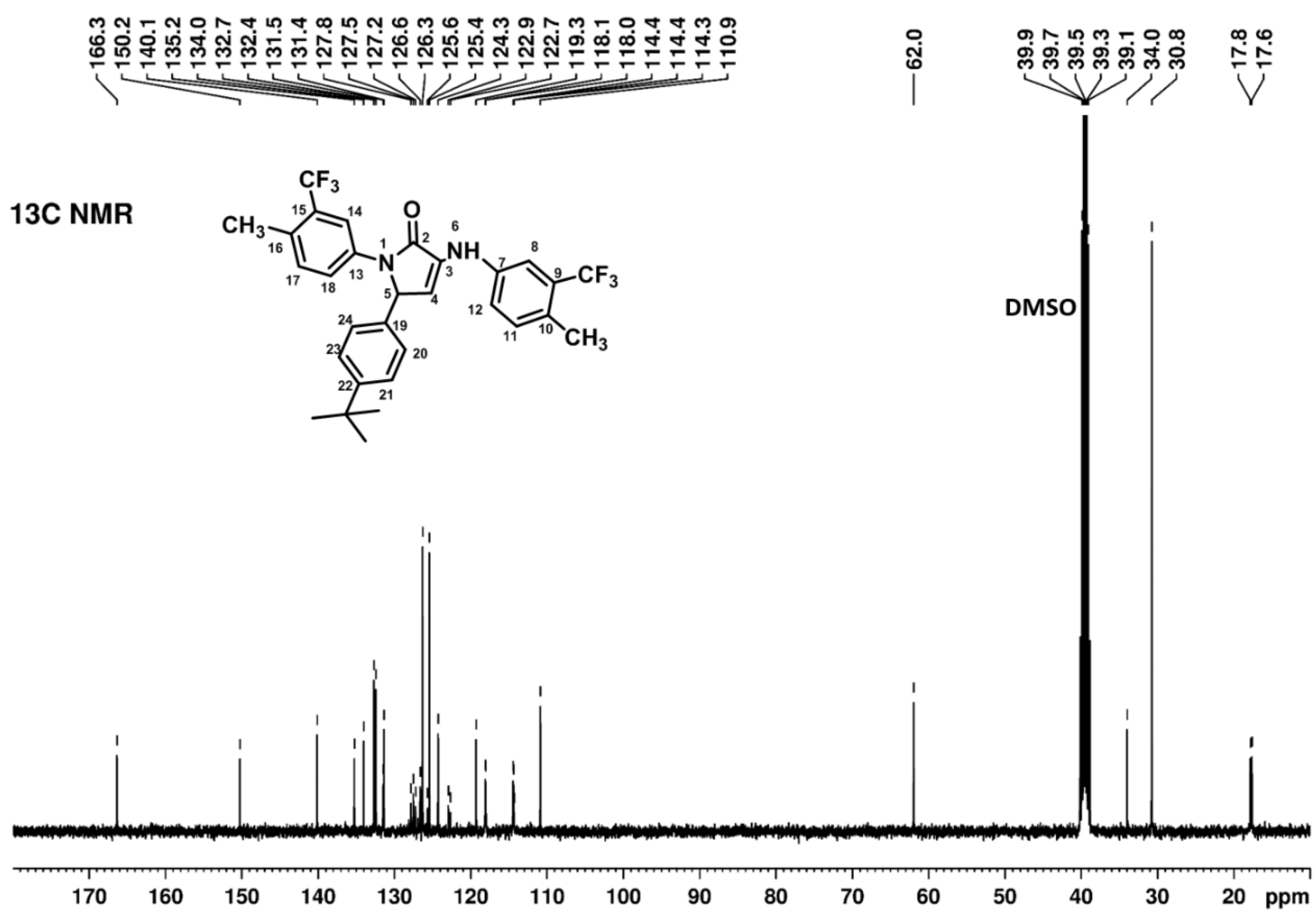

Figure 8S. ^{13}C-NMR spectrum **4l** recorded in DMSO-d$_6$, at 100.6 MHz.

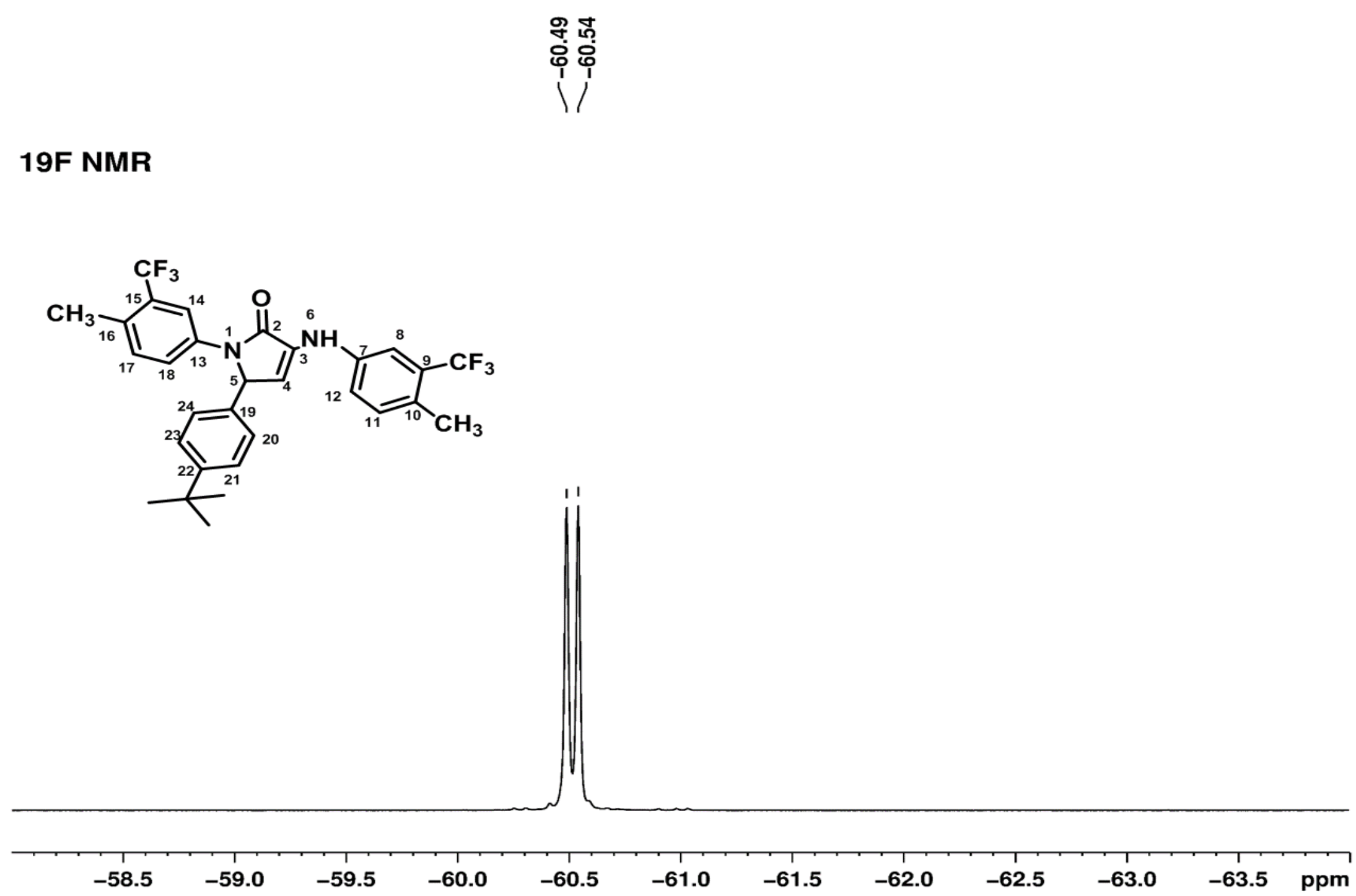

Figure 9S. ^{19}F-NMR spectrum **4l** recorded in DMSO-d$_6$, at 376.4 MHz.

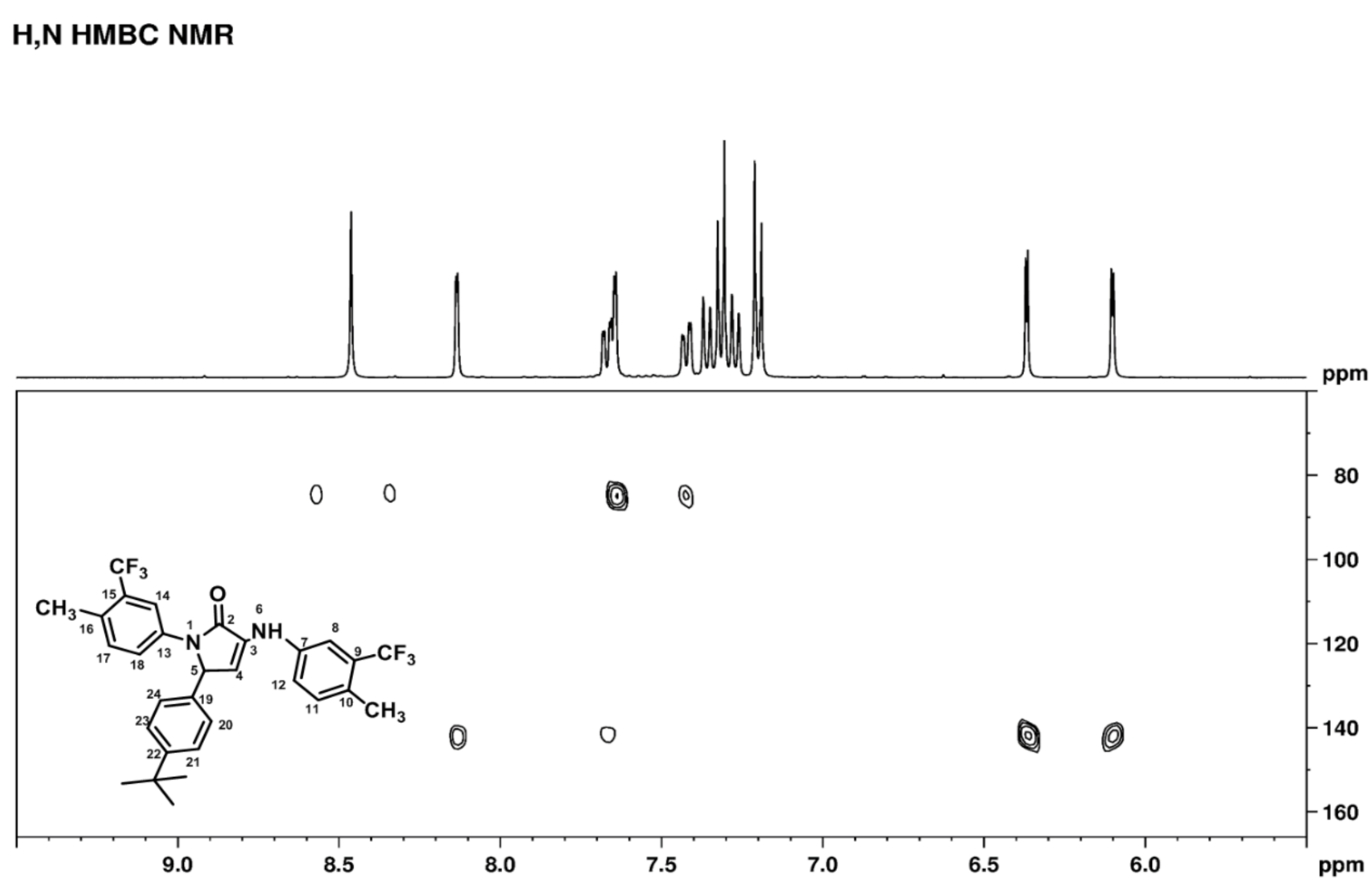

Figure 10S. H,N-HMBC spectrum **4l** recorded in DMSO-d$_6$, at 40.5 MHz.

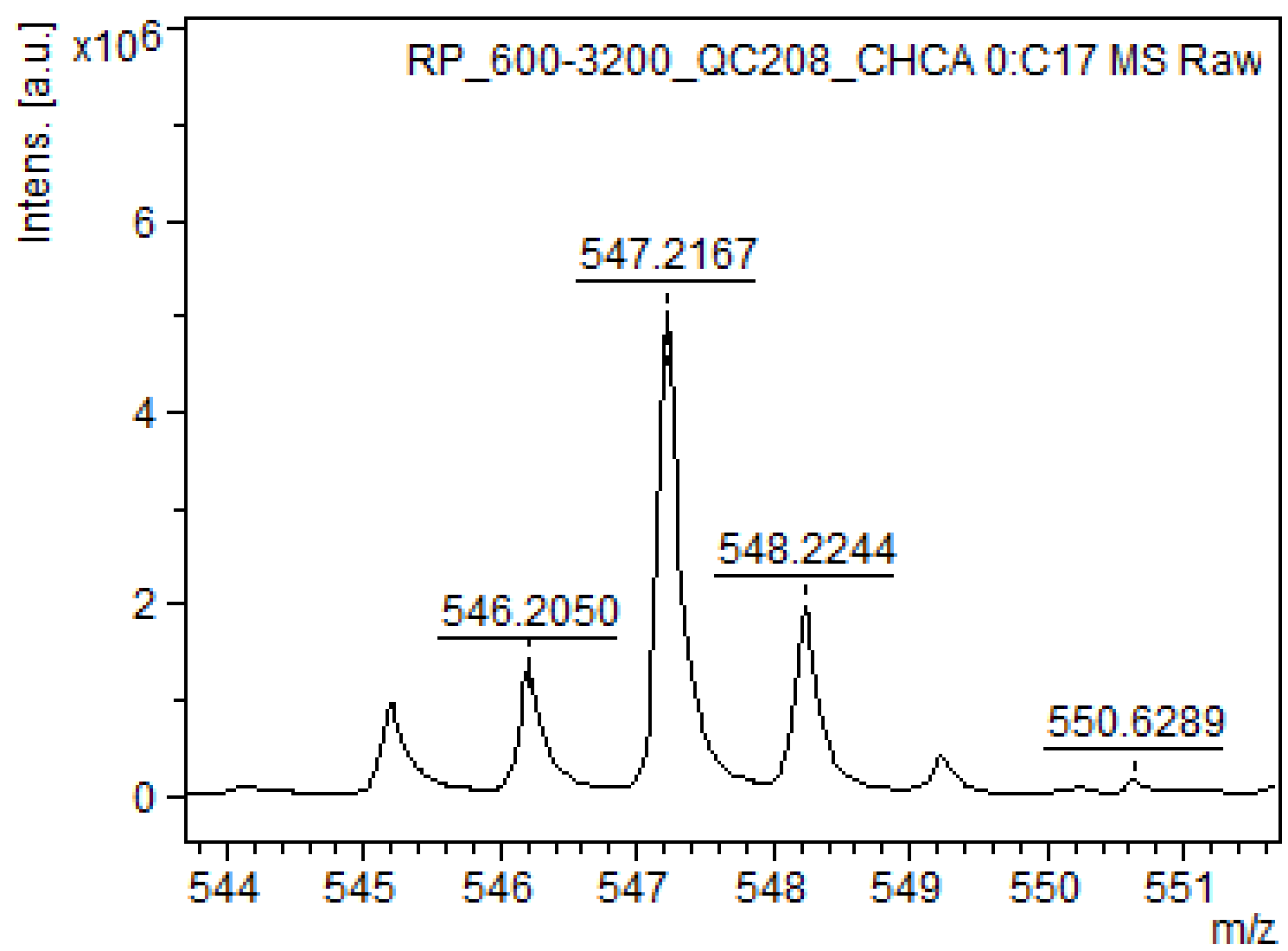

Figure 11S. MALDI-MS spectrum for **4l**

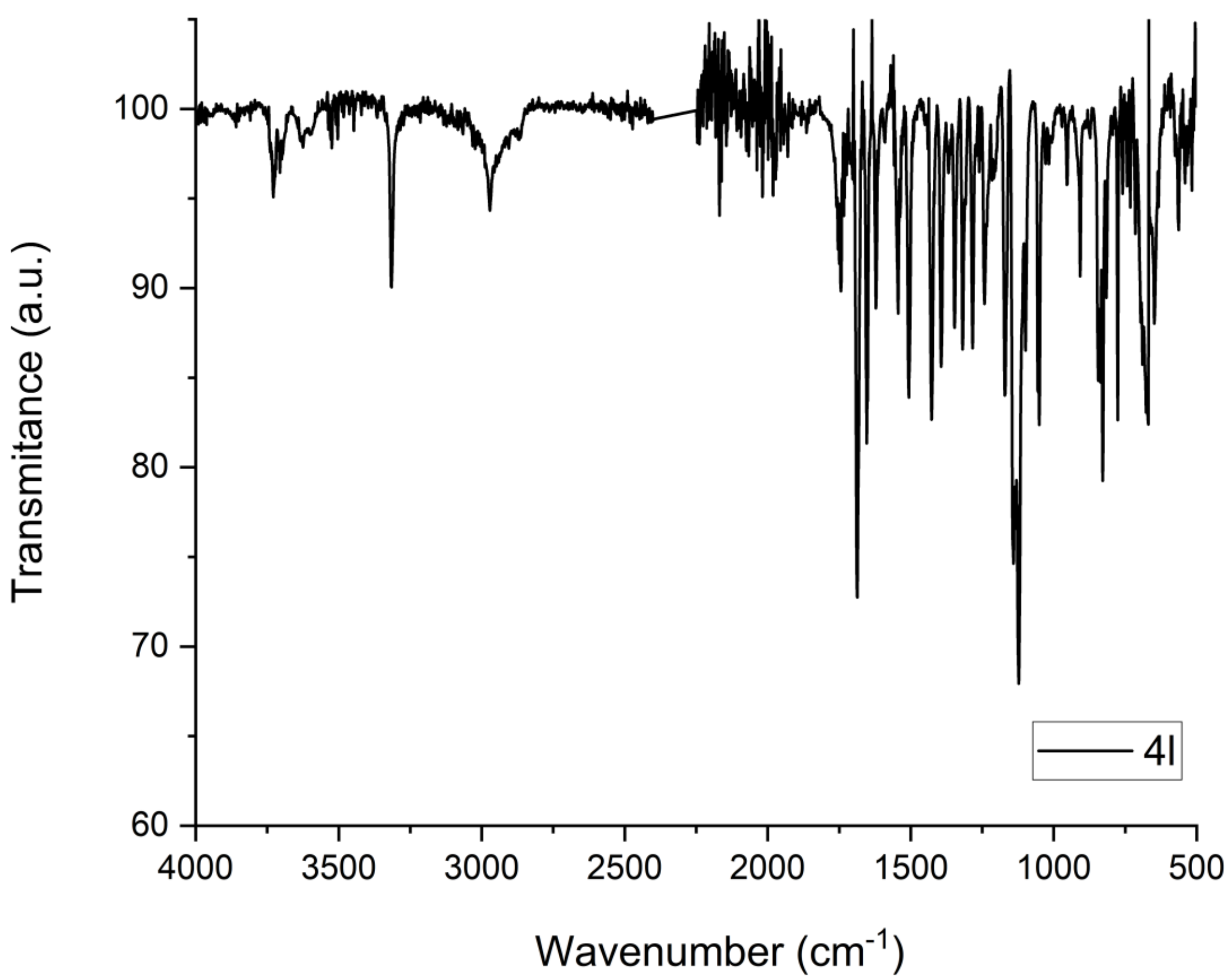

Figure 12S. FT-IR spectra for compound **4l**

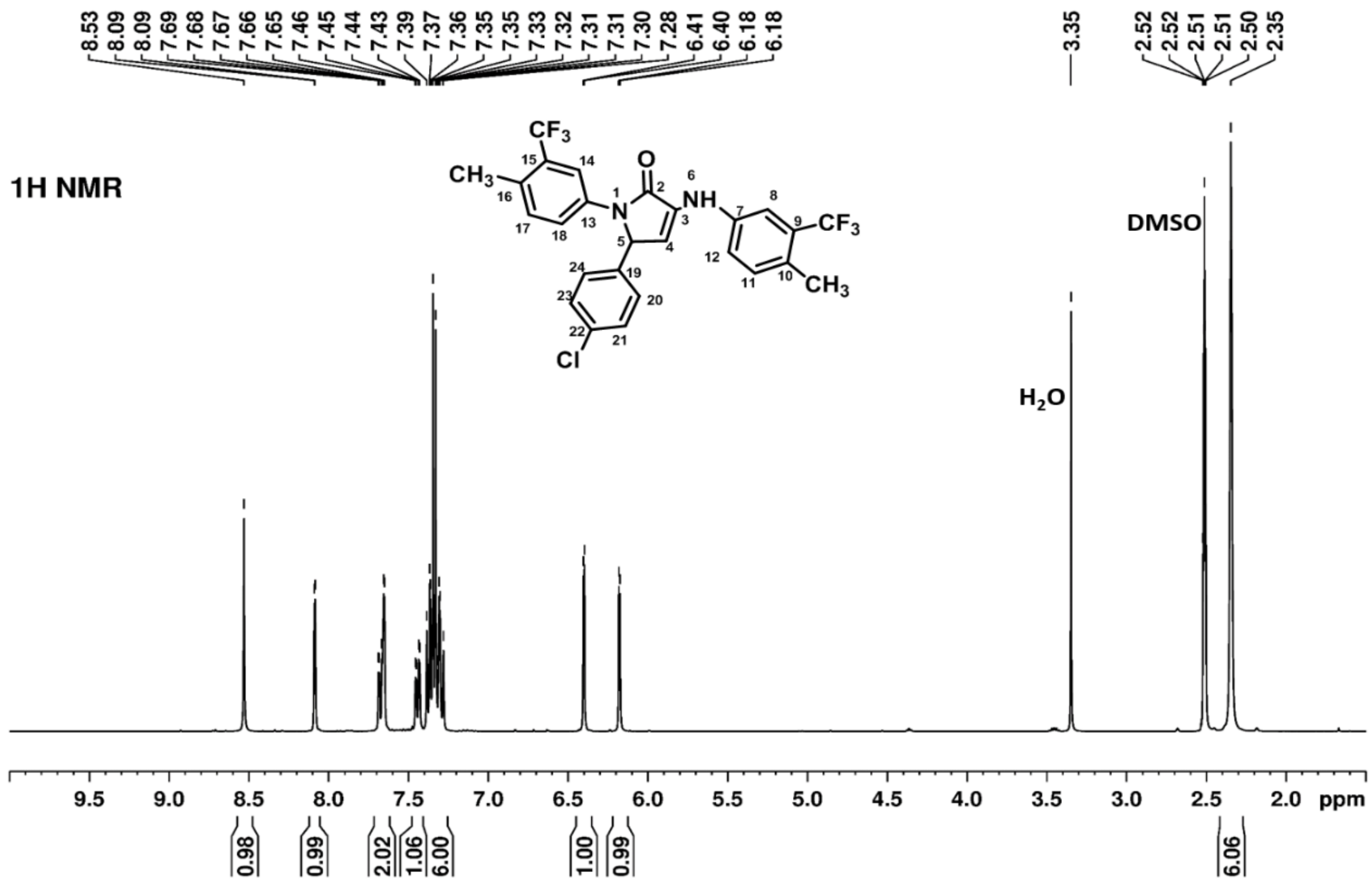

Figure 13S. ^{1}H-NMR spectrum **4o** recorded in DMSO-d$_6$, at 400.1 MHz.

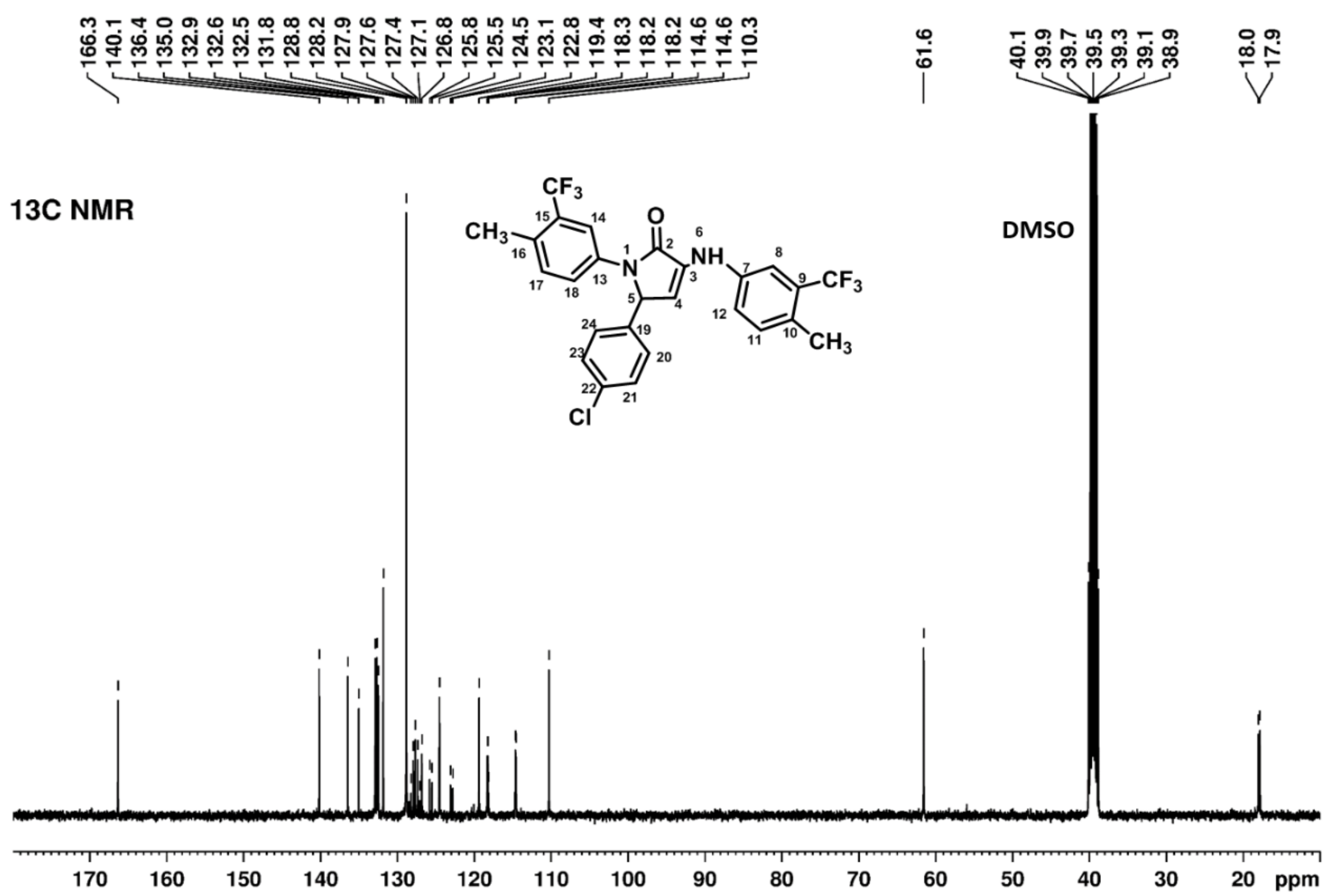

Figure 14S. ^{13}C-NMR spectrum **4o** recorded in DMSO-d$_6$, at 100.6 MHz.

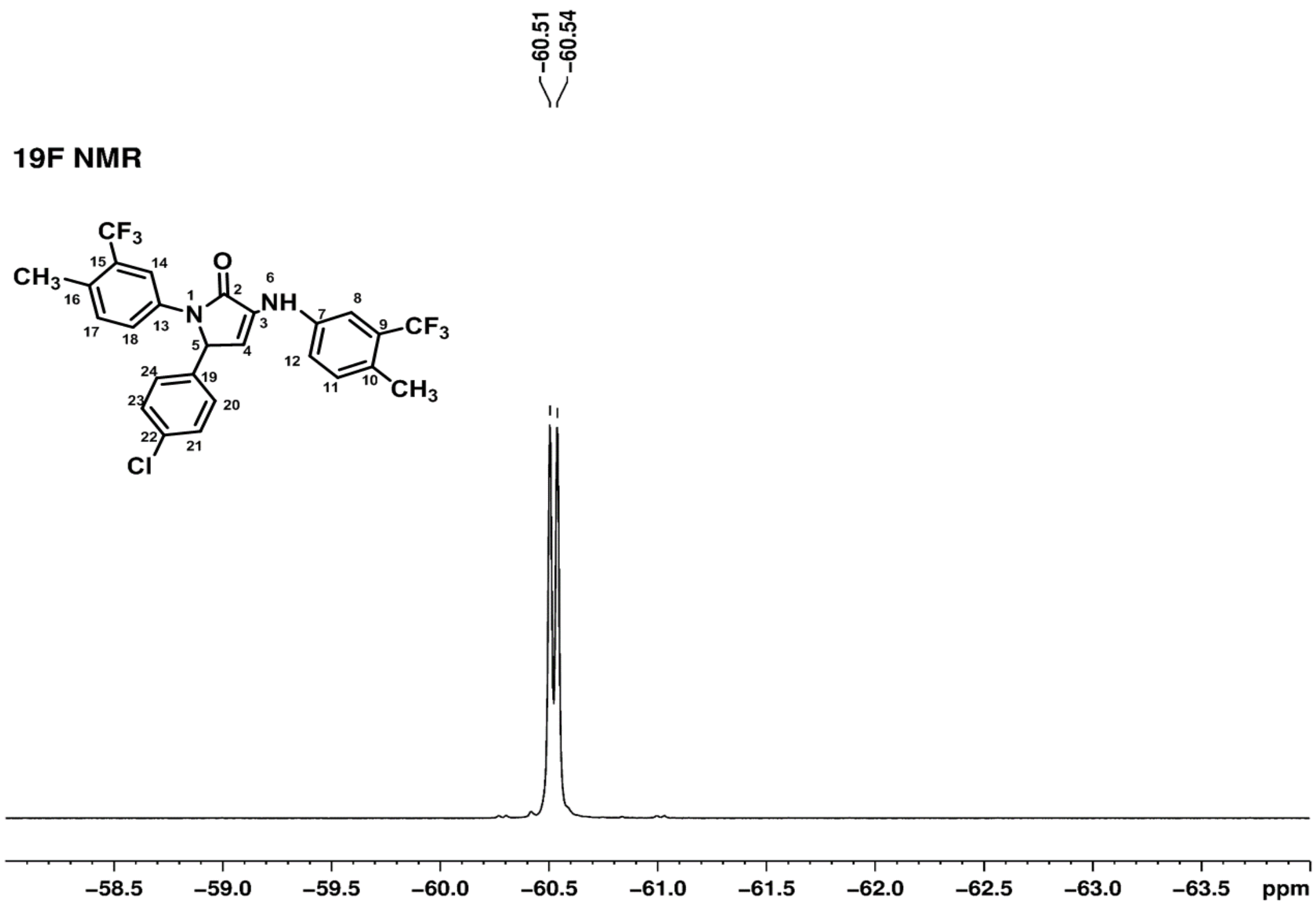

Figure 15S. ^{19}F-NMR spectrum **4o** recorded in DMSO-d$_6$, at 376.4 MHz.

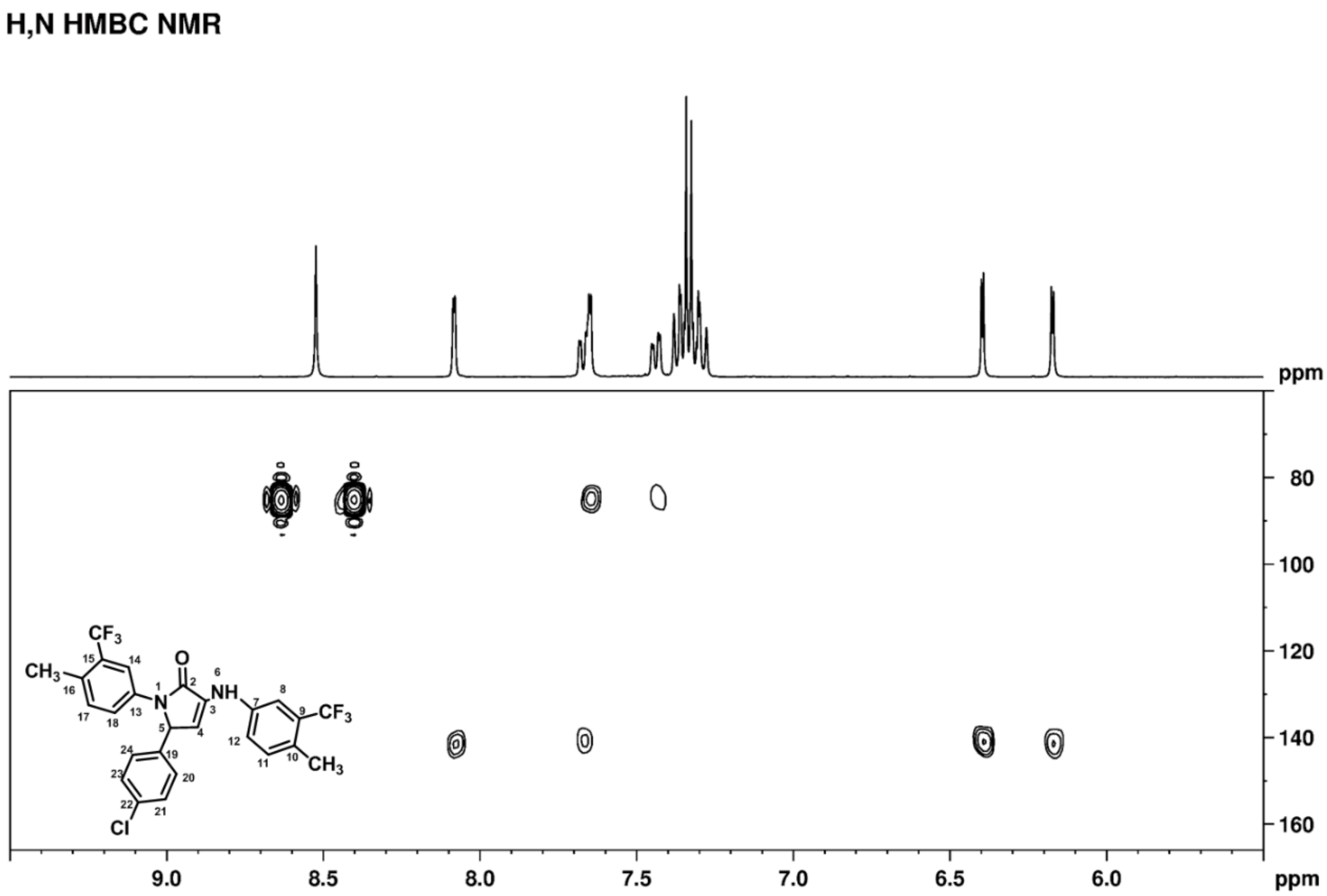

Figure 16S. H,N-HMBC spectrum **4o** recorded in DMSO-d$_6$, at 40.5 MHz.

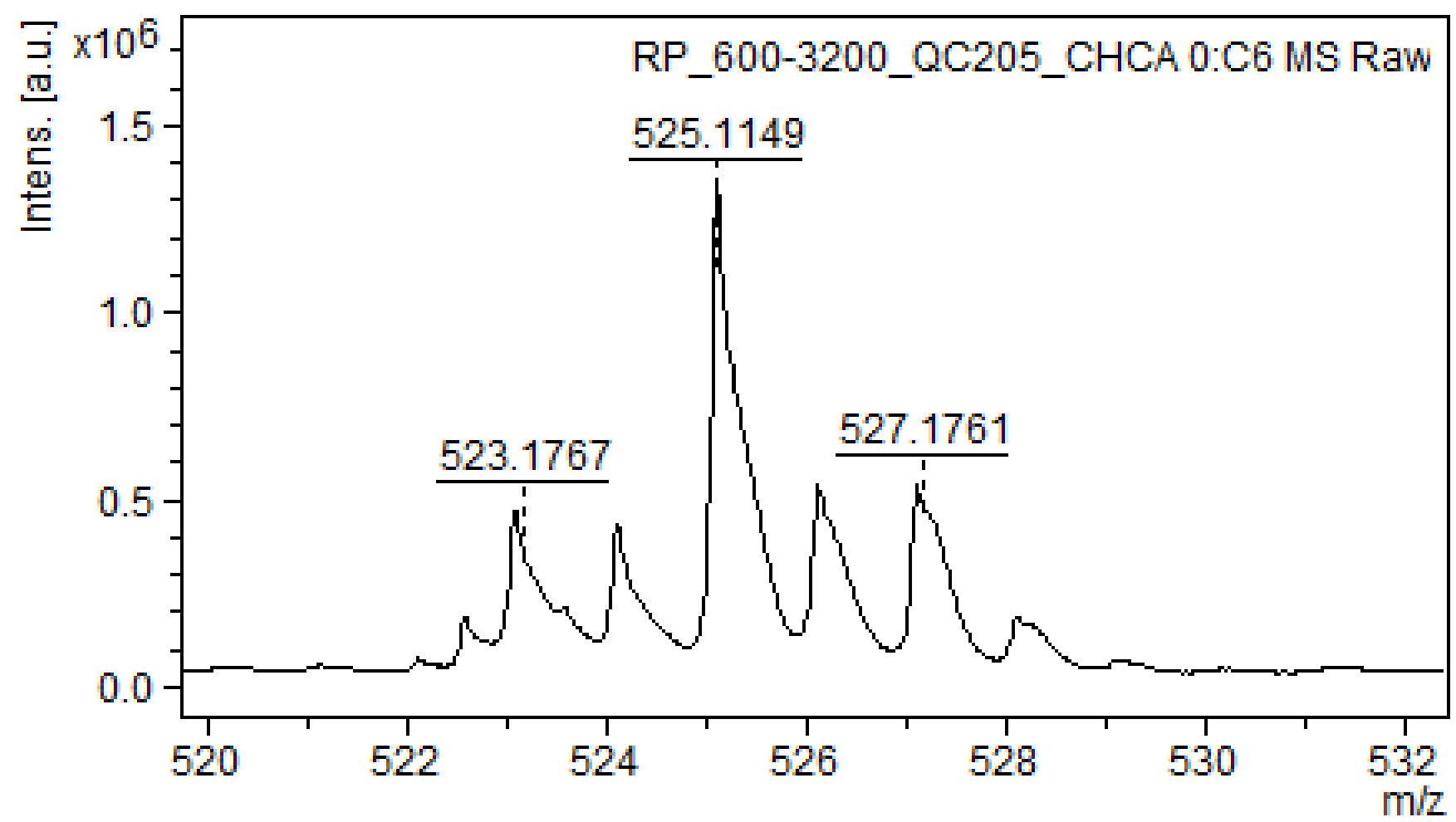

Figure 17S. MALDI-MS spectrum for **4o**

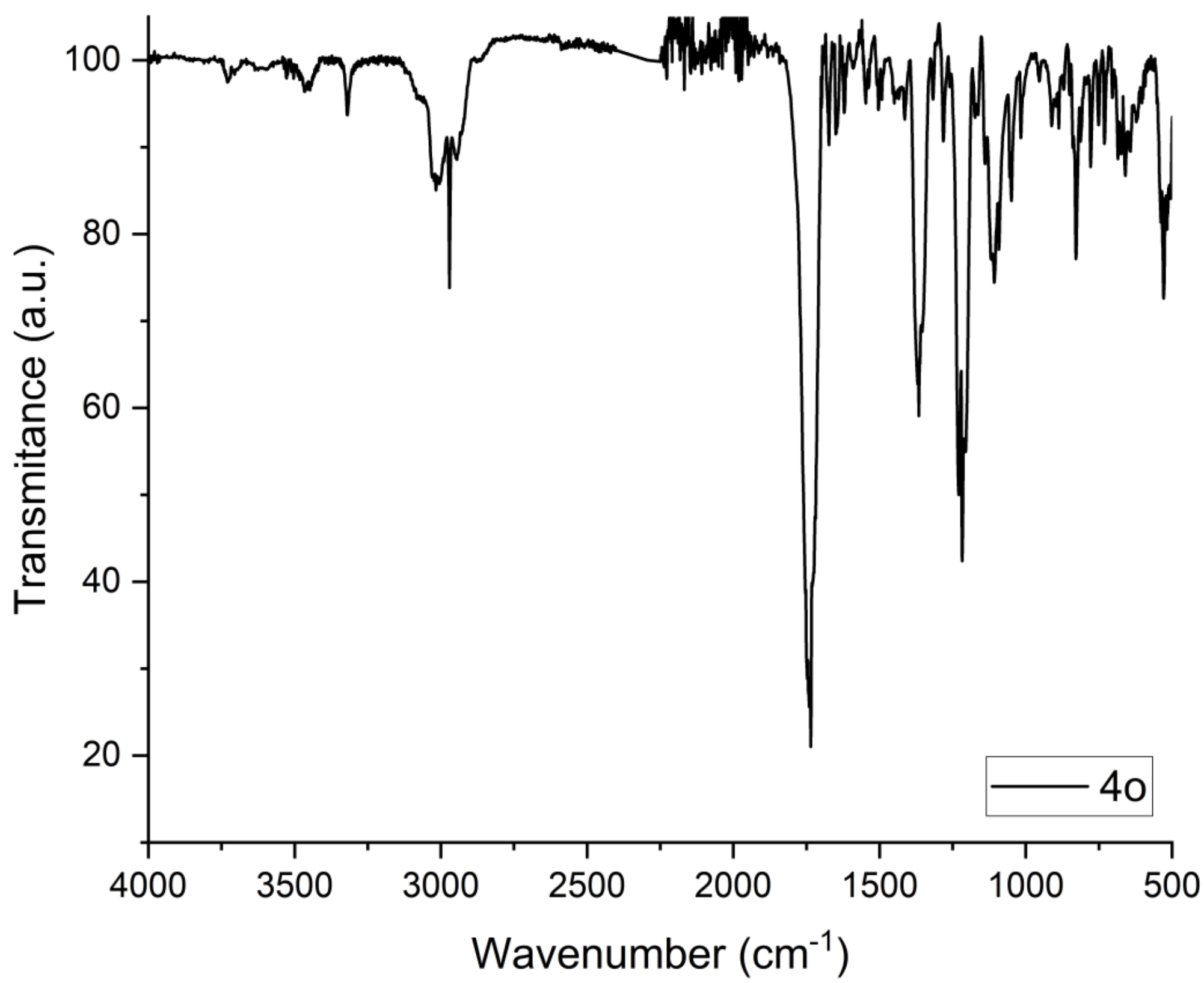

Figure 18S. FT-IR spectra for compound **4o**

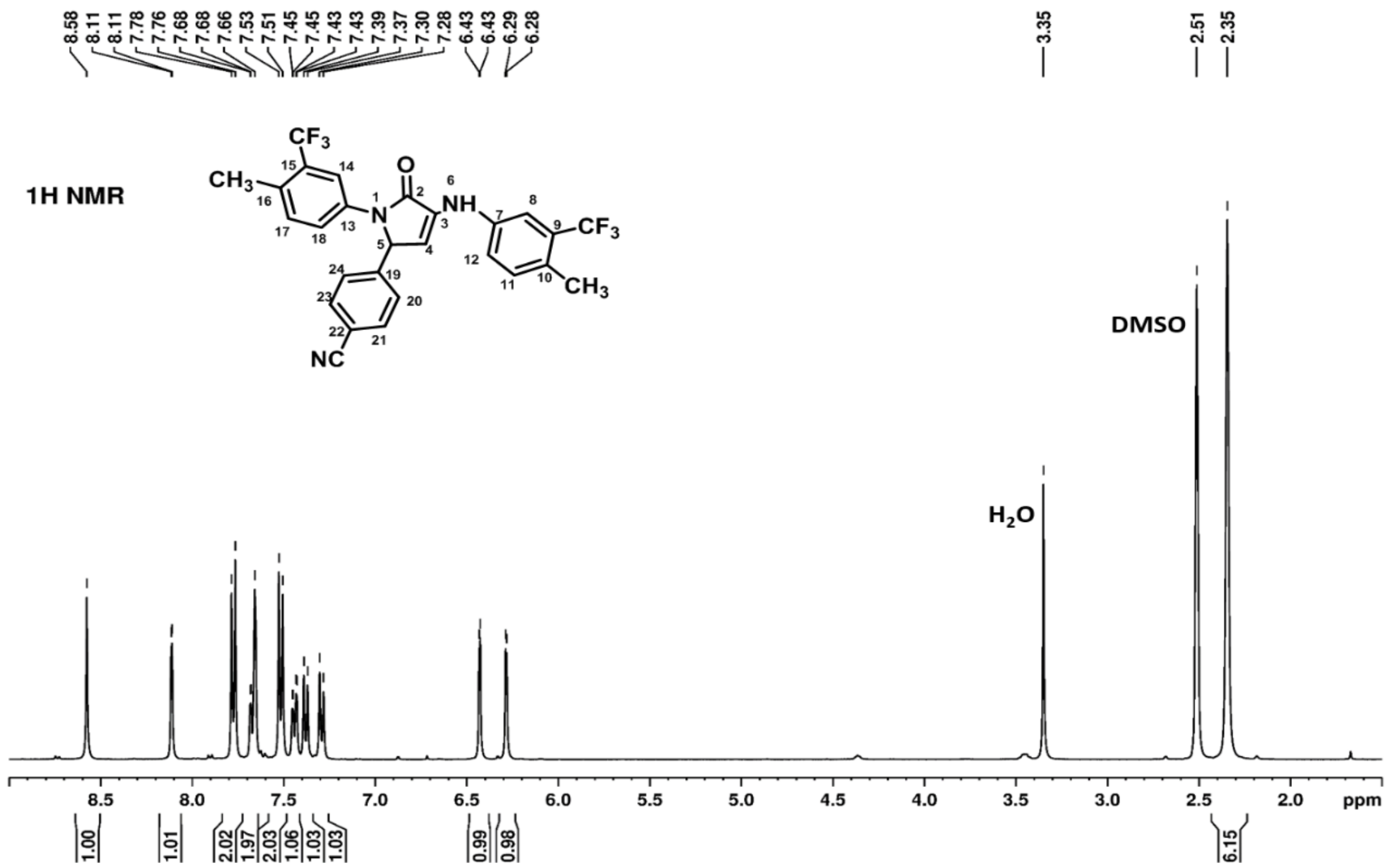

Figure 19S. ^{1}H-NMR spectrum **4t** recorded in DMSO-d$_6$, at 400.1 MHz.

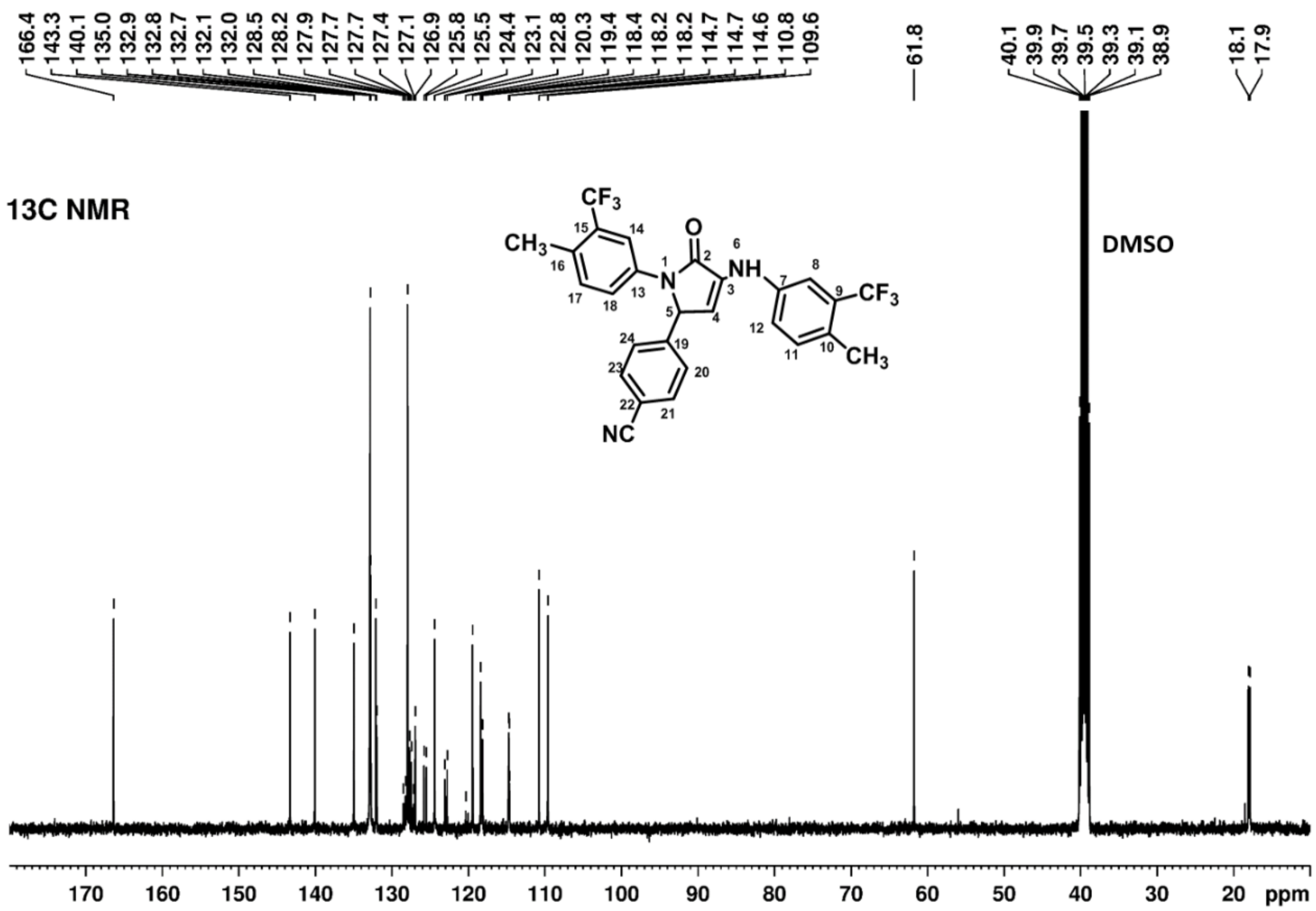

Figure 20S. ^{13}C-NMR spectrum **4t** recorded in DMSO-d$_6$, at 100.6 MHz.

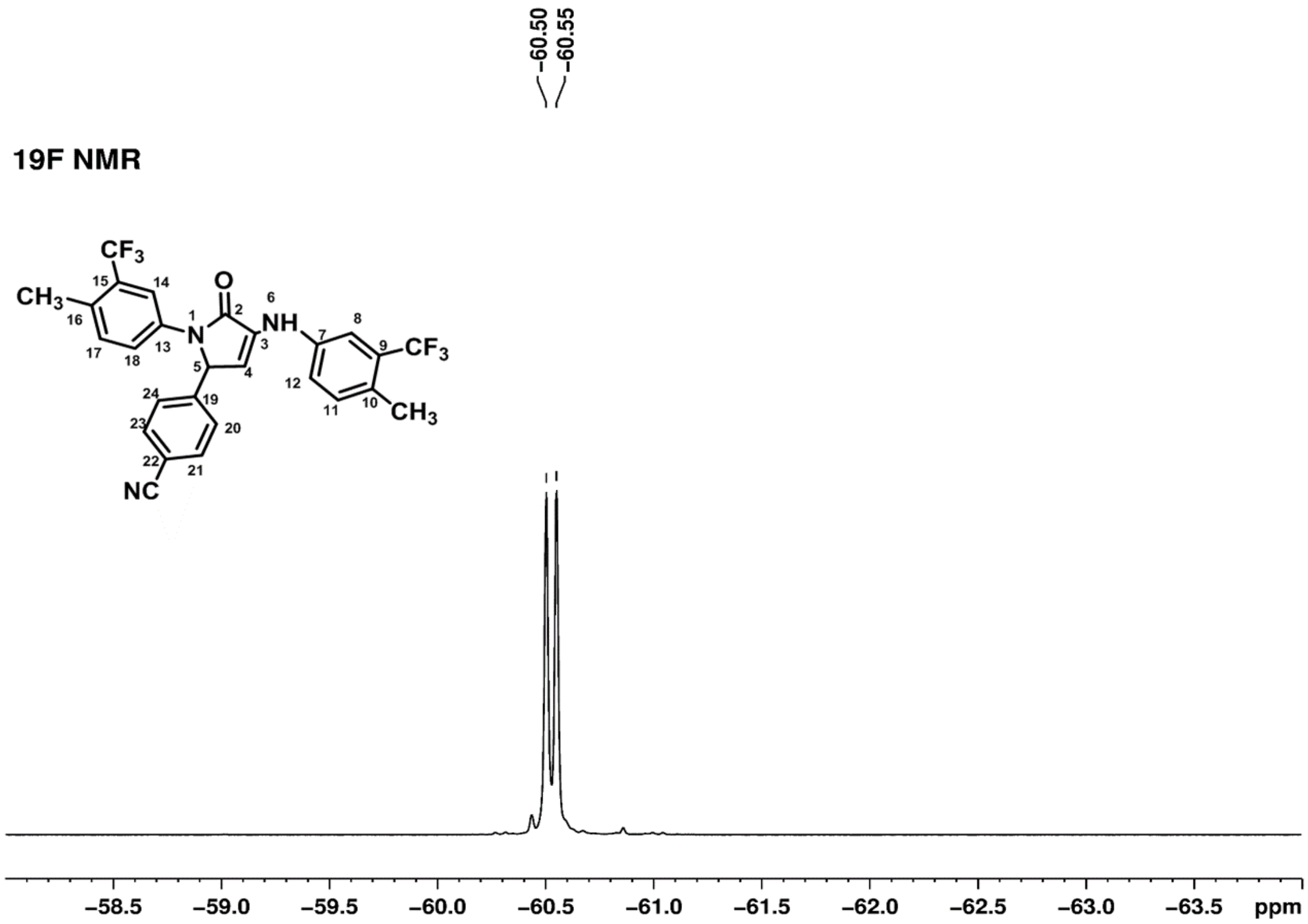

Figure 21S. ^{19}F-NMR spectrum **4t** recorded in DMSO-d$_6$, at 376.4 MHz.

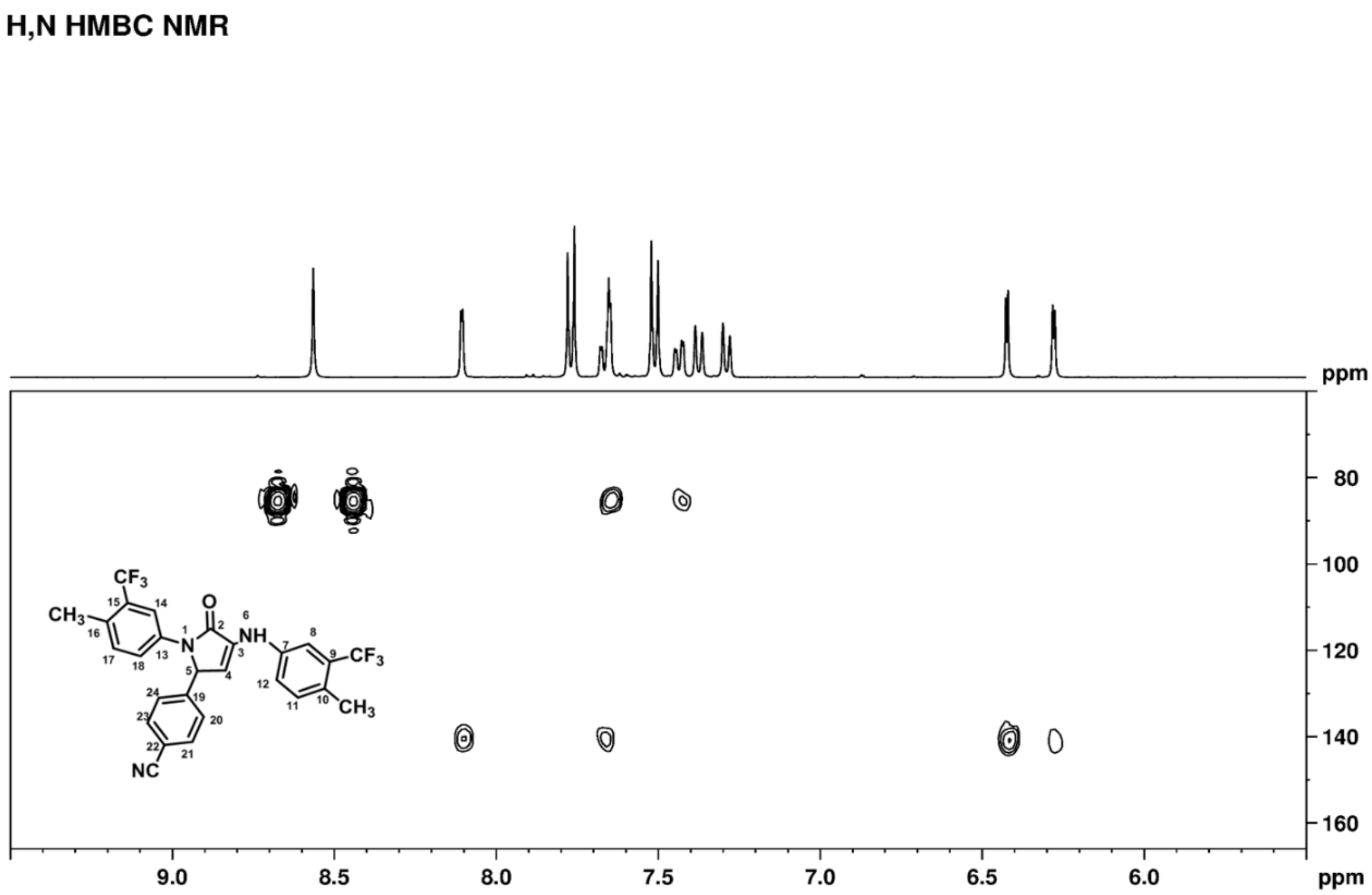

Figure 22S. H,N-HMBC spectrum **4t** recorded in DMSO-d$_6$, at 40.5 MHz.

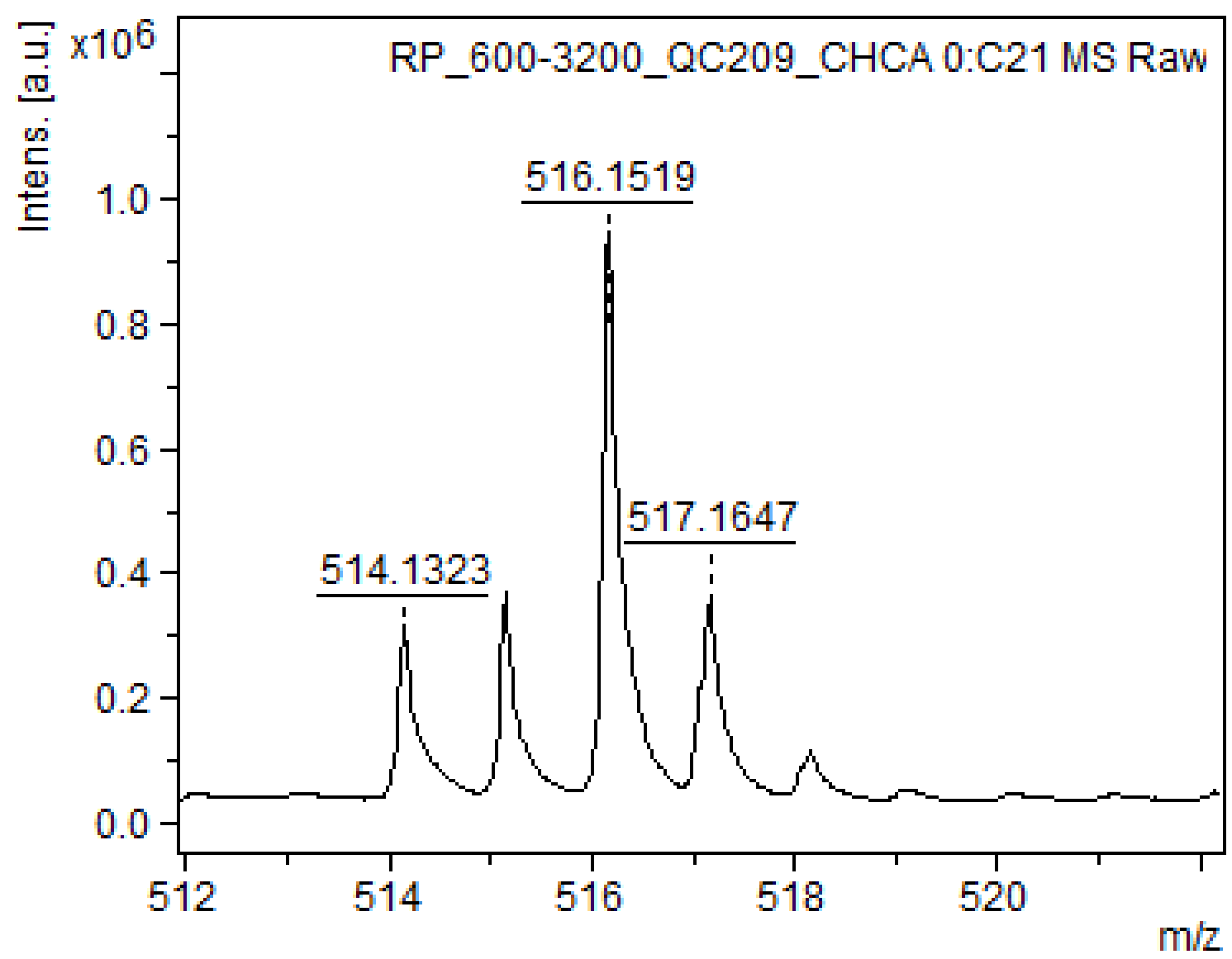

Figure 23S. MALDI-MS spectrum for **4t**

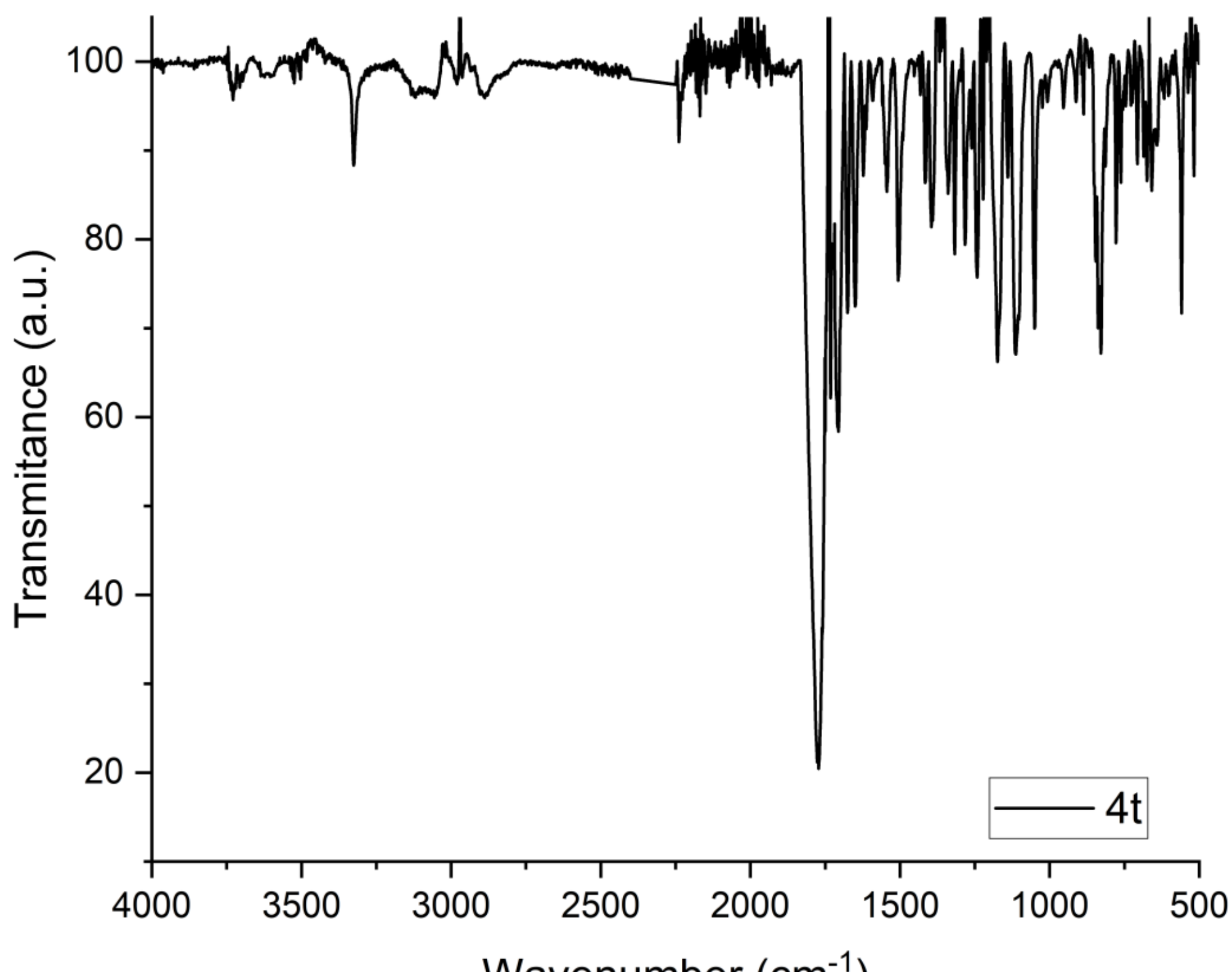

Figure 24S. FT-IR spectra for compound **4t**

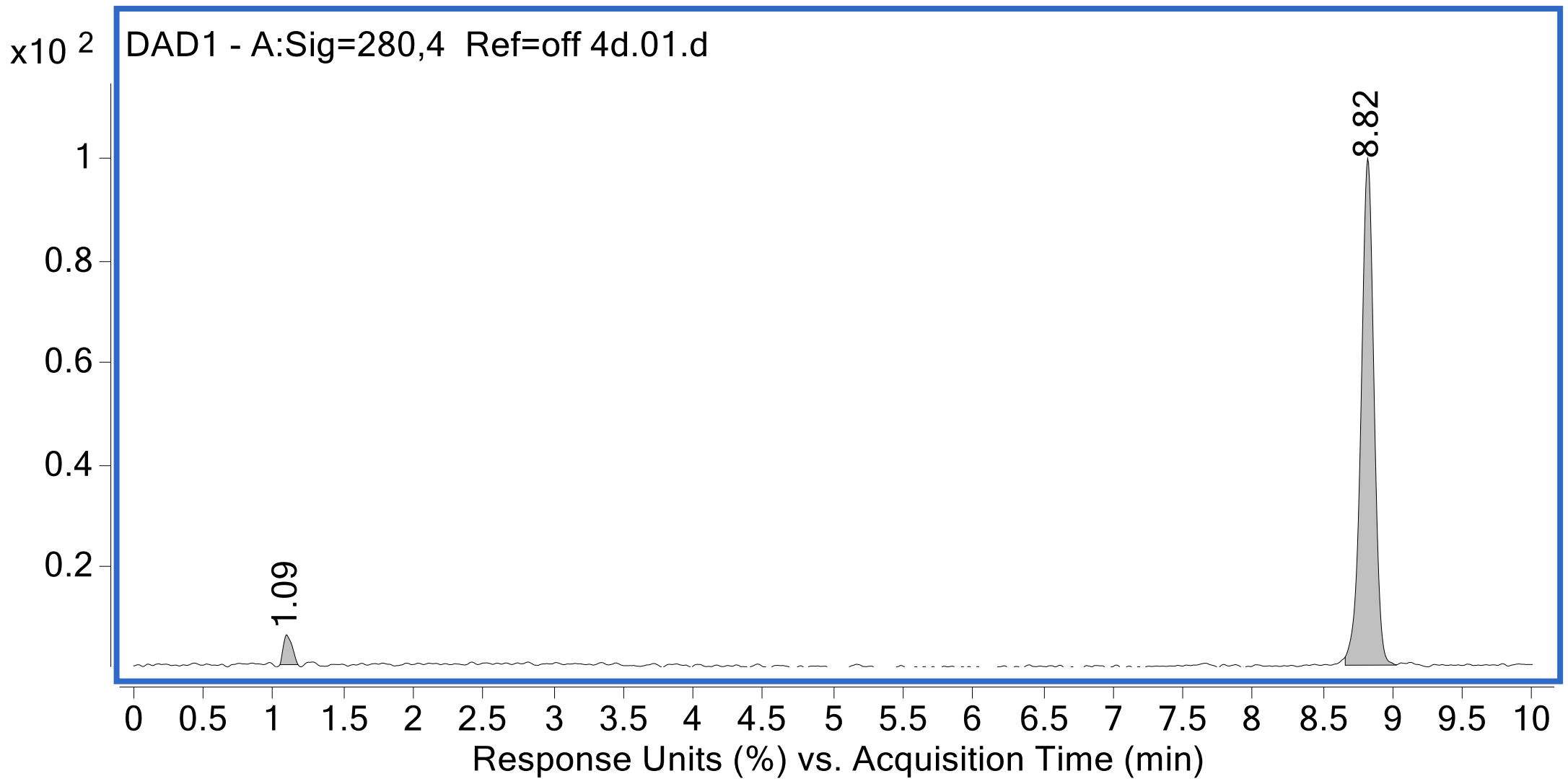

Figure S25. HPLC spectra for compound **4d** (280nm)

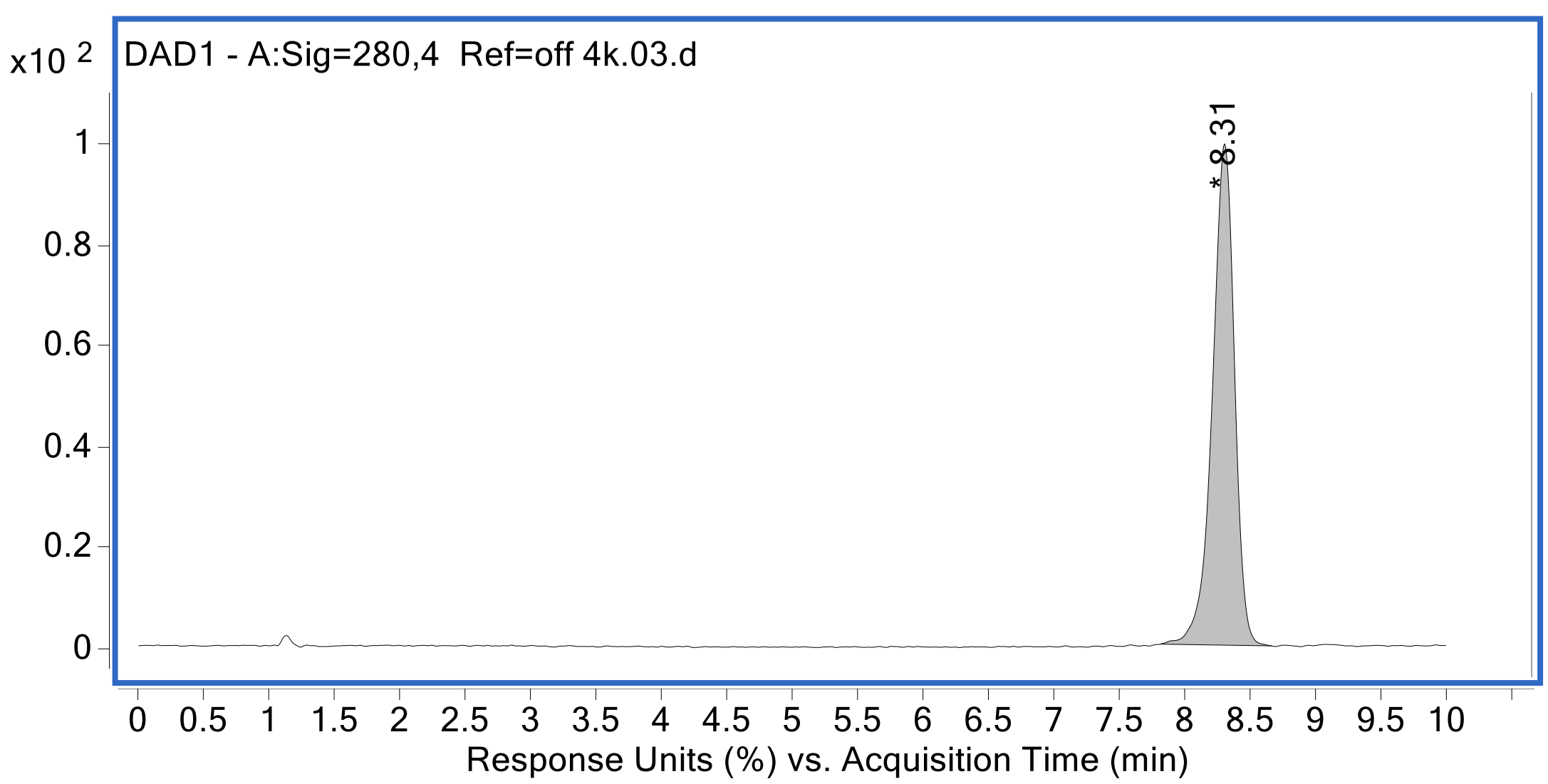

Figure S26. HPLC spectra for compound **4k** (280nm)

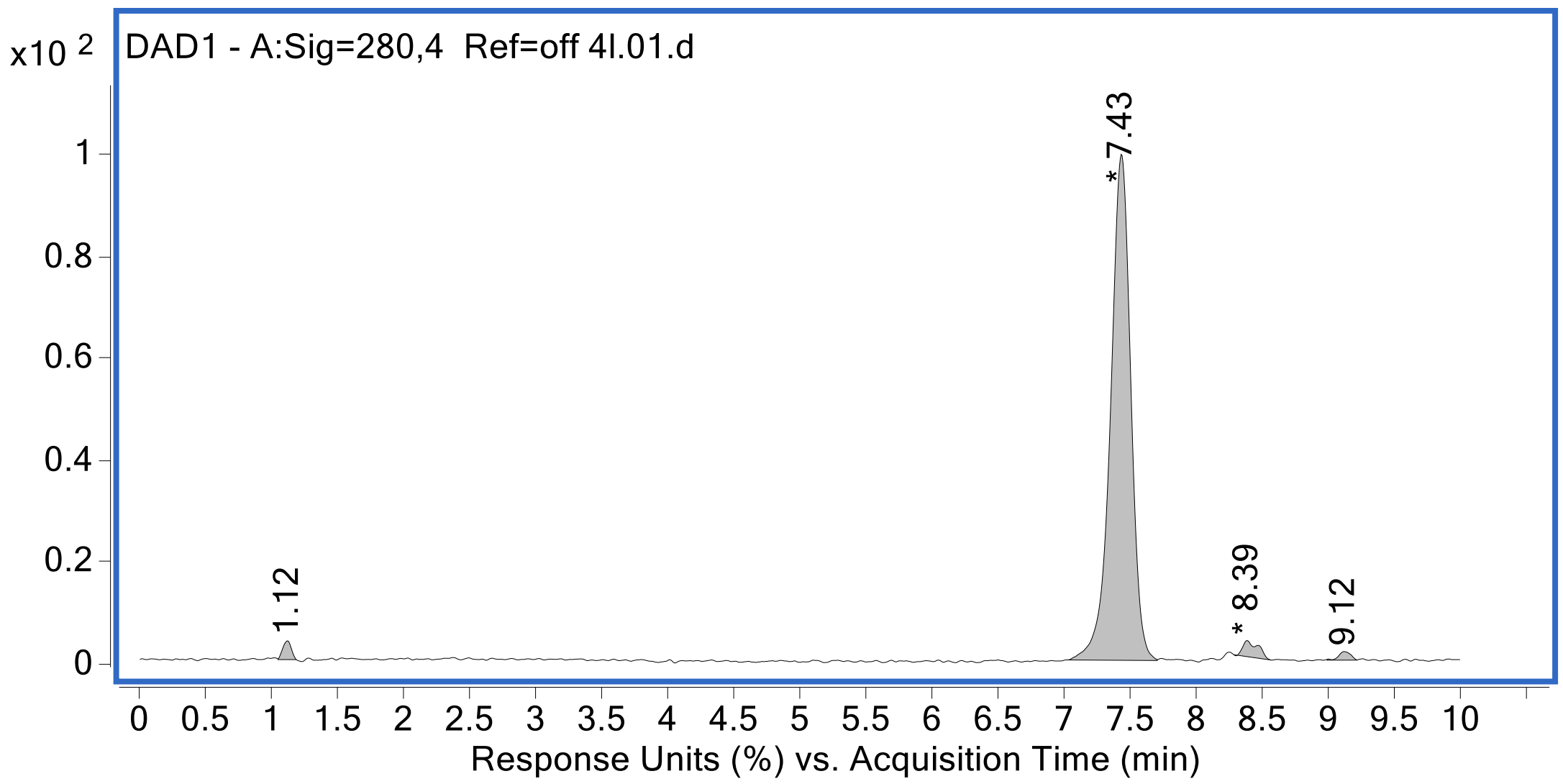

Figure S27. HPLC spectra for compound **4l** (280nm)

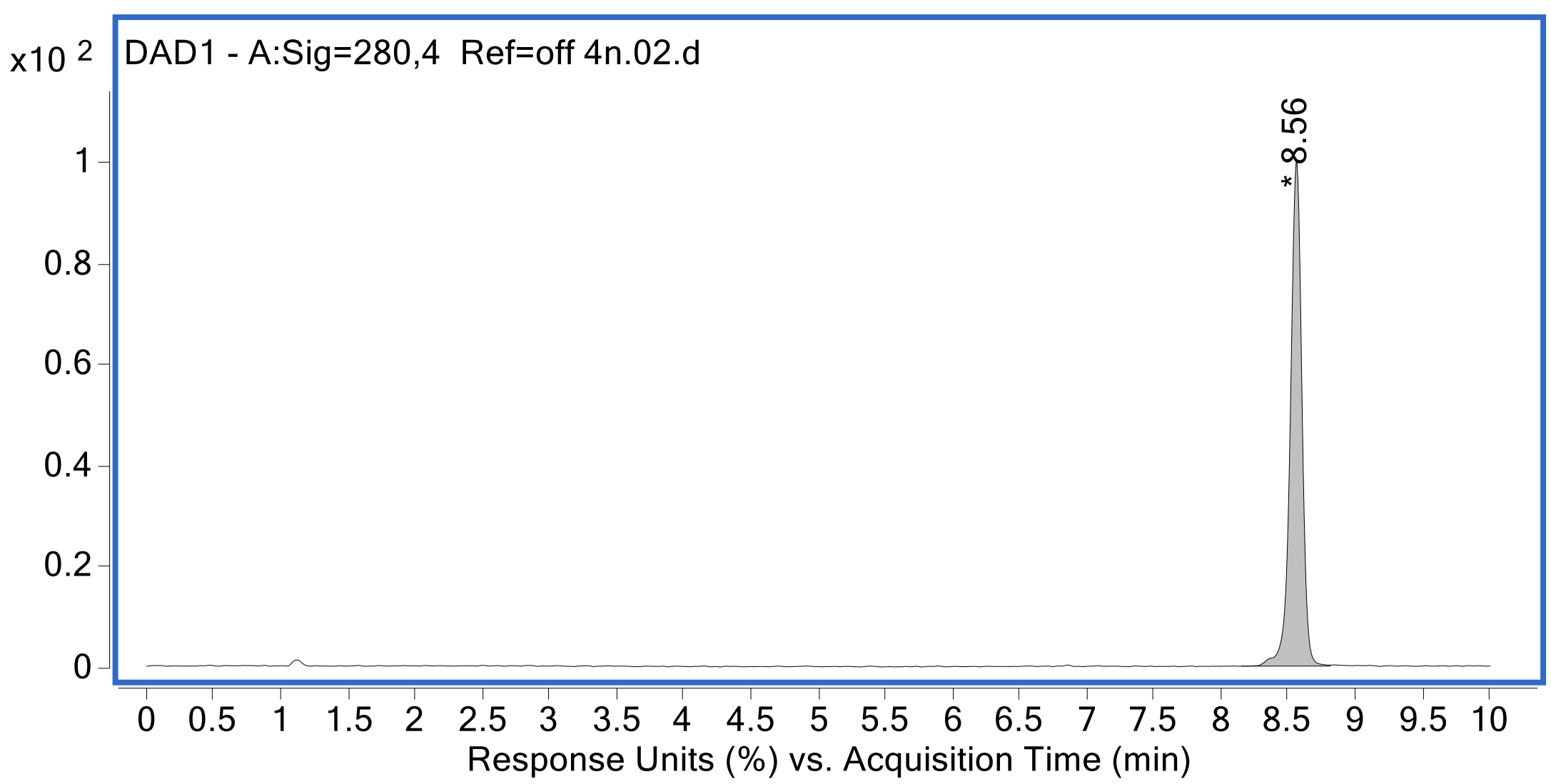

Figure S28. HPLC spectra for compound **4n** (280nm)

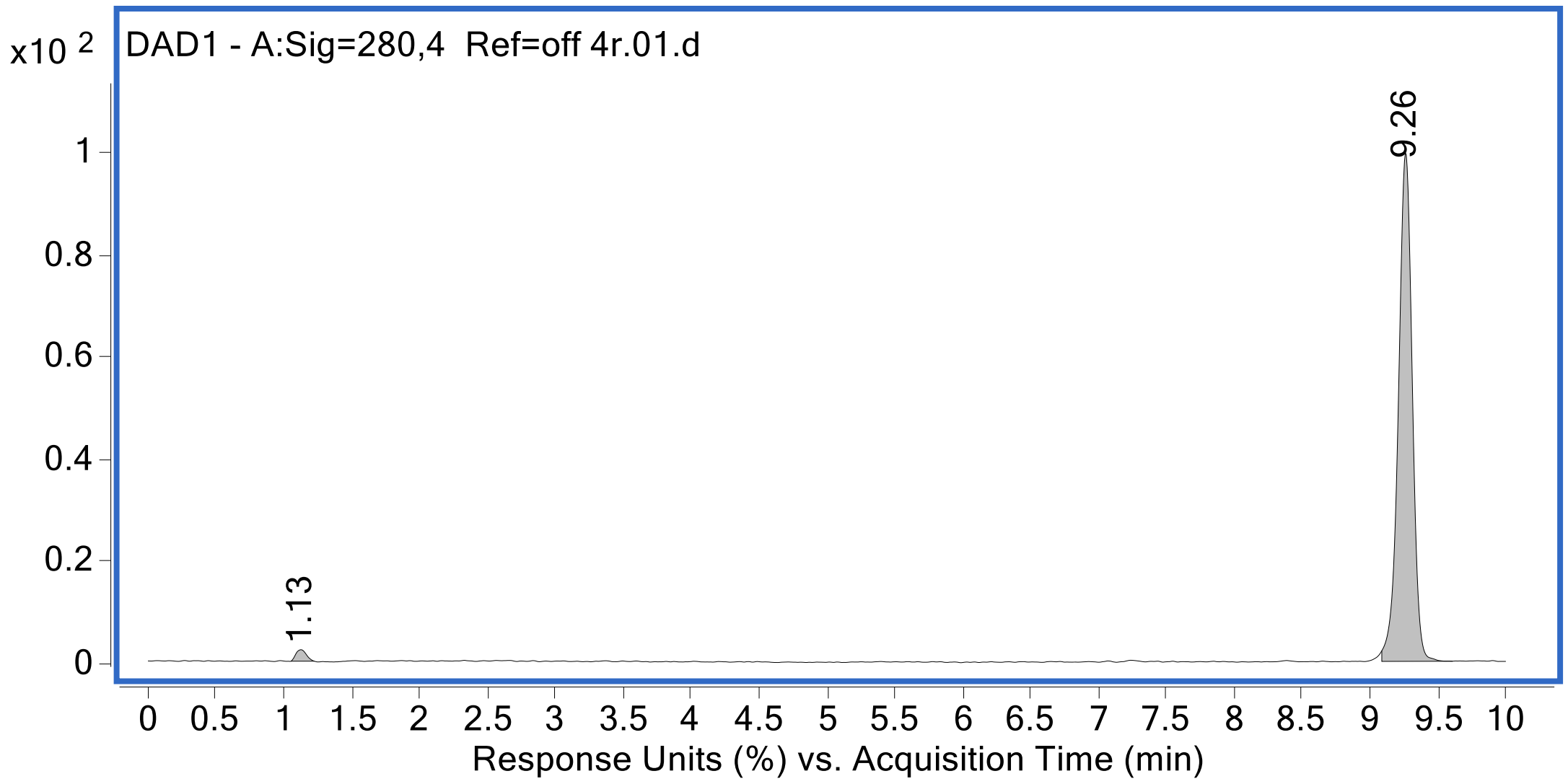

Figure S29. HPLC spectra for compound **4r** (280nm)

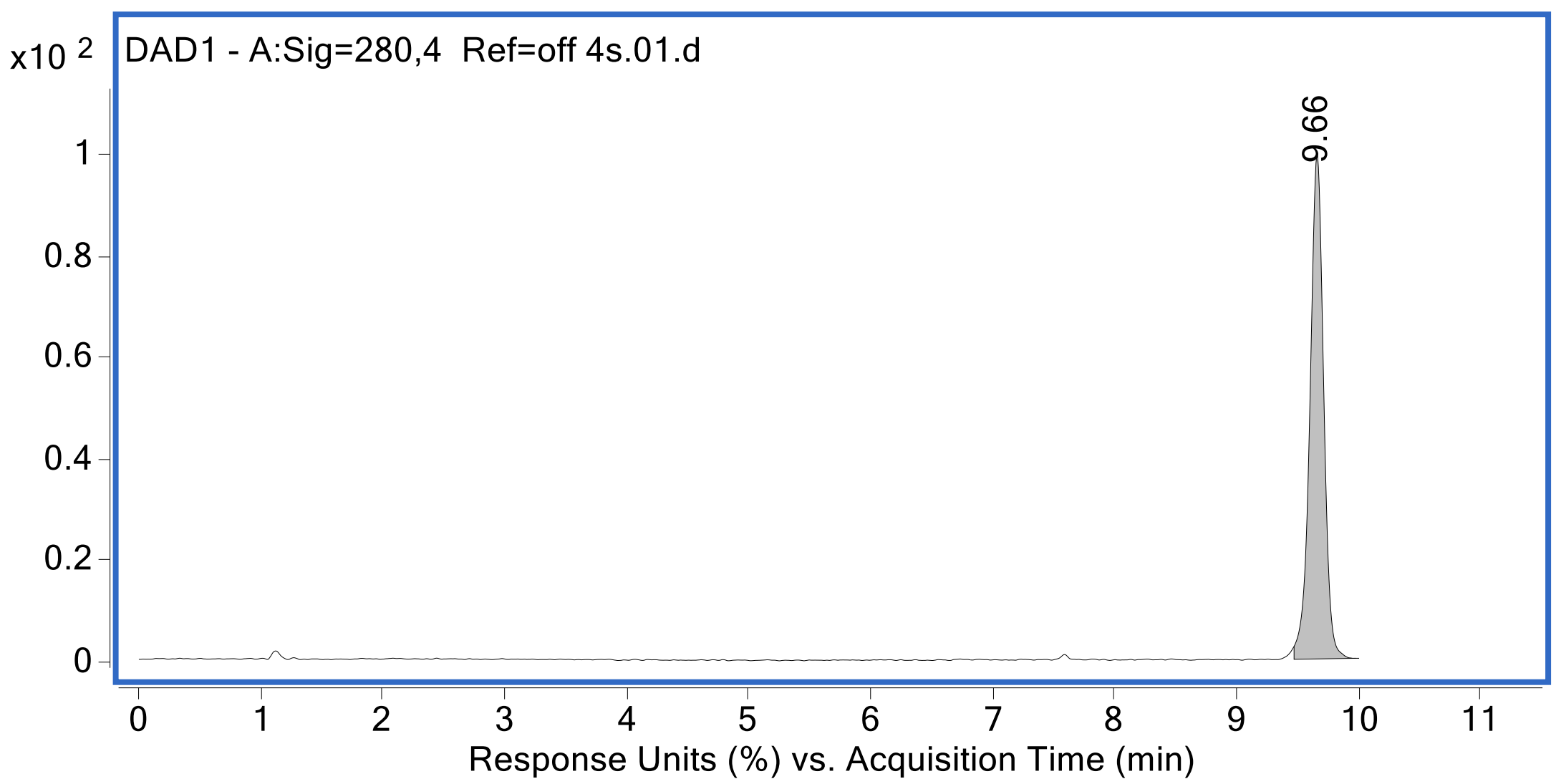

Figure S30. HPLC spectra for compound **4s** (280nm)

Table 1S. Radar diagram for compounds

Compound smiles	Radar diagram
**(4a):** O=C1N(C2=CC=C(C)C(C(F)(F)F)=C2)C(C3=CC(OC)=C(O)C(OC)=C3)C=C1NC4=CC=C(C)C(C(F)(F)F)=C4	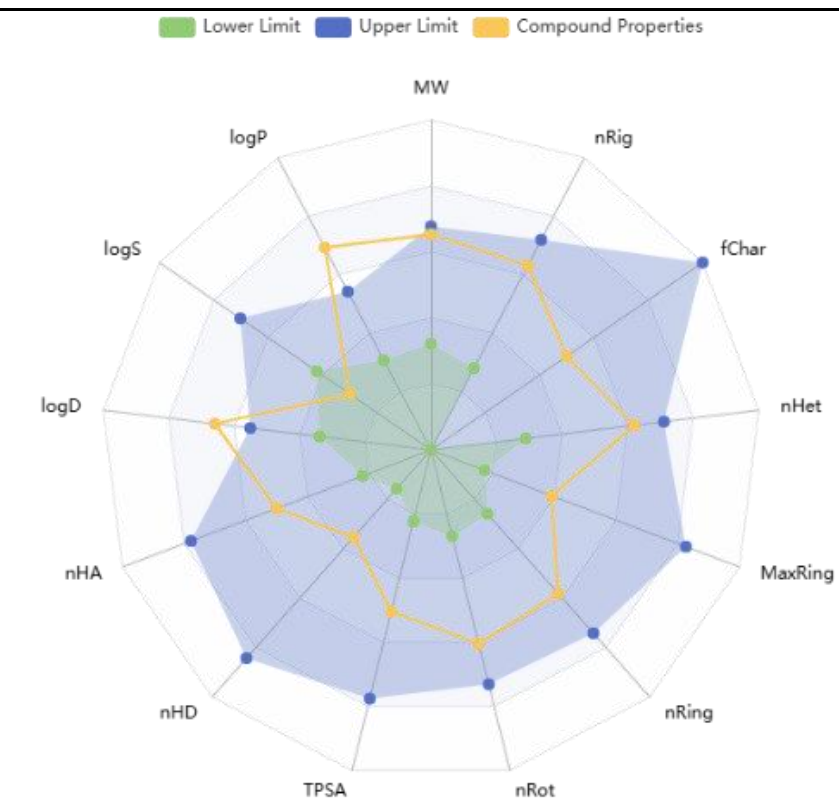
**(4b):** O=C1N(C2=CC=C(C)C(C(F)(F)F)=C2)C(C3=CC=C(OC)C(O)=C3)C=C1NC4=CC=C(C)C(C(F)(F)F)=C4	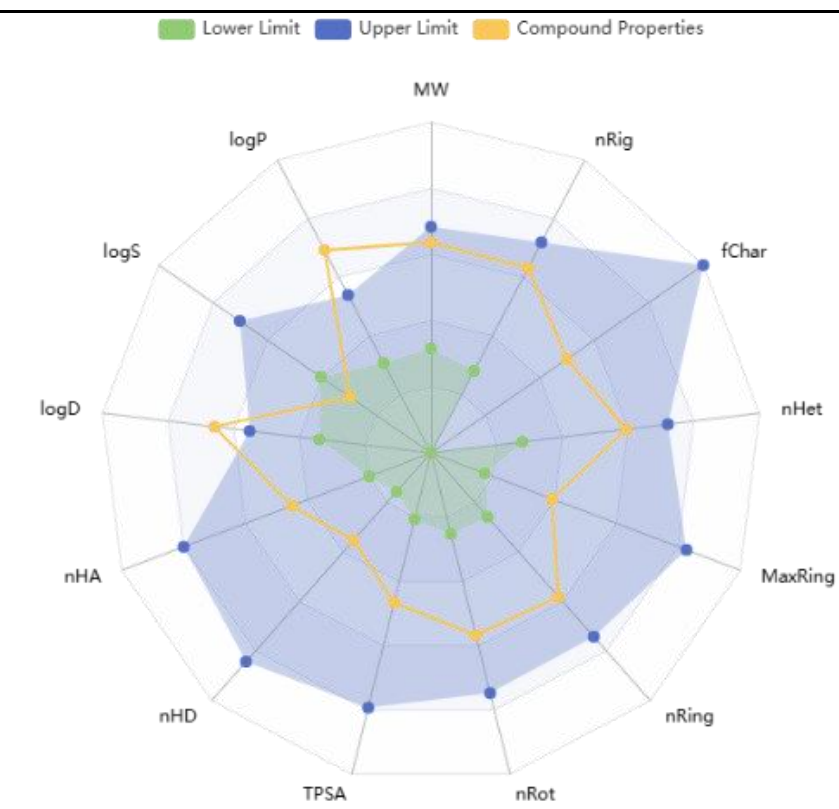
**(4c):** O=C1N(C2=CC=C(C)C(C(F)(F)F)=C2)C(C3=CC(OC)=C(OC)C=C3OC)C=C1NC4=CC=C(C)C(C(F)(F)F)=C4	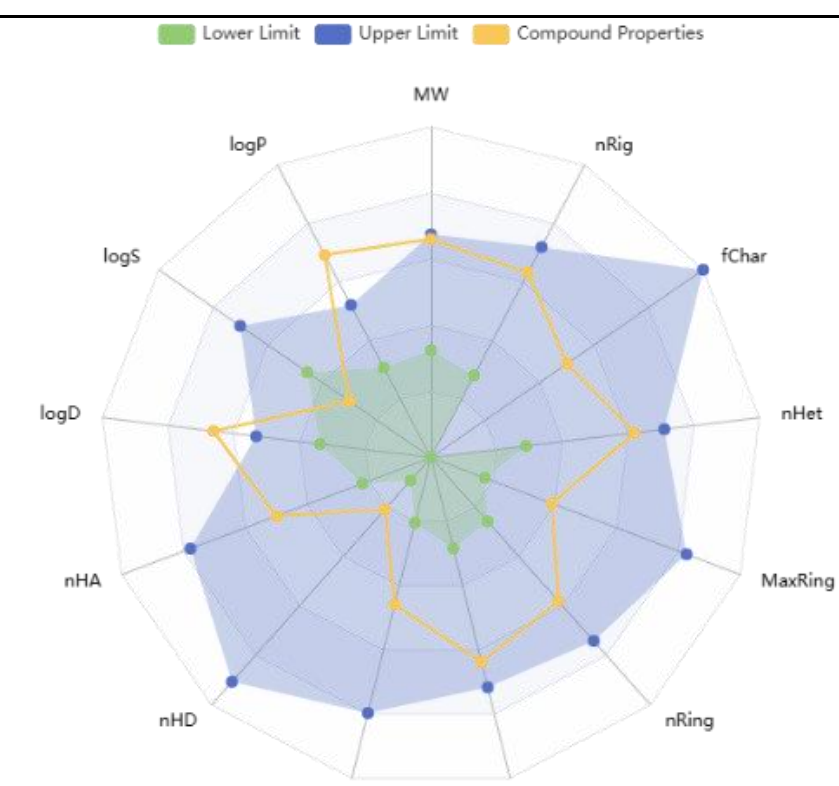

<table>
<tr>
<td>(4d):
O=C1N(C2=CC=C(C)C
(C(F)(F)F)=C2)C(C3=CC=C(OC)C
(OC)=C3OC)C=C1NC4=CC=C(C)
C(C(F)(F)F)=C4</td>
<td>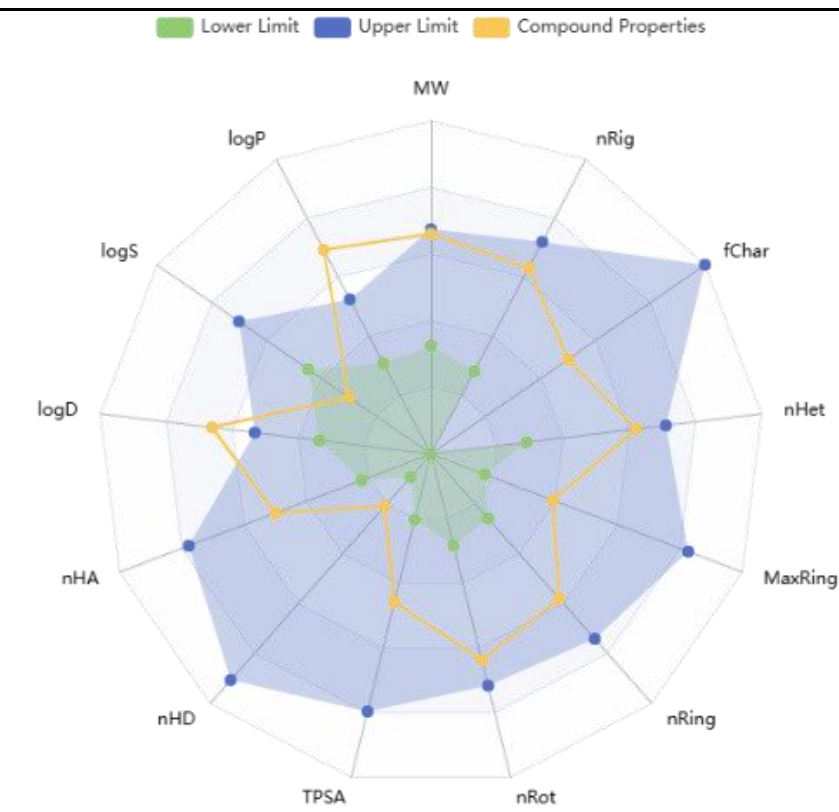
</td>
</tr>
<tr>
<td>(4e): O=C1N(C2=CC=C(C)C
(C(F)(F)F)=C2)C(C3=CC(OC)=C(OC)
C(OC)=C3)C=C1NC4=CC=C(C)C
(C(F)(F)F)=C4</td>
<td>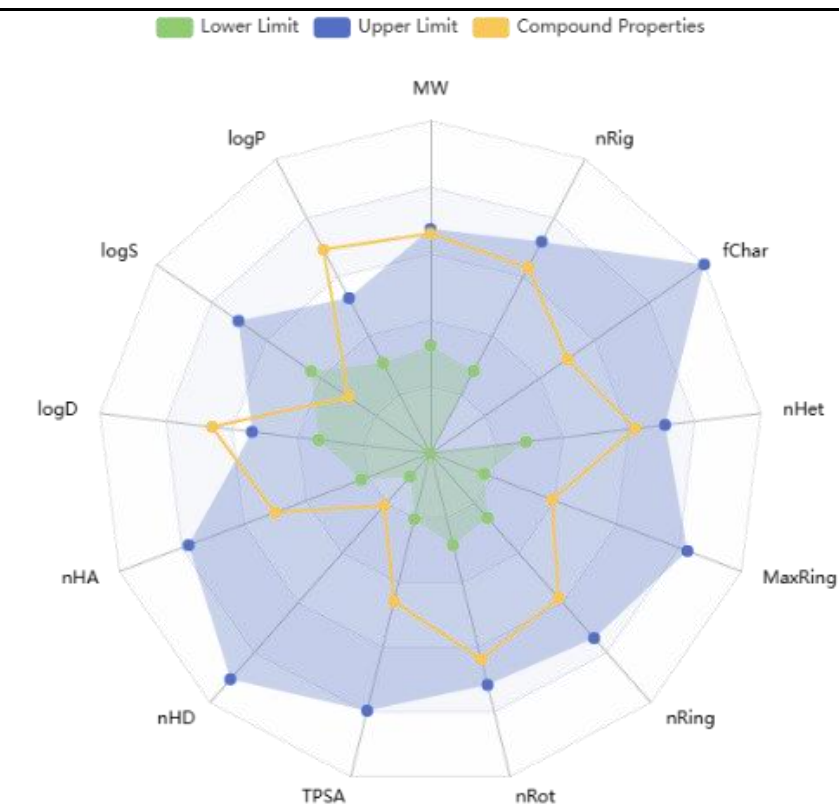
</td>
</tr>
<tr>
<td>(4f): O=C1N(C2=CC=C(C)C
(C(F)(F)F)=C2)C(C3=CC
(OC)=CC=C3OC)C=C1NC4=CC=C(C)
C(C(F)(F)F)=C4</td>
<td>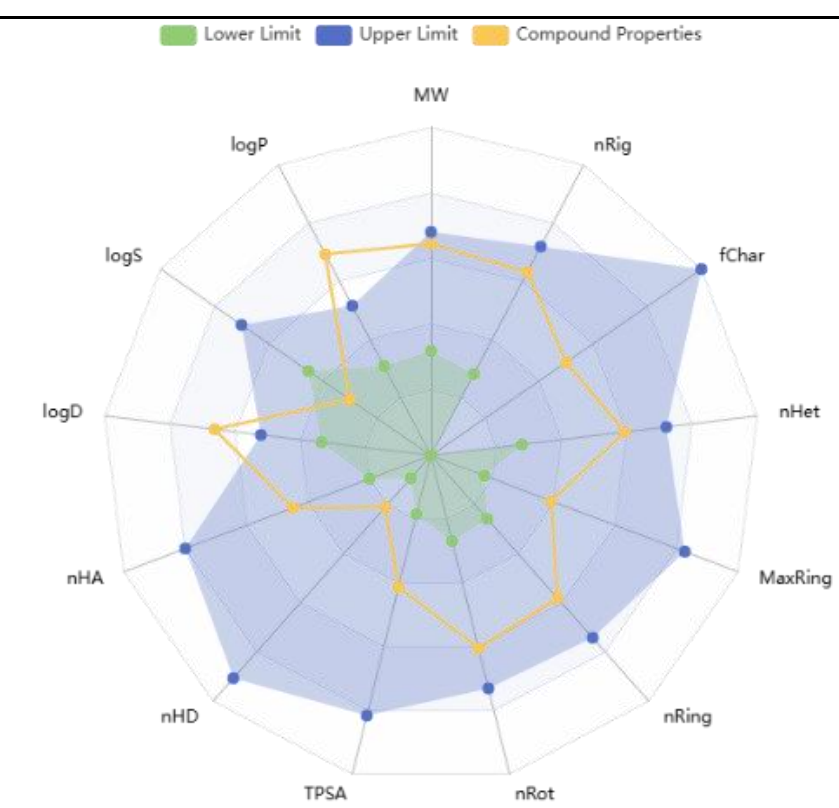
</td>
</tr>
</table>

**(4g):** O=C1N(C2=CC=C(C)C(C(F)(F)F)=C2)C(C3=CC=C(OC)C(OC)=C3)C=C1NC4=CC=C(C)C(C(F)(F)F)=C4	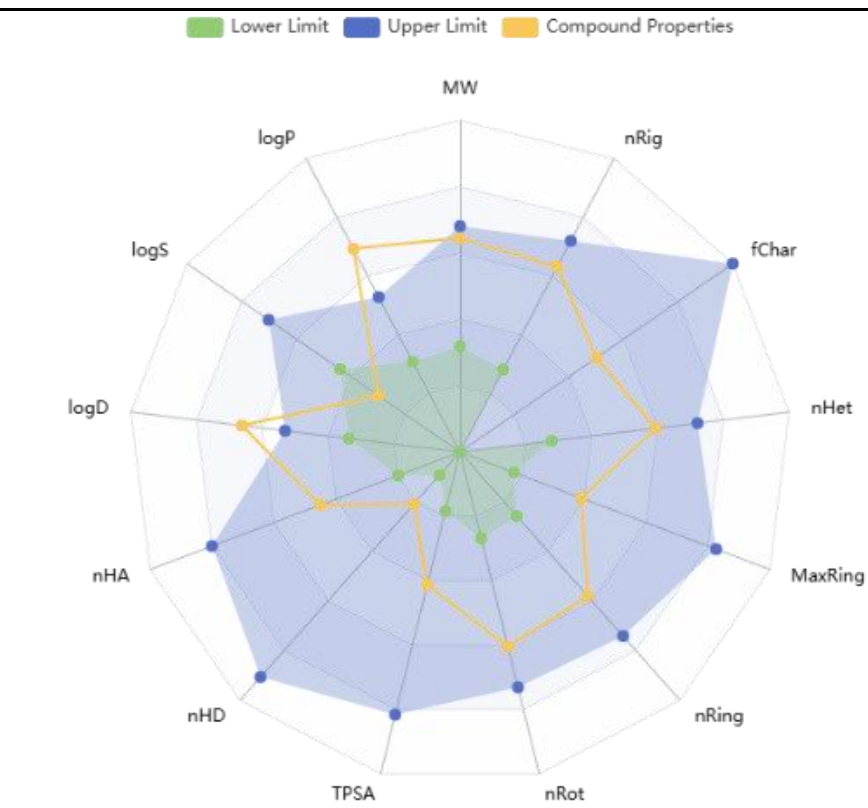
**(4h):** O=C1N(C2=CC=C(C)C(C(F)(F)F)=C2)C(C3=CC=CC=C3OC)C=C1NC4=CC=C(C)C(C(F)(F)F)=C4	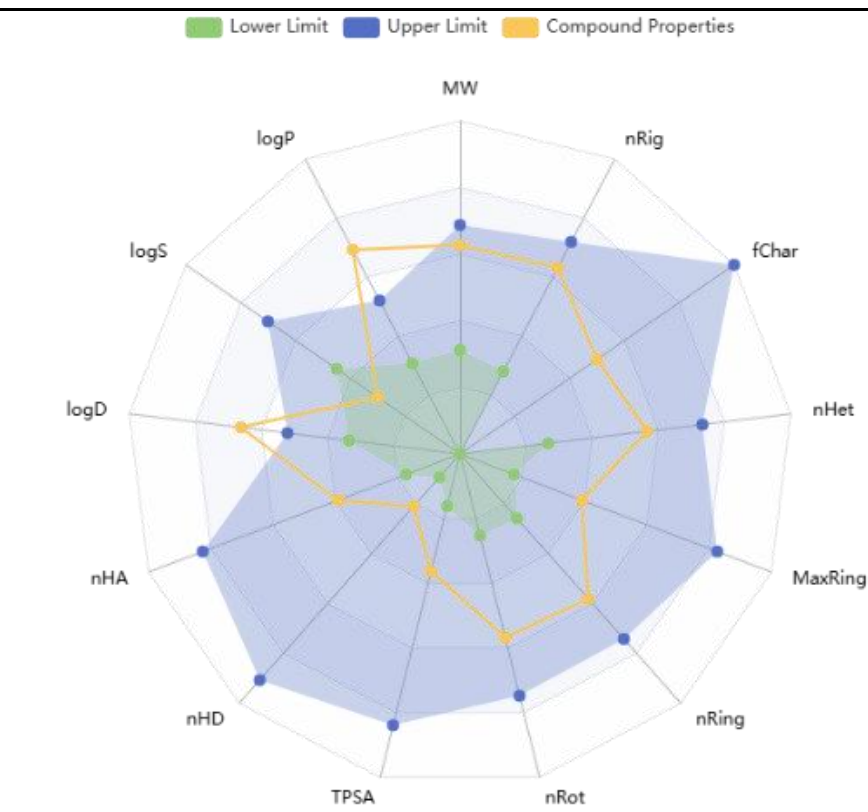
**(4i):** O=C1N(C2=CC=C(C)C(C(F)(F)F)=C2)C(C3=CC=C(OC)C=C3)C=C1NC4=CC=C(C)C(C(F)(F)F)=C4	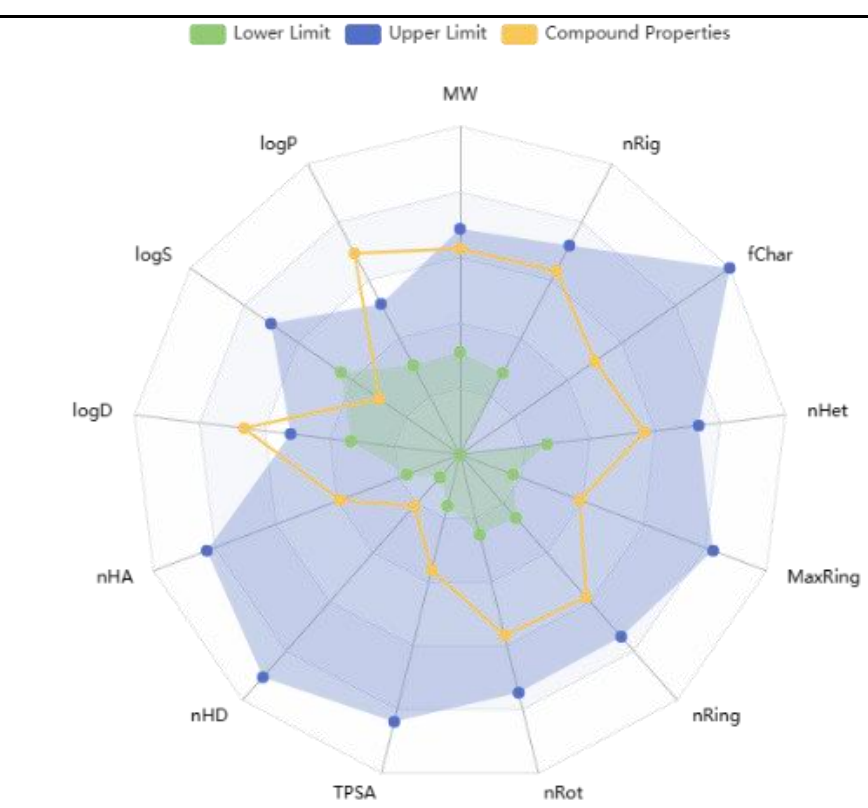

**(4j):** O=C1N(C2=CC=C(C)C(C(F)(F)F)=C2)C(C3=CC=CO3)C=C1NC4=CC=C(C)C(C(F)(F)F)=C4	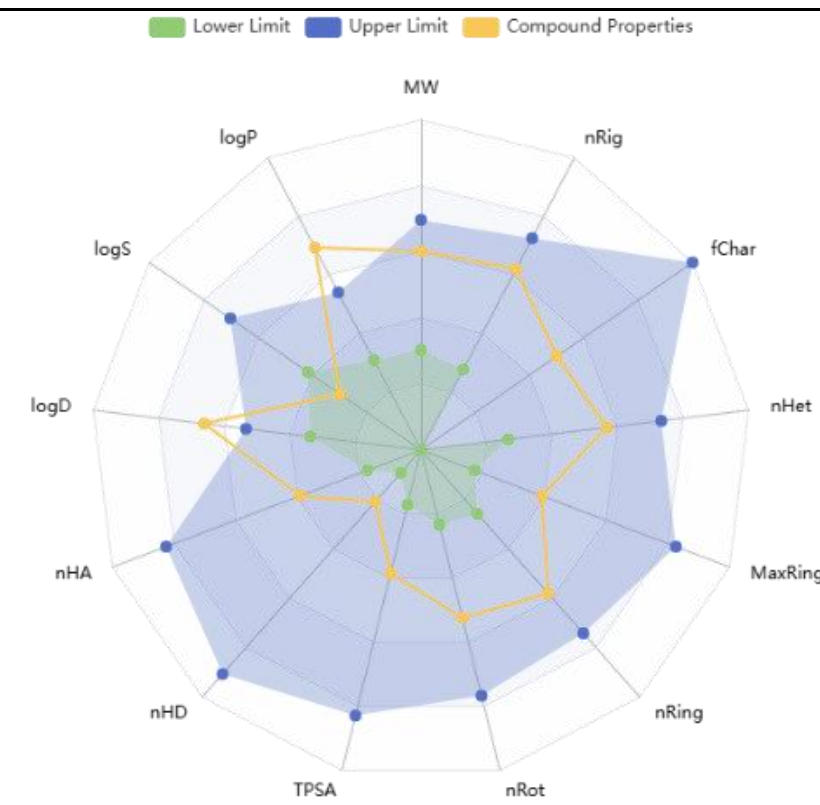
**(4k):** O=C1N(C2=CC=C(C)C(C(F)(F)F)=C2)C(C3=CC=C(C(C)C)C=C3)C=C1NC4=CC=C(C)C(C(F)(F)F)=C4	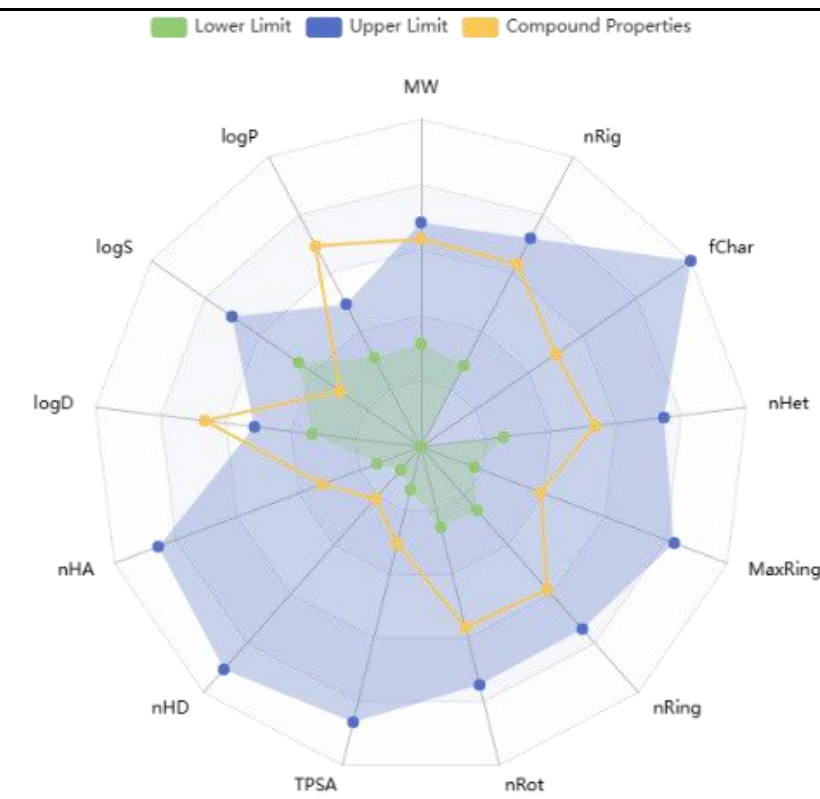
**(4l):** O=C1N(C2=CC=C(C)C(C(F)(F)F)=C2)C(C3=CC=C(C(C)(C)C)C=C3)C=C1NC4=CC=C(C)C(C(F)(F)F)=C4	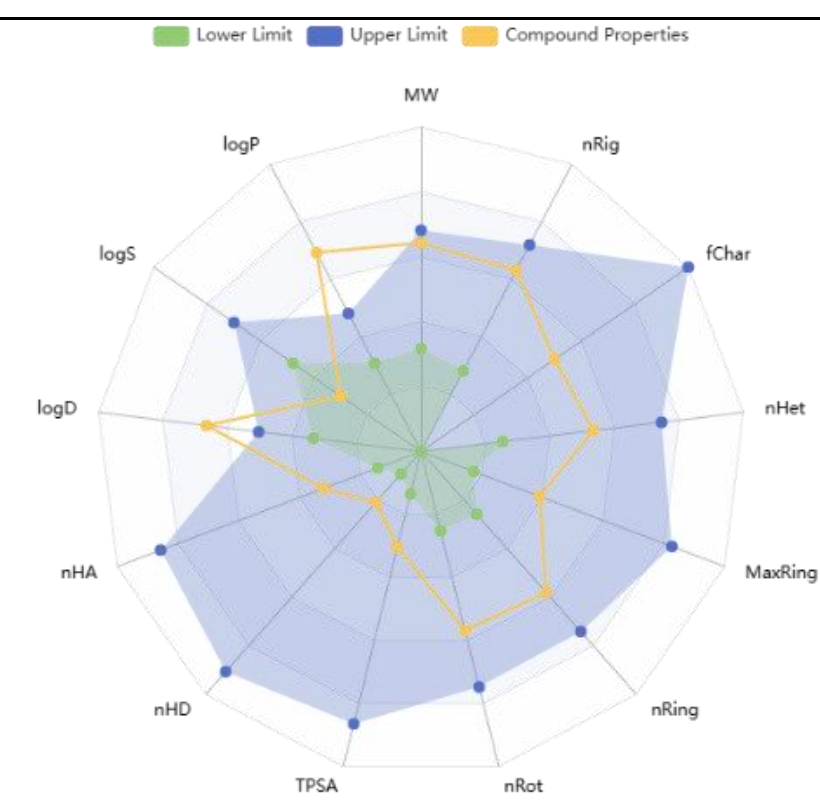

<table>
<tr>
<td>(4m): O=C1N(C2=CC=C(C)C(C(F)(F)F)=C2)C(C3=CC=CC=C3)C=C1NC4=CC=C(C)C(C(F)(F)F)=C4</td>
<td>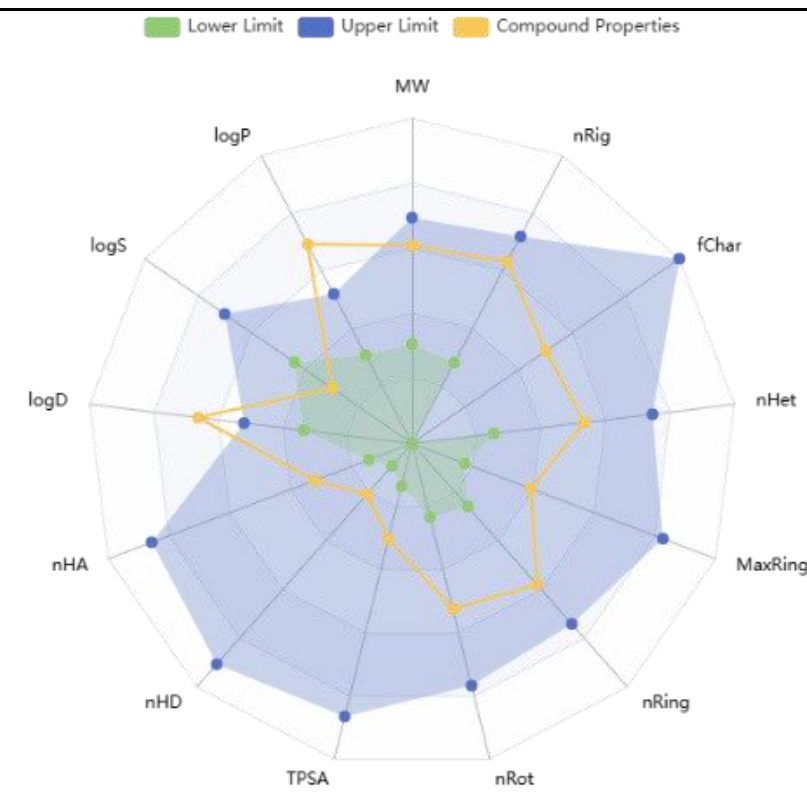
</td>
</tr>
<tr>
<td>(4n): O=C1N(C2=CC=C(C)C(C(F)(F)F)=C2)C(C3=CC=C(F)C=C3)C=C1NC4=CC=C(C)C(C(F)(F)F)=C4</td>
<td>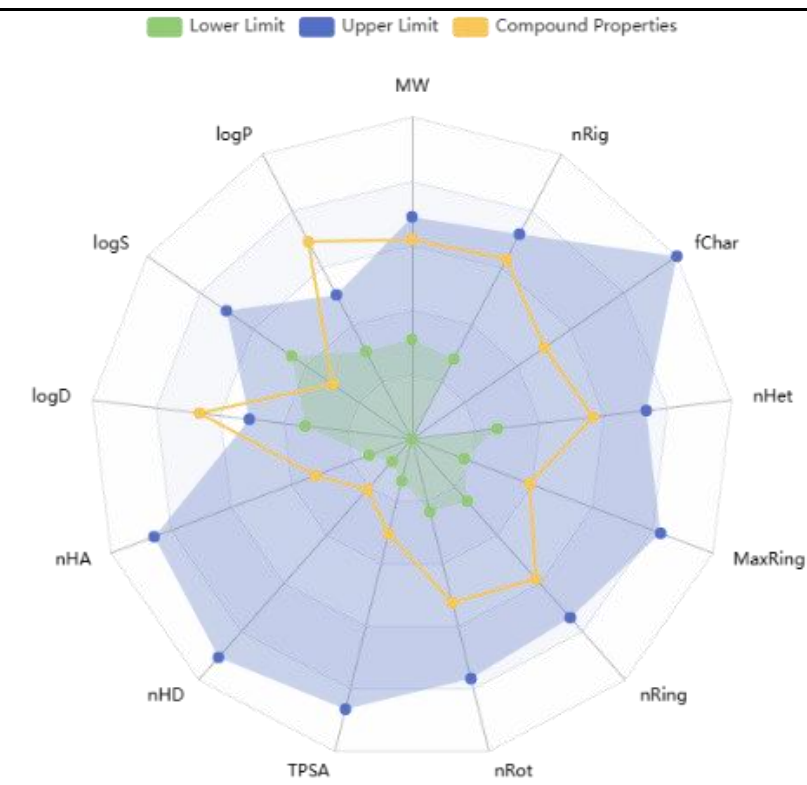
</td>
</tr>
<tr>
<td>(4o): O=C1N(C2=CC=C(C)C(C(F)(F)F)=C2)C(C3=CC=C(Cl)C=C3)C=C1NC4=CC=C(C)C(C(F)(F)F)=C4</td>
<td>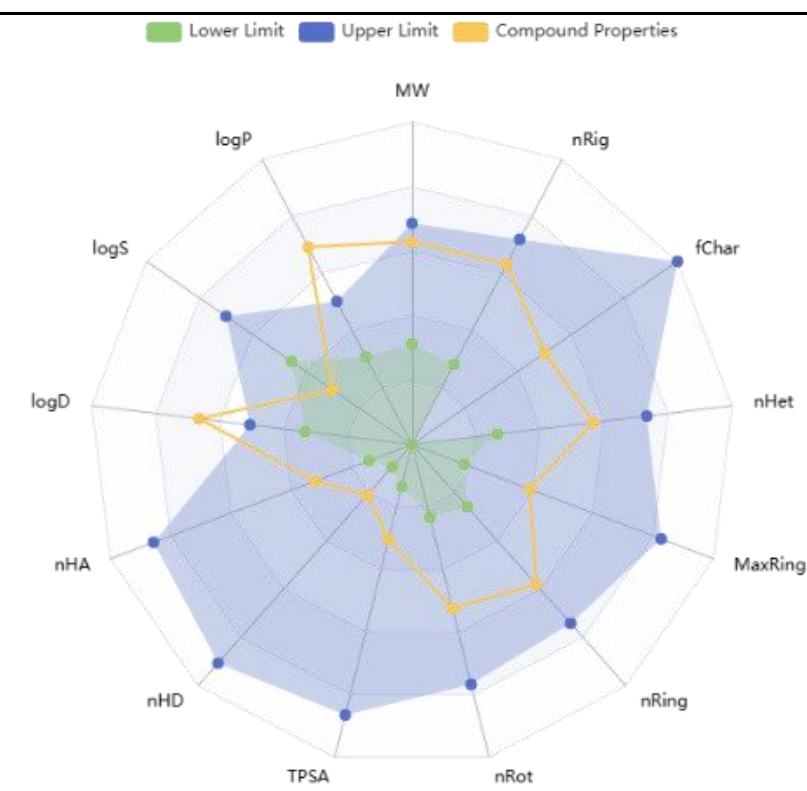
</td>
</tr>
<tr>
<td>(4p): O=C1N(C2=CC=C(C)C(C(F)(F)F)=C2)C(C3=CC=C(Br)C=C3)C=C1NC4=CC=C(C)C(C(F)(F)F)=C4</td>
<td>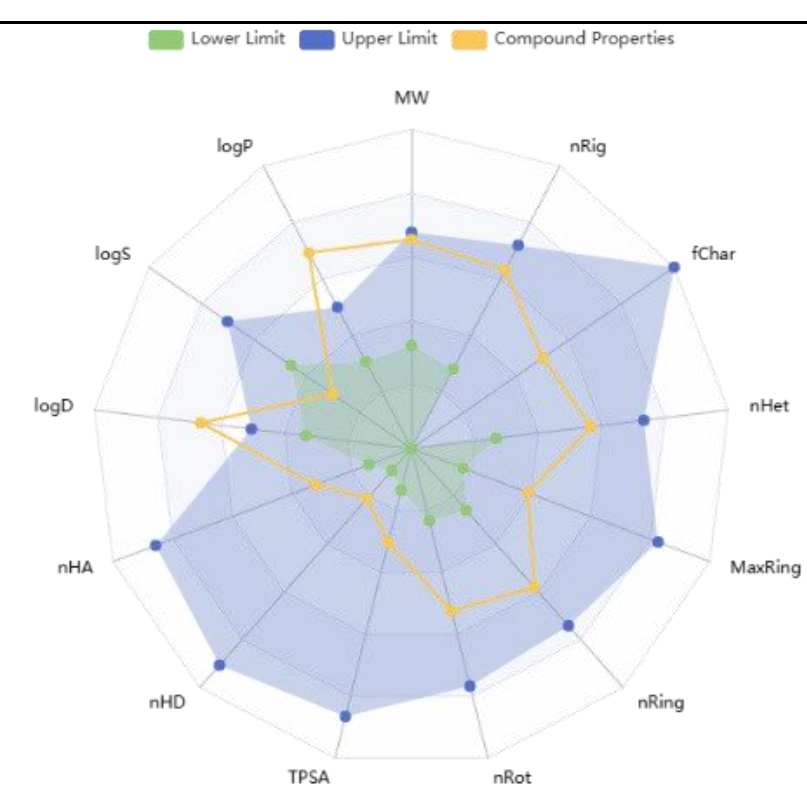
</td>
</tr>
</table>

**(4r):** O=C1N(C2=CC=C(C)C(C(F)(F)F)=C2)C(C3=CC=C(C(F)(F)F)C=C3)C=C1NC4=CC=C(C)C(C(F)(F)F)=C4	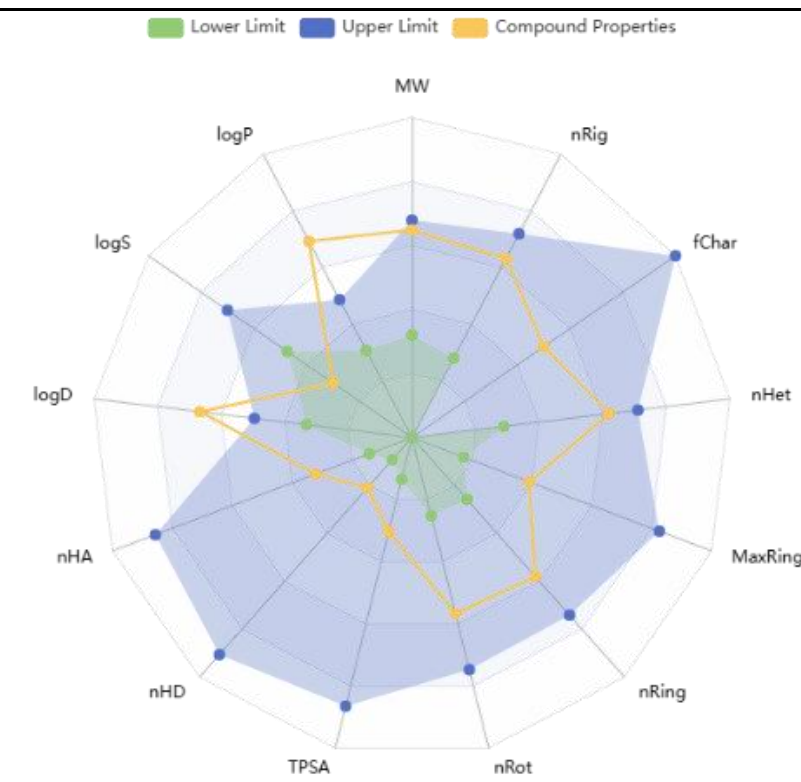
**(4s):** O=C1N(C2=CC=C(C)C(C(F)(F)F)=C2)C(C3=CC(C(F)(F)F)=CC(C(F)(F)F)=C3)C=C1NC4=CC=C(C)C(C(F)(F)F)=C4	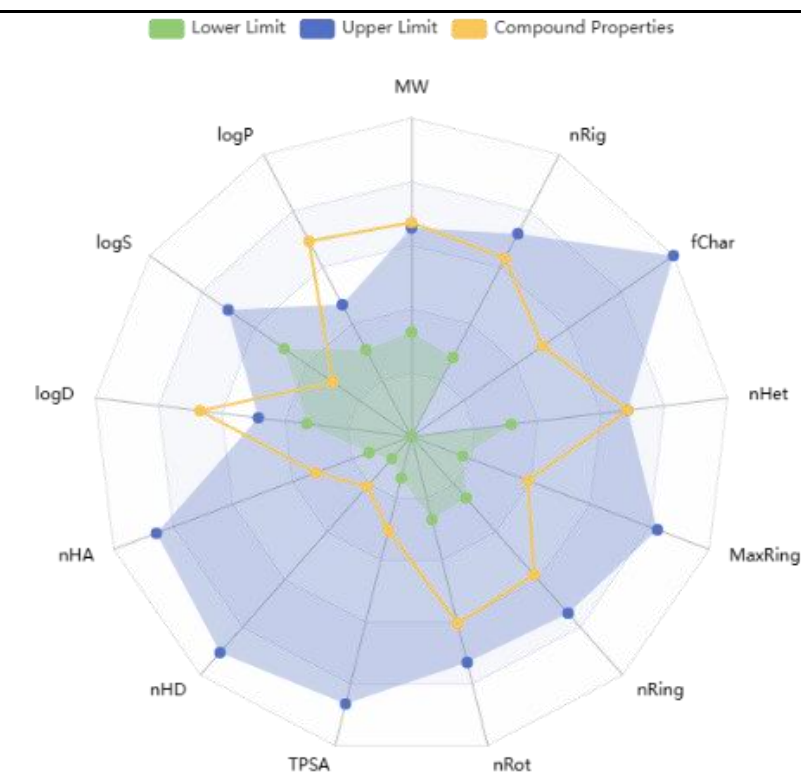
**(4t):** N#CC1=CC=C(C(C=C2NC3=CC=C(C)C(C(F)(F)F)=C3)N(C4=CC=C(C)C(C(F)(F)F)=C4)C2=O)C=C1	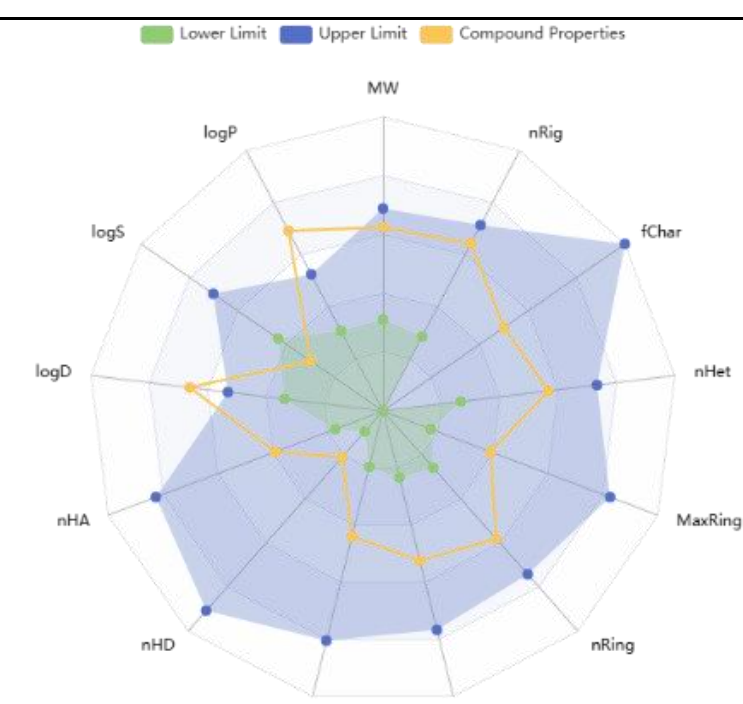

Table S2. Physicochemical Properties

Compound	MW (Da)	TPSA (Å²)	logP	logS
QC203	508.1	32.3	5.8	-6.8
QC204	524.1	32.3	5.9	-6.8
QC205	568.1	32.3	6.0	-7.0
QC206	558.1	32.3	6.5	-7.5
QC207	550.2	50.8	5.6	-6.8

QC208	550.2	50.8	5.2	-6.4
QC209	536.2	61.8	5.0	-5.6
QC210	566.2	71.0	4.9	-6.0
QC211	580.2	60.0	5.3	-6.7
QC212	580.2	60.0	5.4	-6.8
QC213	580.2	60.0	5.3	-6.7
QC214	580.2	60.0	5.4	-6.8
QC216	566.2	71.0	4.9	-6.0
QC217	558.1	32.3	6.5	-7.5
QC219	626.1	32.3	7.2	-7.9
QC222	536.2	61.8	5.0	-5.6
QC223	550.2	50.8	5.6	-6.8
QC224	550.2	50.8	5.2	-6.4
QC227	580.2	60.0	5.2	-6.3

Table S3. Hydrogen Bonding and Molecular Flexibility

Compound	nHA	nHD	nRot	Flexibility	nRing
QC203	3	1	6	0.3	4
QC204	3	1	6	0.3	4
QC205	3	1	6	0.3	4
QC206	3	1	7	0.3	4
QC207	5	2	7	0.3	4
QC208	5	1	8	0.3	4
QC209	5	2	7	0.3	4
QC210	6	2	8	0.3	4
QC211	6	1	9	0.4	4
QC212	6	1	9	0.4	4
QC213	6	1	9	0.4	4
QC214	6	1	9	0.4	4
QC216	6	2	8	0.3	4
QC217	3	1	7	0.3	4
QC219	3	1	8	0.3	4
QC222	5	2	7	0.3	4
QC223	5	1	8	0.3	4
QC224	5	1	8	0.3	4
QC227	6	1	9	0.4	4

Table S4. Absorption and Permeability

Compound	Caco-2 (log unit)	MDCK (log unit)	PAMPA
QC203	-4.8	-4.8	0.0
QC204	-4.8	-4.8	0.0
QC205	-4.9	-4.8	0.0
QC206	-4.9	-4.8	0.0
QC207	-4.7	-4.7	0.0
QC208	-4.7	-4.7	0.0

QC209	-4.8	-4.8	0.0
QC210	-5.0	-4.8	0.0
QC211	-4.8	-4.7	0.0
QC212	-4.8	-4.8	0.0
QC213	-4.8	-4.7	0.0
QC214	-4.7	-4.7	0.0
QC216	-5.0	-4.8	0.0
QC217	-4.9	-4.8	0.0
QC219	-4.9	-4.8	0.0
QC222	-4.8	-4.8	0.0
QC223	-4.7	-4.7	0.0
QC224	-4.7	-4.7	0.0
QC227	-4.8	-4.7	0.0

Table S5. Transporter Interaction

Compound	P-gp inhibitor	P-gp substrate
QC203	high	low
QC204	high	low
QC205	high	low
QC206	high	low
QC207	high	low
QC208	high	low
QC209	high	low
QC210	high	low
QC211	high	low
QC212	high	low
QC213	high	low
QC214	high	low
QC216	high	low
QC217	high	low
QC219	high	low
QC222	high	low
QC223	high	low
QC224	high	low
QC227	high	low

Table S6. Skin and Ocular Safety Predictions

Compound	Skin sensitivity	Skin permeability	Eye irritation
QC203	medium	medium	low
QC204	medium	medium	low
QC205	medium	medium	low
QC206	high	medium	medium
QC207	medium	medium	low
QC208	medium	medium	low
QC209	medium	low	low

QC210	low	low	low
QC211	medium	medium	low
QC212	medium	medium	low
QC213	medium	medium	low
QC214	medium	medium	low
QC216	low	low	low
QC217	high	high	medium
QC219	high	high	medium
QC222	low	low	low
QC223	medium	medium	low
QC224	medium	medium	low
QC227	medium	medium	low

Table S7. Toxicological Alerts

Compound	Acute dermal toxicity	ALARM NMR alerts	PAINS alerts
QC203	medium	medium	low
QC204	medium	medium	low
QC205	medium	medium	low
QC206	high	medium	low
QC207	medium	medium	low
QC208	medium	medium	low
QC209	medium	low	low
QC210	low	low	low
QC211	medium	medium	low
QC212	medium	medium	low
QC213	medium	medium	low
QC214	medium	medium	low
QC216	low	low	low
QC217	high	high	medium
QC219	high	high	medium
QC222	low	low	low
QC223	medium	medium	low
QC224	medium	medium	low
QC227	medium	medium	low

Table S8. Medicinal Chemistry Descriptors

Compound	Synthetic accessibility score	Fsp3	MCE-18
QC203	3.0	0.3	94.8
QC204	3.0	0.3	94.8
QC205	3.1	0.3	95.2
QC206	3.1	0.3	95.2
QC207	3.0	0.3	94.8
QC208	3.0	0.3	94.8

QC209	3.0	0.3	95.0
QC210	3.1	0.3	95.6
QC211	3.0	0.4	94.8
QC212	3.0	0.4	94.8
QC213	3.0	0.4	94.8
QC214	3.0	0.4	94.8
QC216	3.1	0.3	95.6
QC217	3.1	0.3	95.2
QC219	3.2	0.3	96.1
QC222	3.0	0.3	95.0
QC223	3.0	0.3	94.8
QC224	3.0	0.3	94.8
QC227	3.0	0.4	94.8

Table S9. Drug-likeness and Developability

Compound	QED	Lipinski compliance	Pfizer rule	Golden Triangle
QC203	0.3	medium	medium	medium
QC204	0.3	medium	medium	medium
QC205	0.3	medium	medium	medium
QC206	0.3	low	medium	medium
QC207	0.3	medium	medium	medium
QC208	0.3	medium	medium	medium
QC209	0.3	medium	medium	high
QC210	0.3	high	high	high
QC211	0.3	medium	medium	medium
QC212	0.3	medium	medium	medium
QC213	0.3	medium	medium	medium
QC214	0.3	medium	medium	medium
QC216	0.3	high	high	high
QC217	0.3	low	low	low
QC219	0.3	low	low	low
QC222	0.3	high	high	high
QC223	0.3	medium	medium	medium
QC224	0.3	medium	medium	medium
QC227	0.3	medium	medium	medium

Table S10. Experimental Reliability Risks

Compound	Colloidal aggregation risk	Reactive compounds	Promiscuity score
QC203	medium	low	low
QC204	medium	low	low
QC205	medium	low	low
QC206	medium	medium	low
QC207	medium	low	low

QC208	medium	low	low
QC209	low	low	low
QC210	low	low	low
QC211	medium	low	low
QC212	medium	low	low
QC213	medium	low	low
QC214	medium	low	low
QC216	low	low	low
QC217	high	medium	medium
QC219	high	medium	medium
QC222	low	low	low
QC223	medium	low	low
QC224	medium	low	low
QC227	medium	low	low

Table S11. Antimicrobial-Relevant Summary

Compound	logP/TPSA balance	Permeability profile	Overall ADMET suitability
QC203	medium	medium	medium
QC204	medium	medium	medium
QC205	medium	medium	medium
QC206	low	medium	low
QC207	medium	medium	medium
QC208	medium	medium	medium
QC209	medium	high	high
QC210	high	high	high
QC211	medium	medium	medium
QC212	medium	medium	medium
QC213	medium	medium	medium
QC214	medium	medium	medium
QC216	high	high	high
QC217	low	medium	low
QC219	low	medium	low
QC222	high	high	high
QC223	medium	medium	medium
QC224	medium	medium	medium
QC227	medium	medium	medium

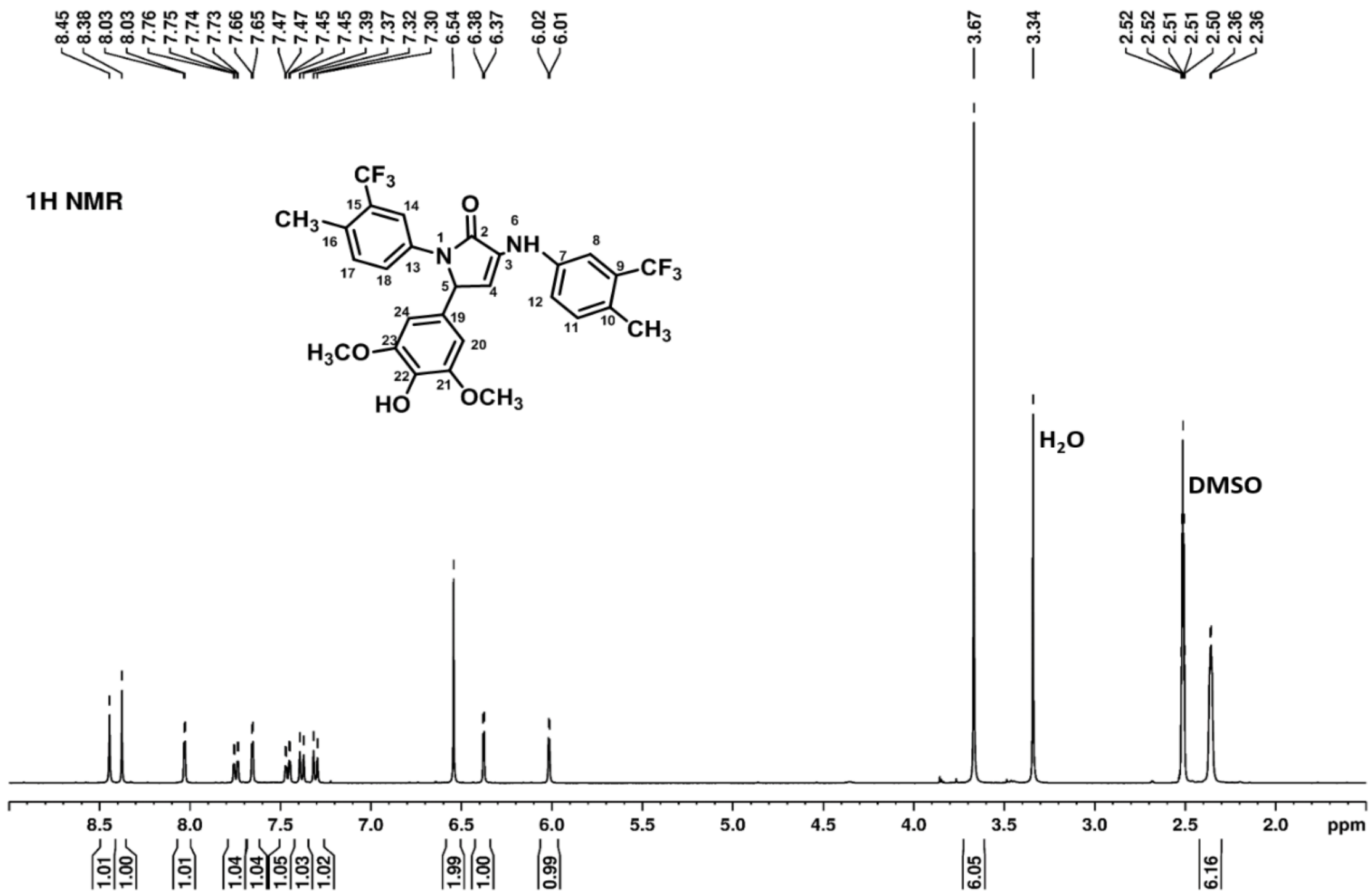

Figure 31S. ^{1}H-NMR spectrum **4a** recorded in DMSO-d6, at 400.1 MHz.

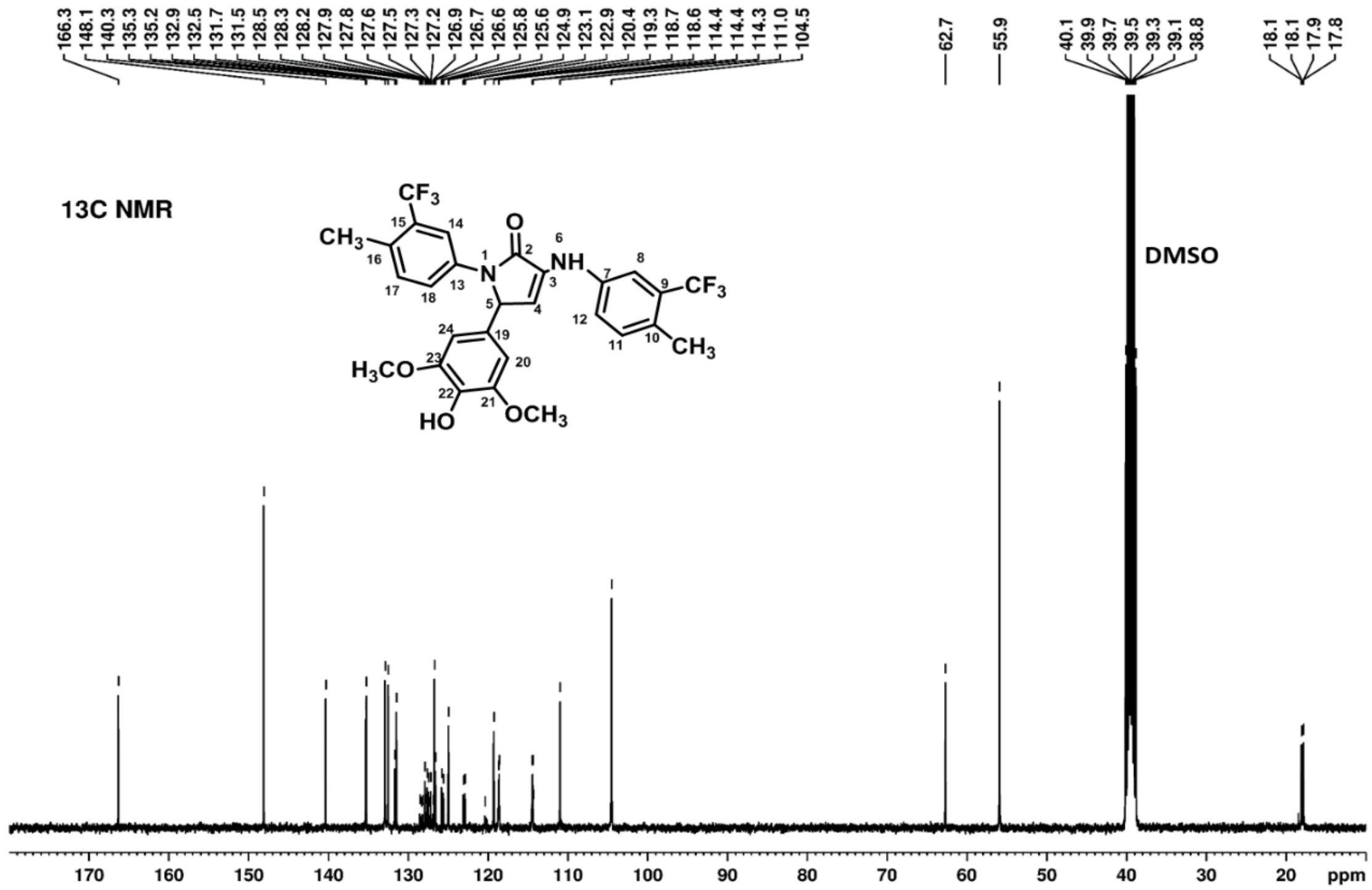

Figure 32S. ^{13}C-NMR spectrum **4a** recorded in DMSO-d_6, at 100.6 MHz.

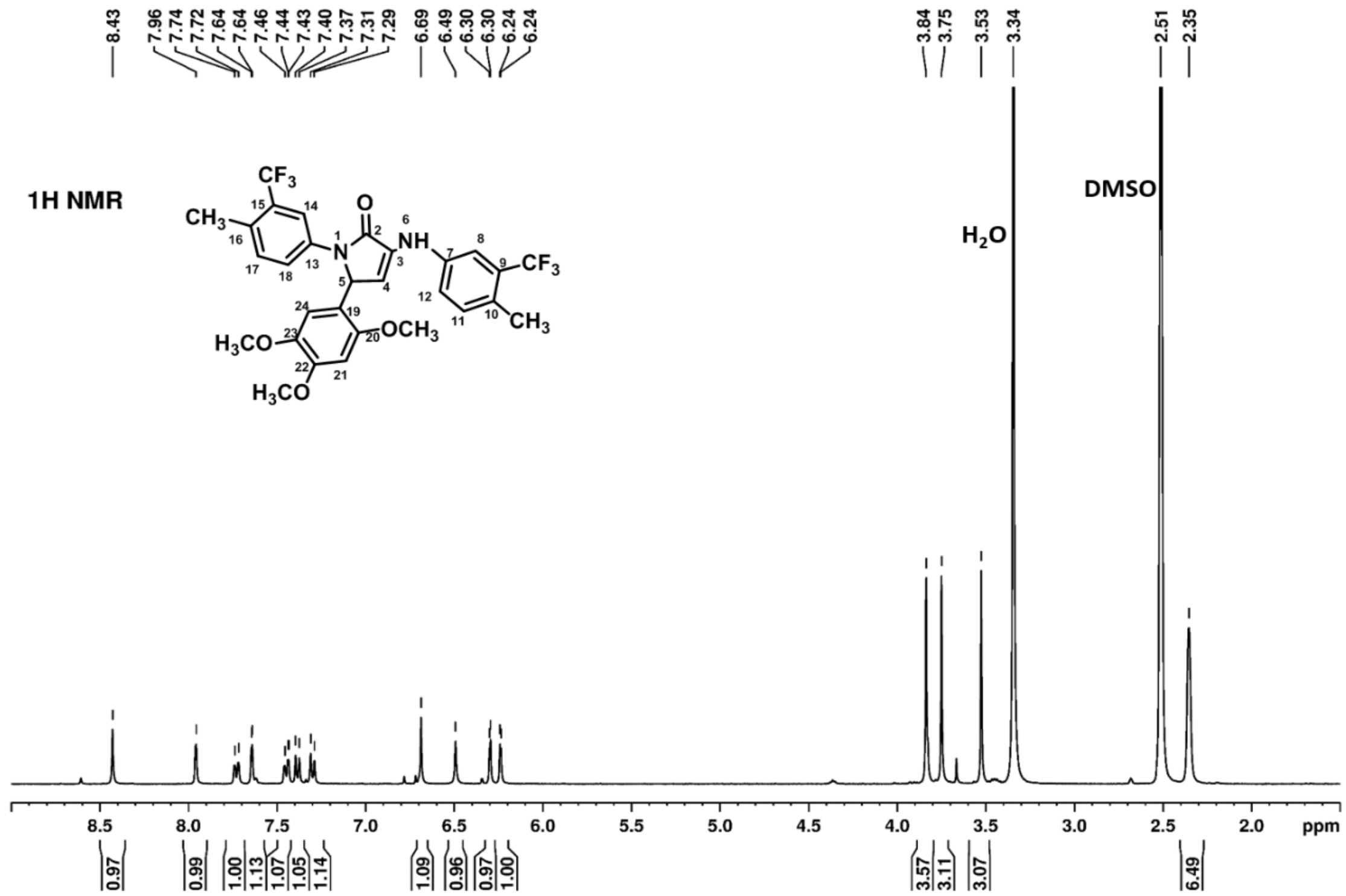

Figure 33S. ^{1}H-NMR spectrum **4c** recorded in DMSO-d6, at 400.1 MHz.

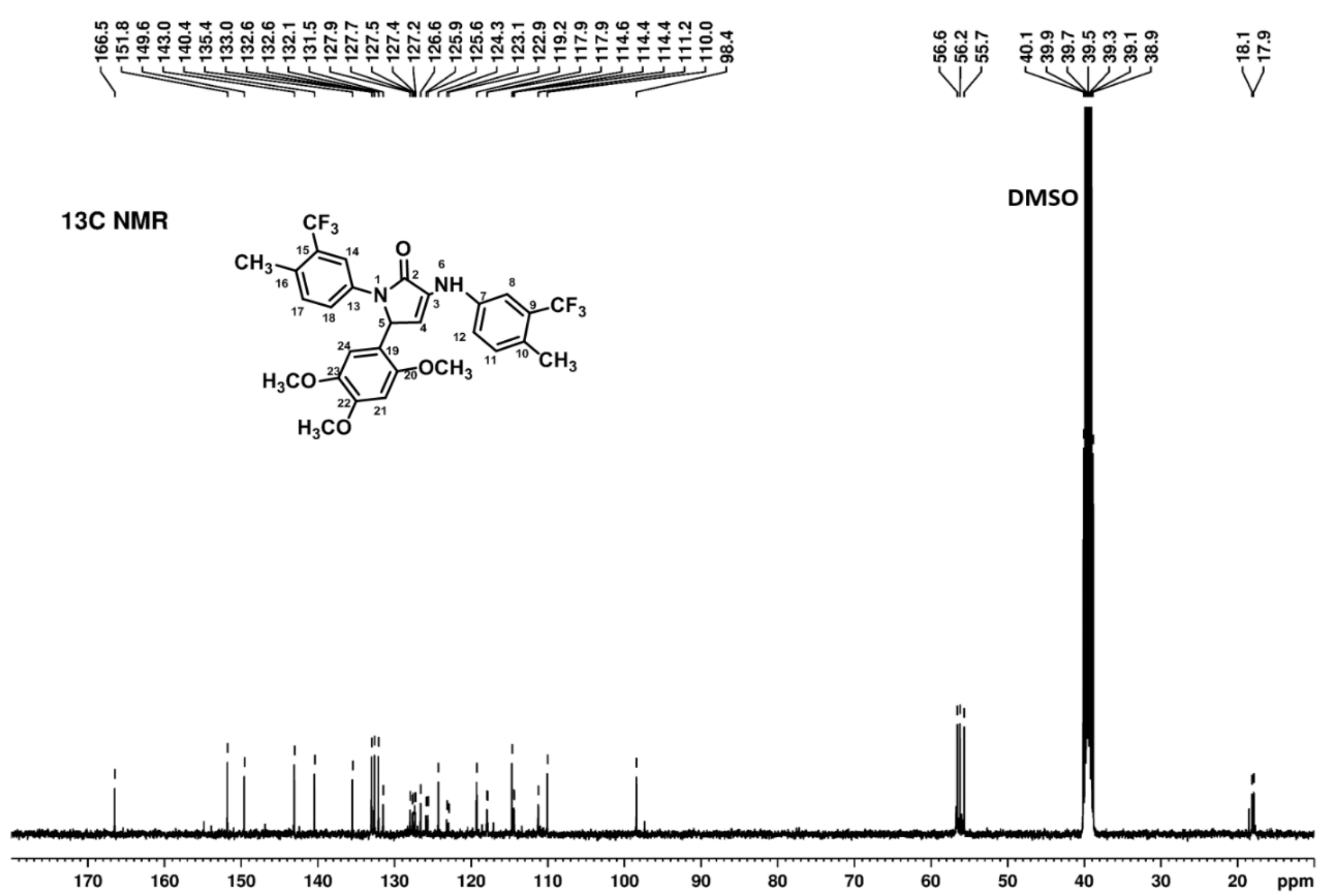

Figure 34S. ^{13}C-NMR spectrum **4c** recorded in DMSO-d_6, at 100.6 MHz.

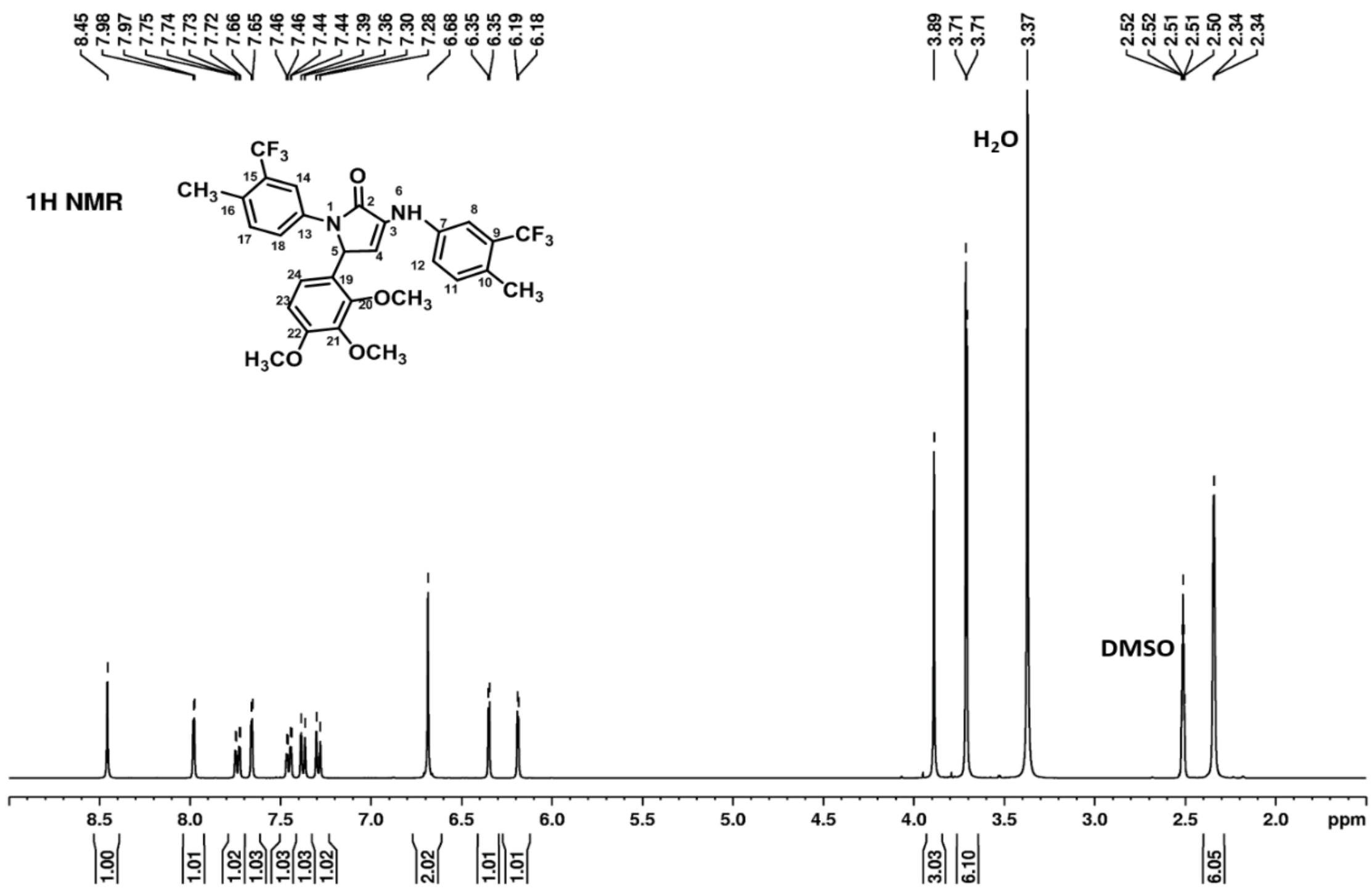

Figure 35S. ^{1}H-NMR spectrum **4d** recorded in DMSO-d6, at 400.1 MHz.

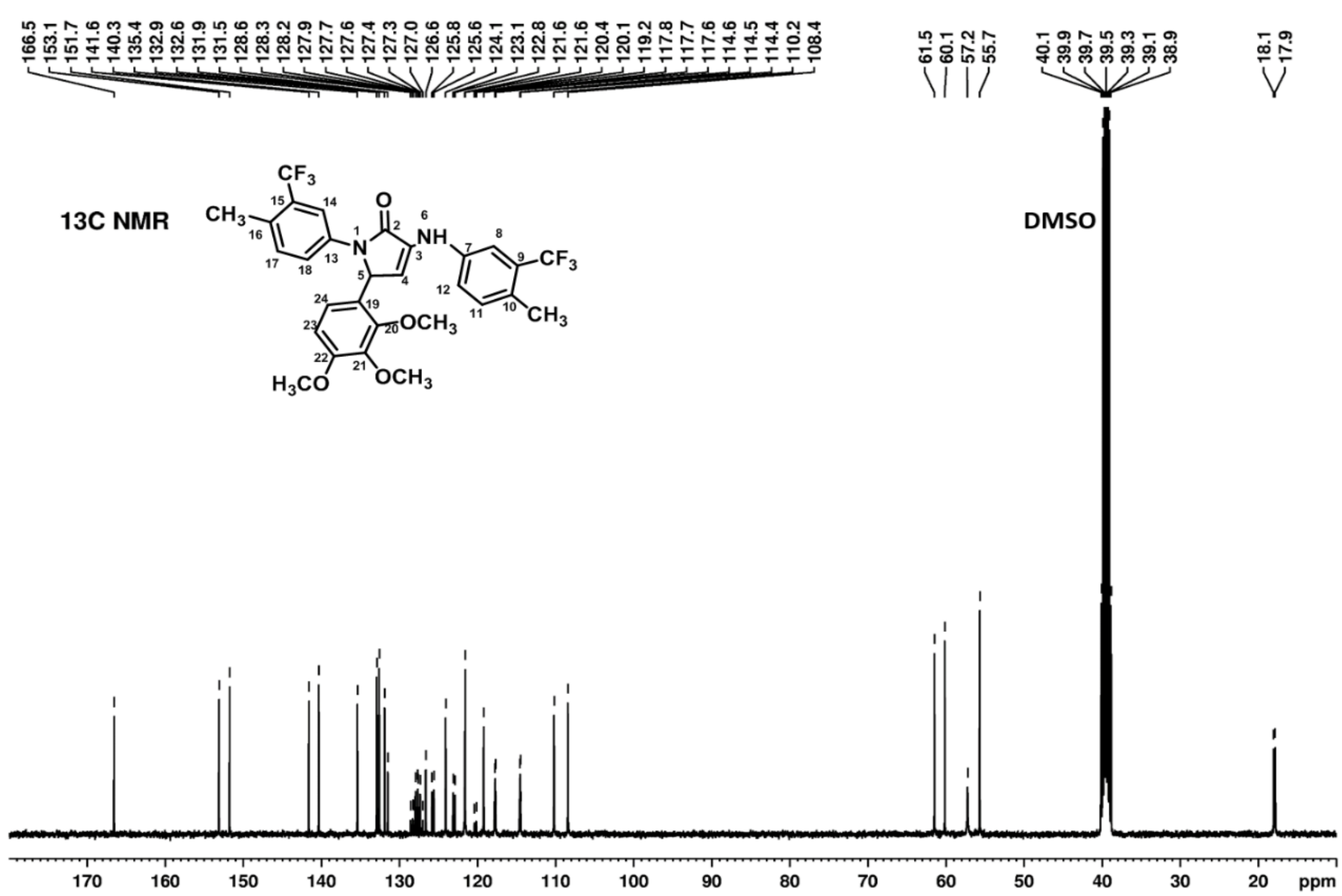

Figure 36S. ^{13}C-NMR spectrum **4d** recorded in DMSO-d_6, at 100.6 MHz.

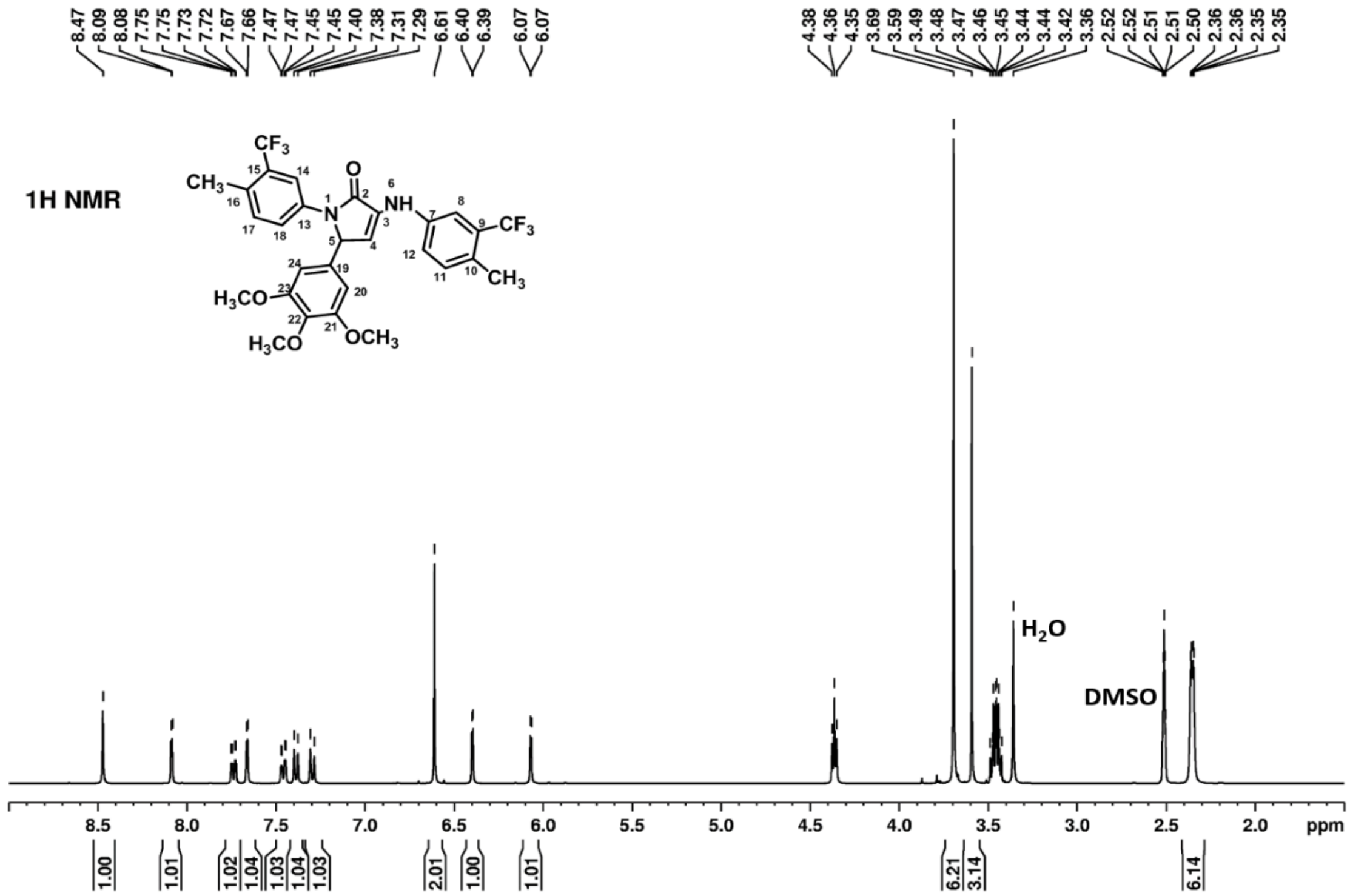

Figure 37S. ^{1}H-NMR spectrum **4e** recorded in DMSO-d6, at 400.1 MHz.

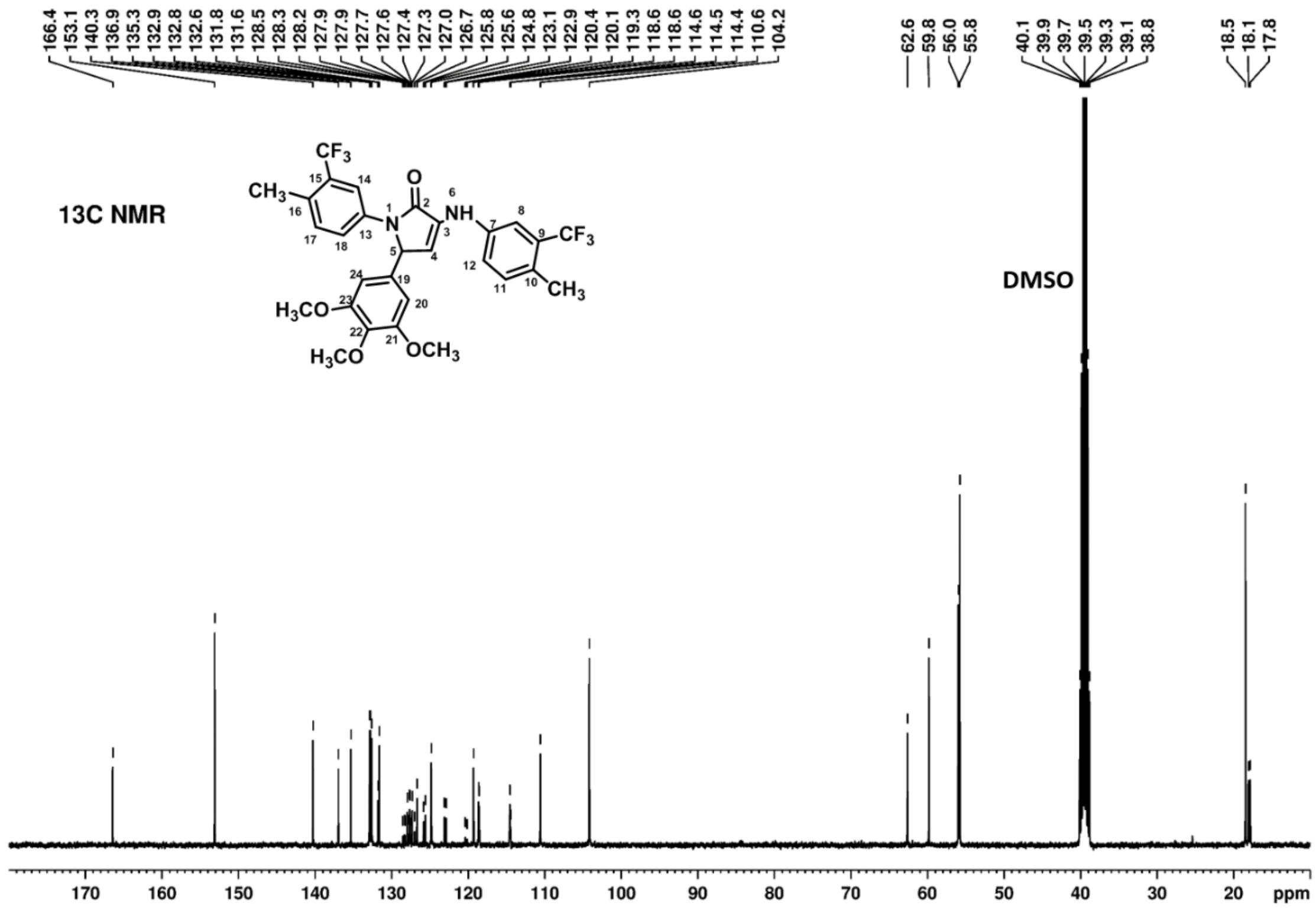

Figure 38S. ^{13}C-NMR spectrum **4e** recorded in DMSO-d_6, at 100.6 MHz.

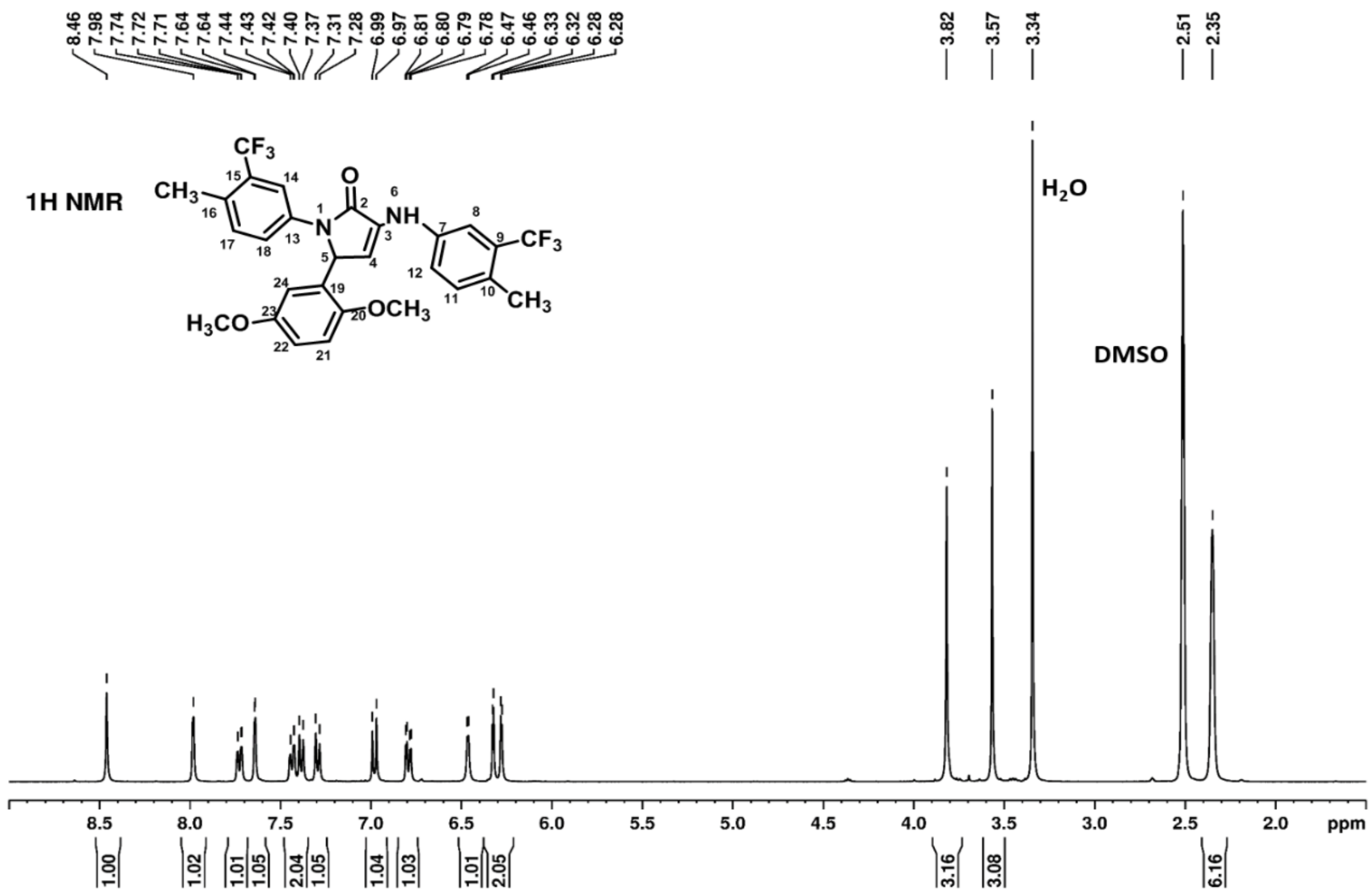

Figure 39S. ^{1}H-NMR spectrum **4f** recorded in DMSO-d6, at 400.1 MHz.

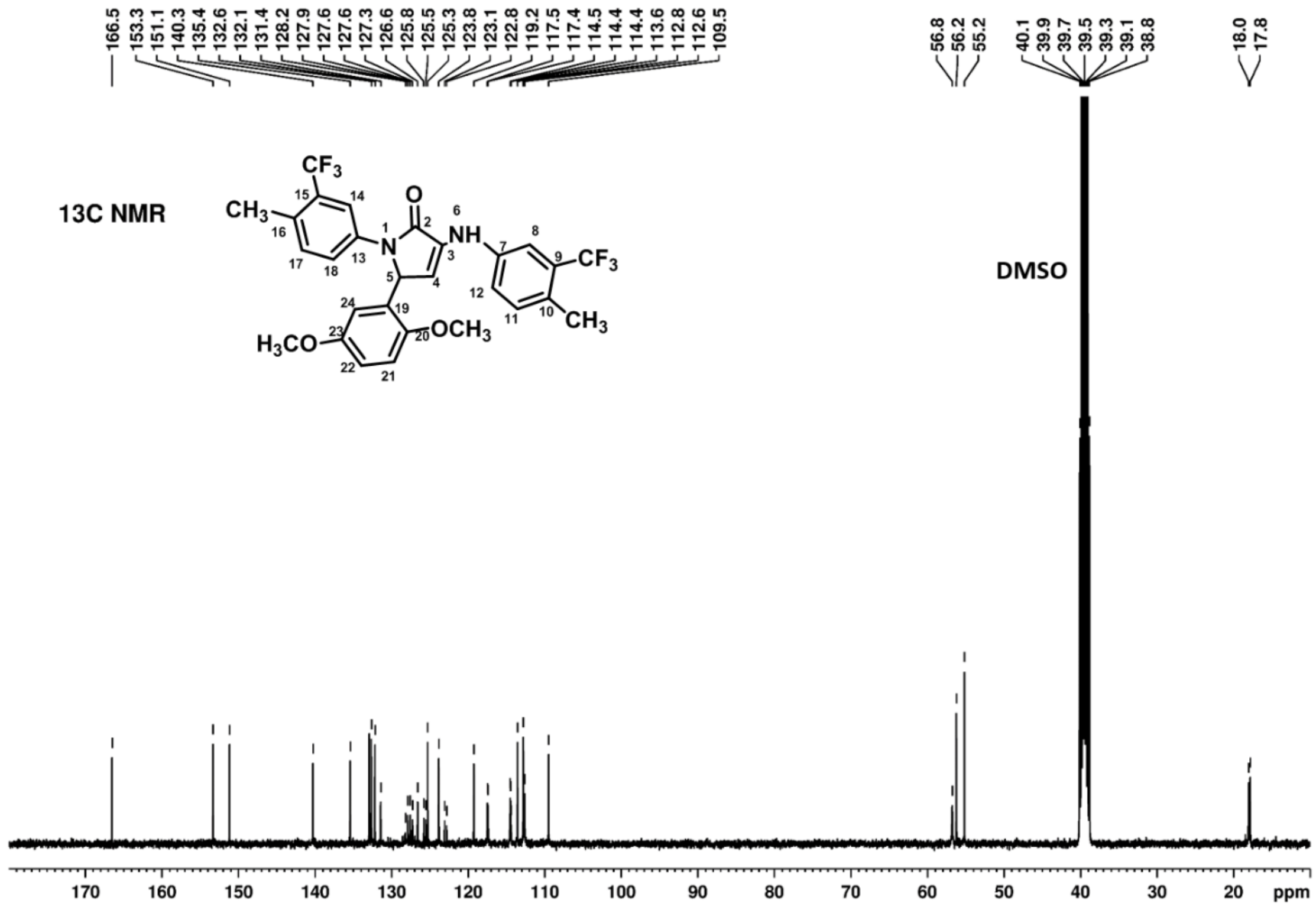

Figure 40S. ^{13}C-NMR spectrum **4f** recorded in DMSO-d_6, at 100.6 MHz.

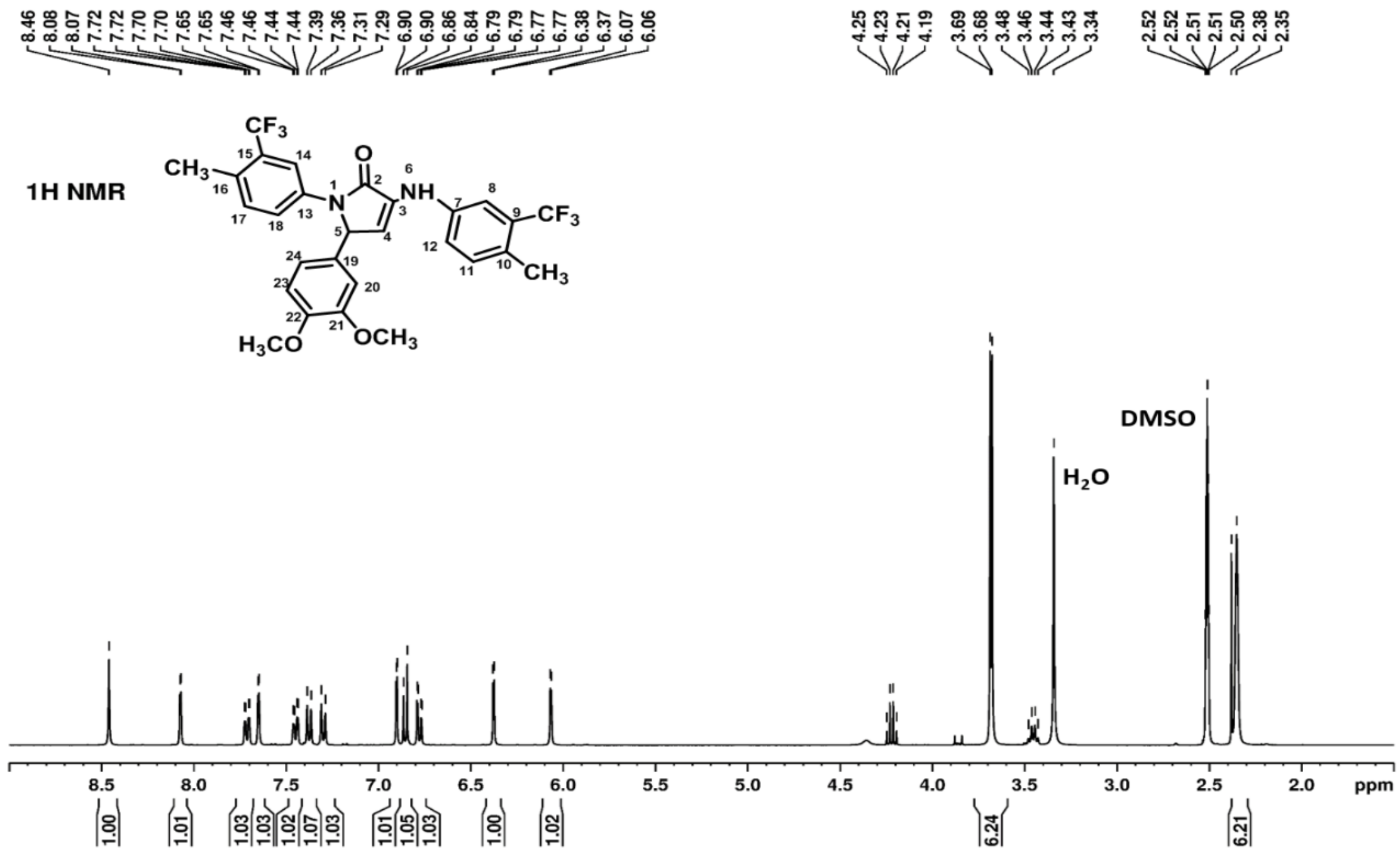

Figure 41S. ^{1}H-NMR spectrum **4g** recorded in DMSO-d6, at 400.1 MHz.

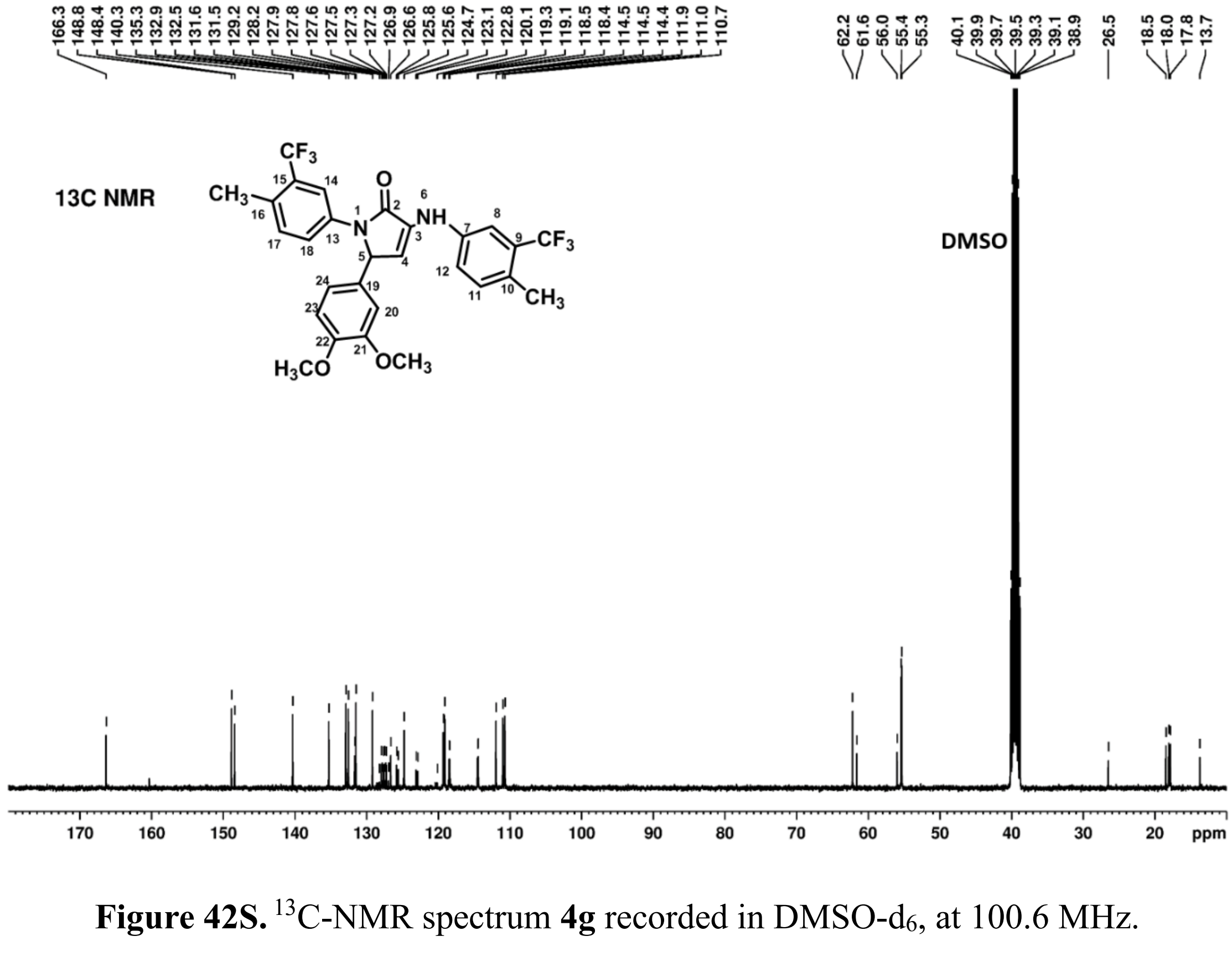

Figure 42S. ^{13}C-NMR spectrum **4g** recorded in DMSO-d_6, at 100.6 MHz.

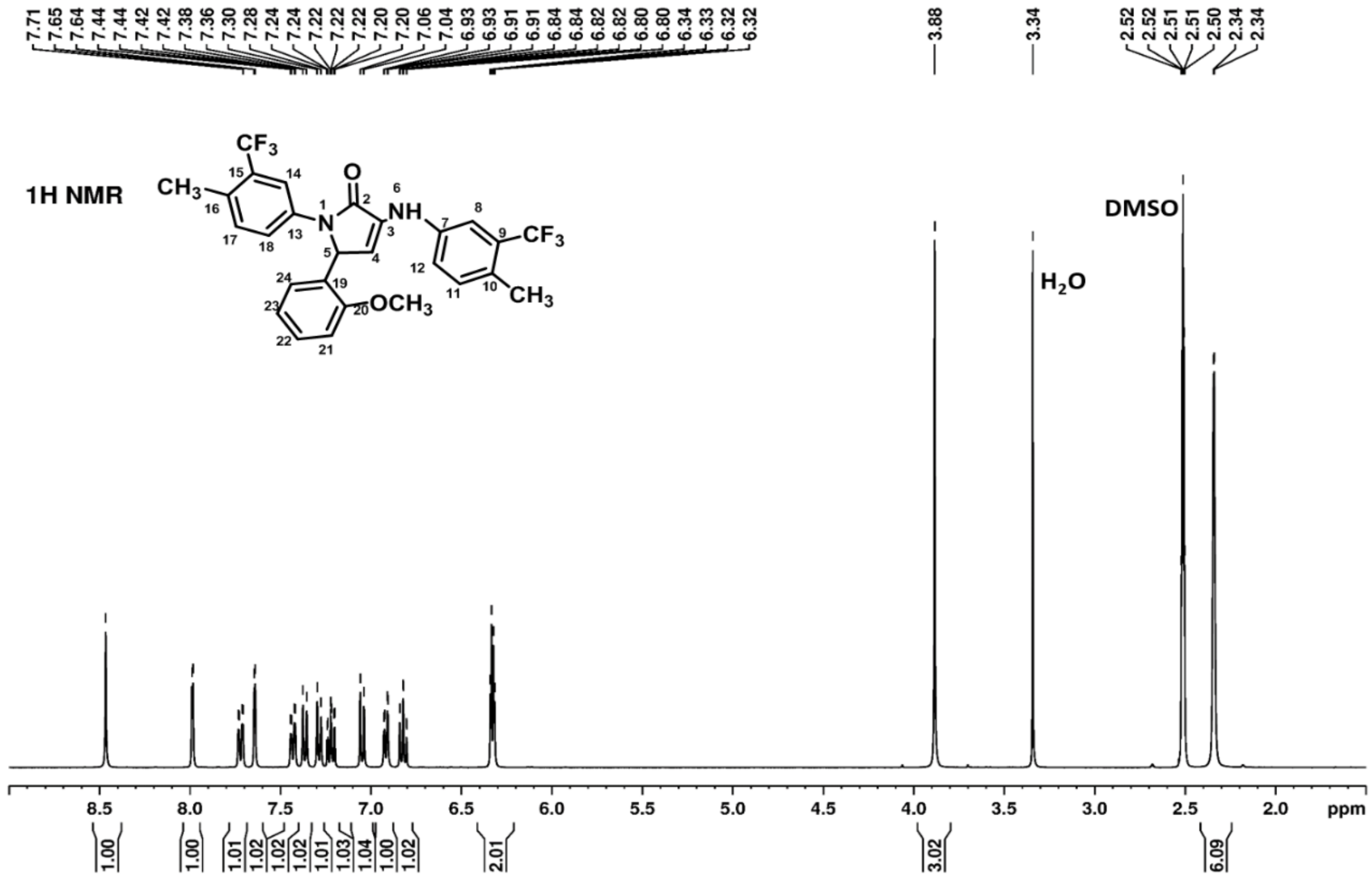

Figure 43S. ^{1}H-NMR spectrum **4h** recorded in DMSO-d6, at 400.1 MHz.

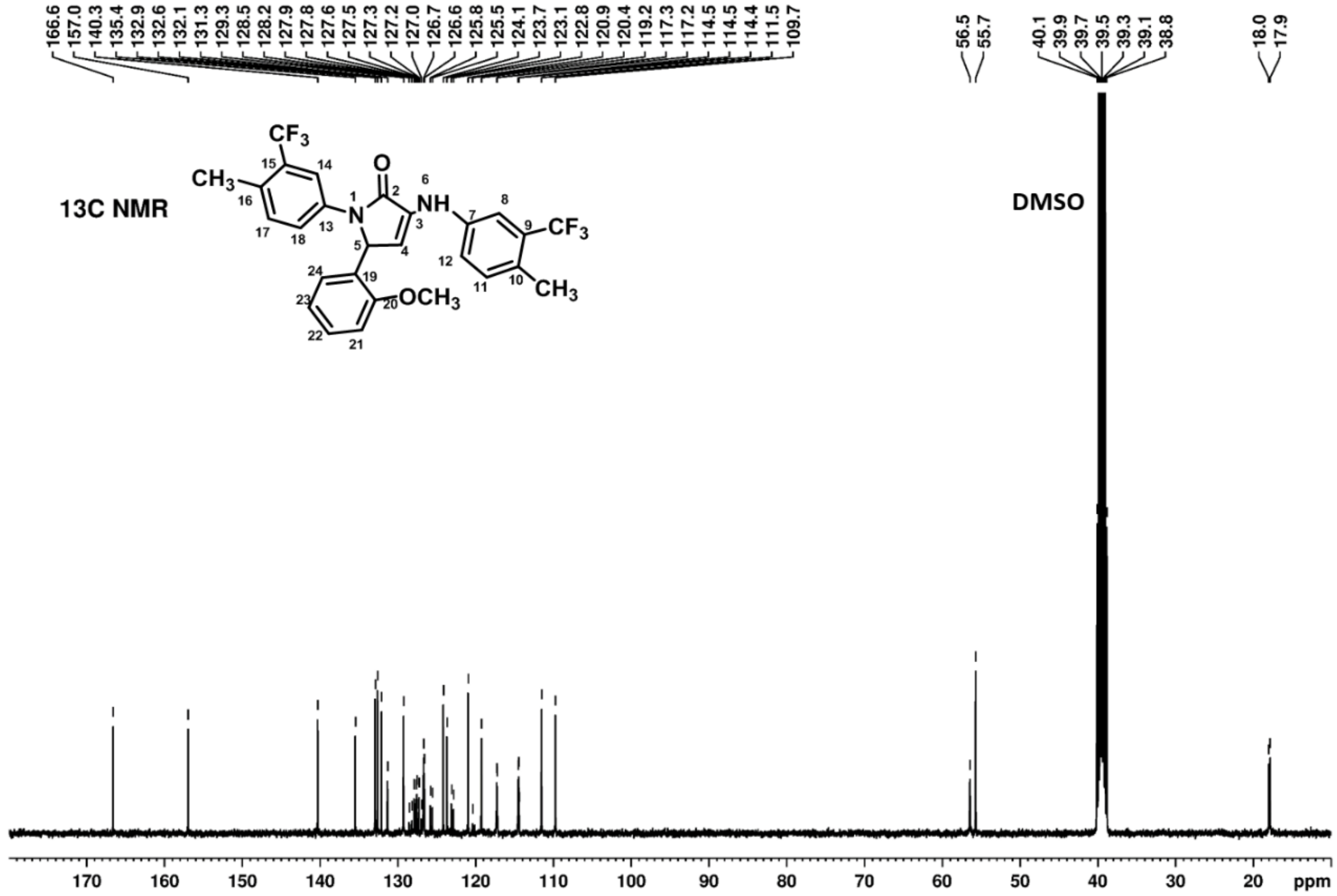

Figure 44S. ^{13}C-NMR spectrum **4h** recorded in DMSO-d_6, at 100.6 MHz.

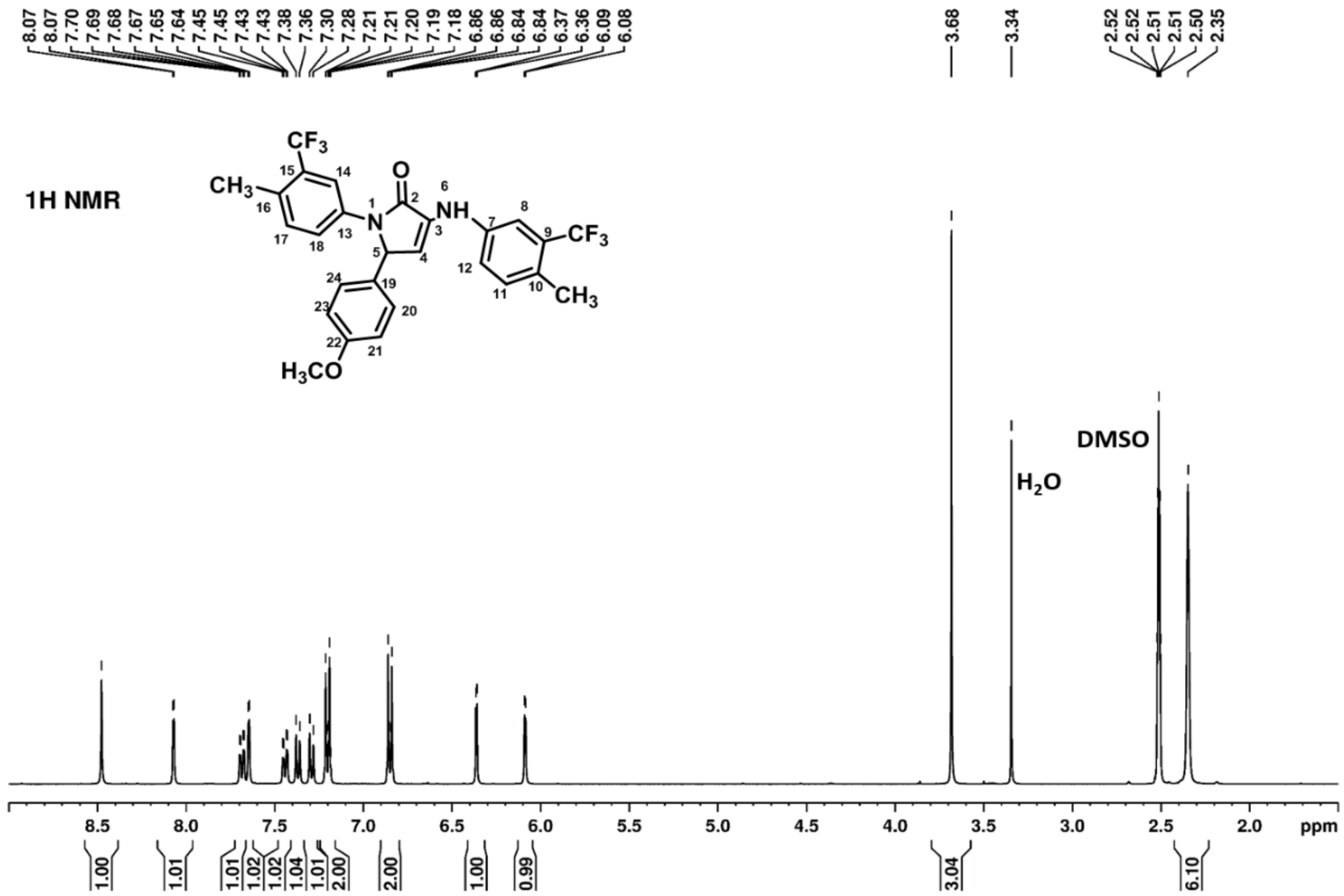

Figure 45S. ^{1}H-NMR spectrum **4i** recorded in DMSO-d6, at 400.1 MHz.

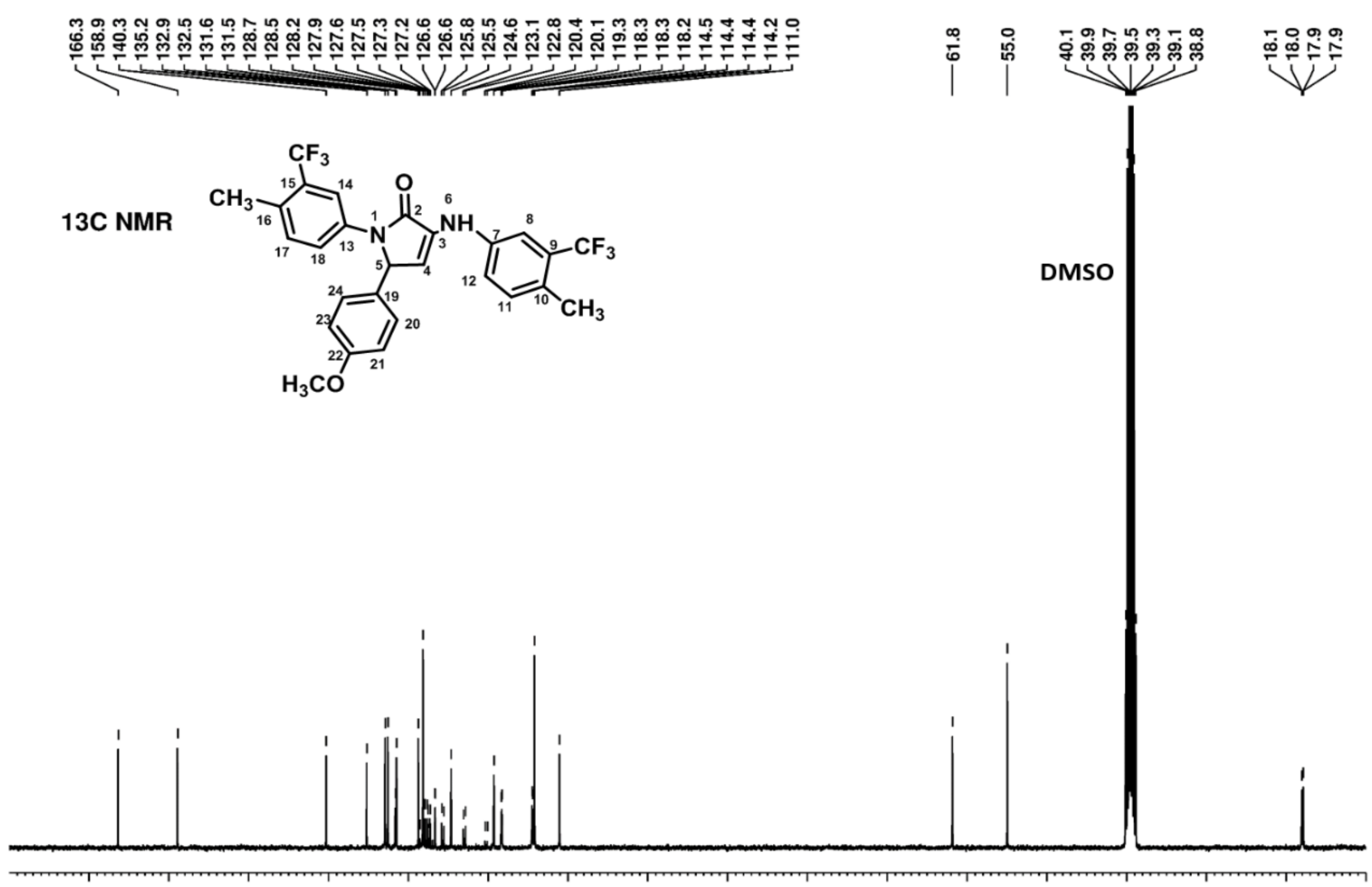

Figure 46S. ^{13}C-NMR spectrum **4i** recorded in DMSO-d_6, at 100.6 MHz.

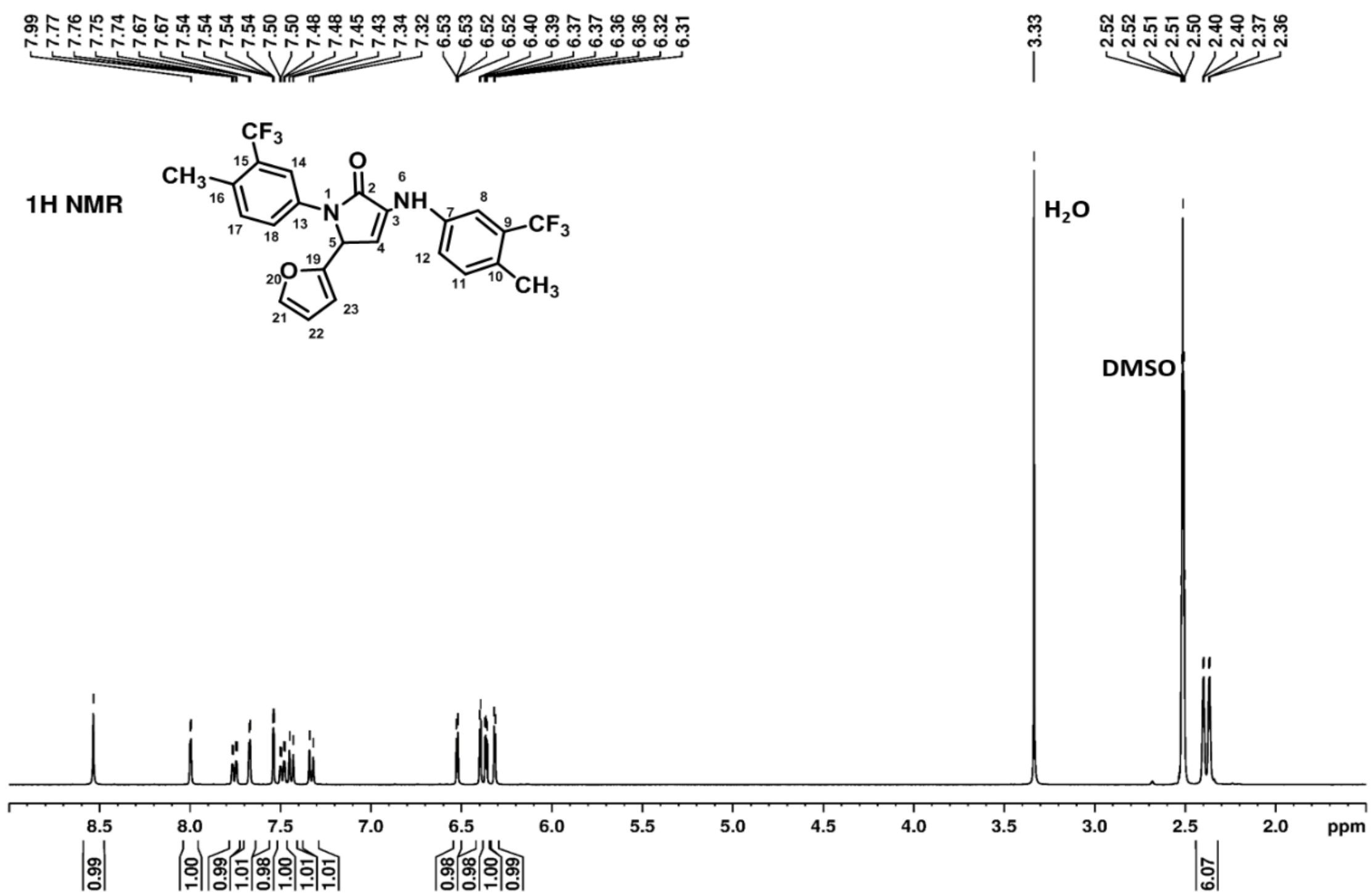

Figure 47S. ^{1}H-NMR spectrum **4j** recorded in DMSO-d6, at 400.1 MHz.

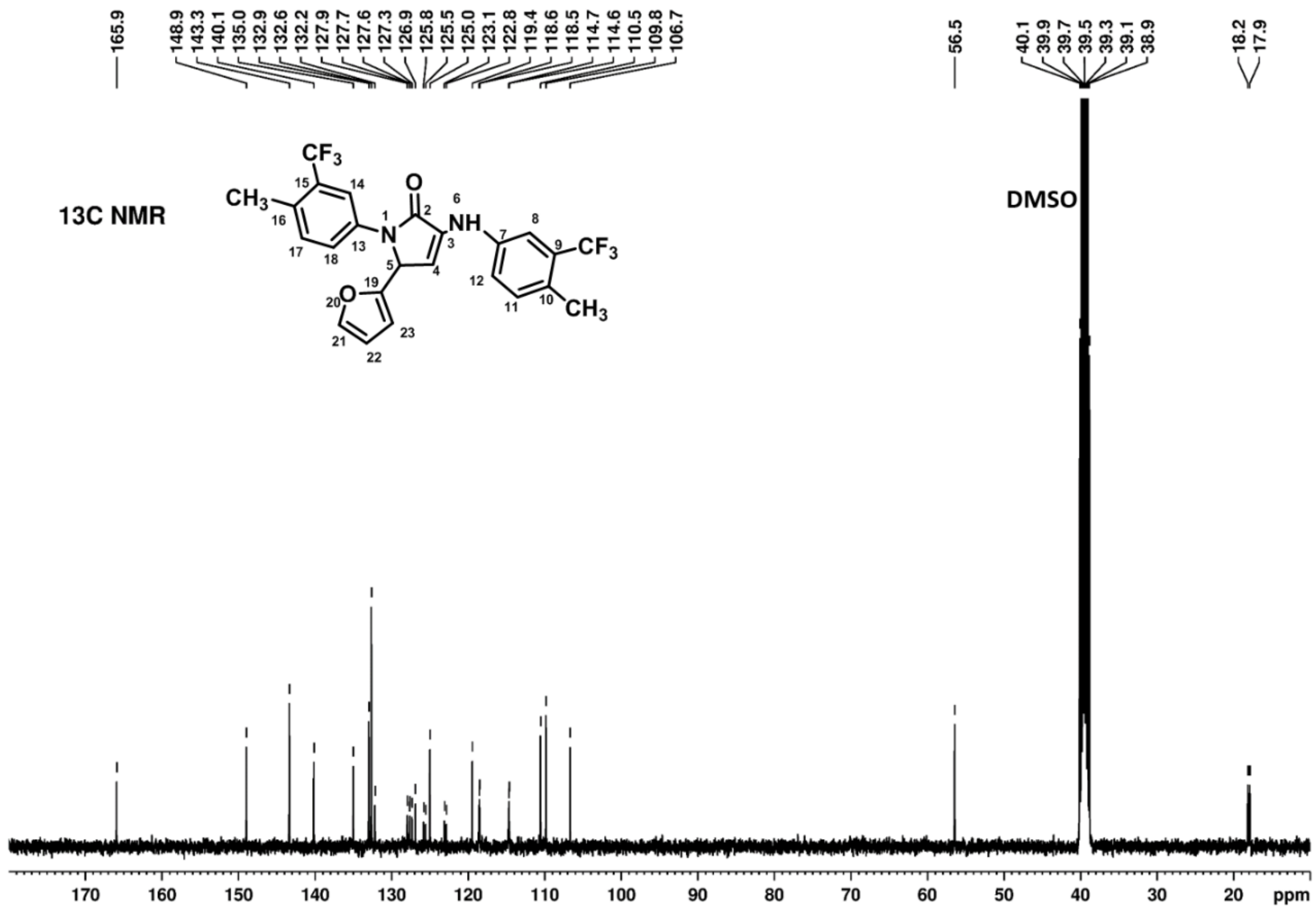

Figure 48S. ^{13}C-NMR spectrum **4j** recorded in DMSO-d_6, at 100.6 MHz.

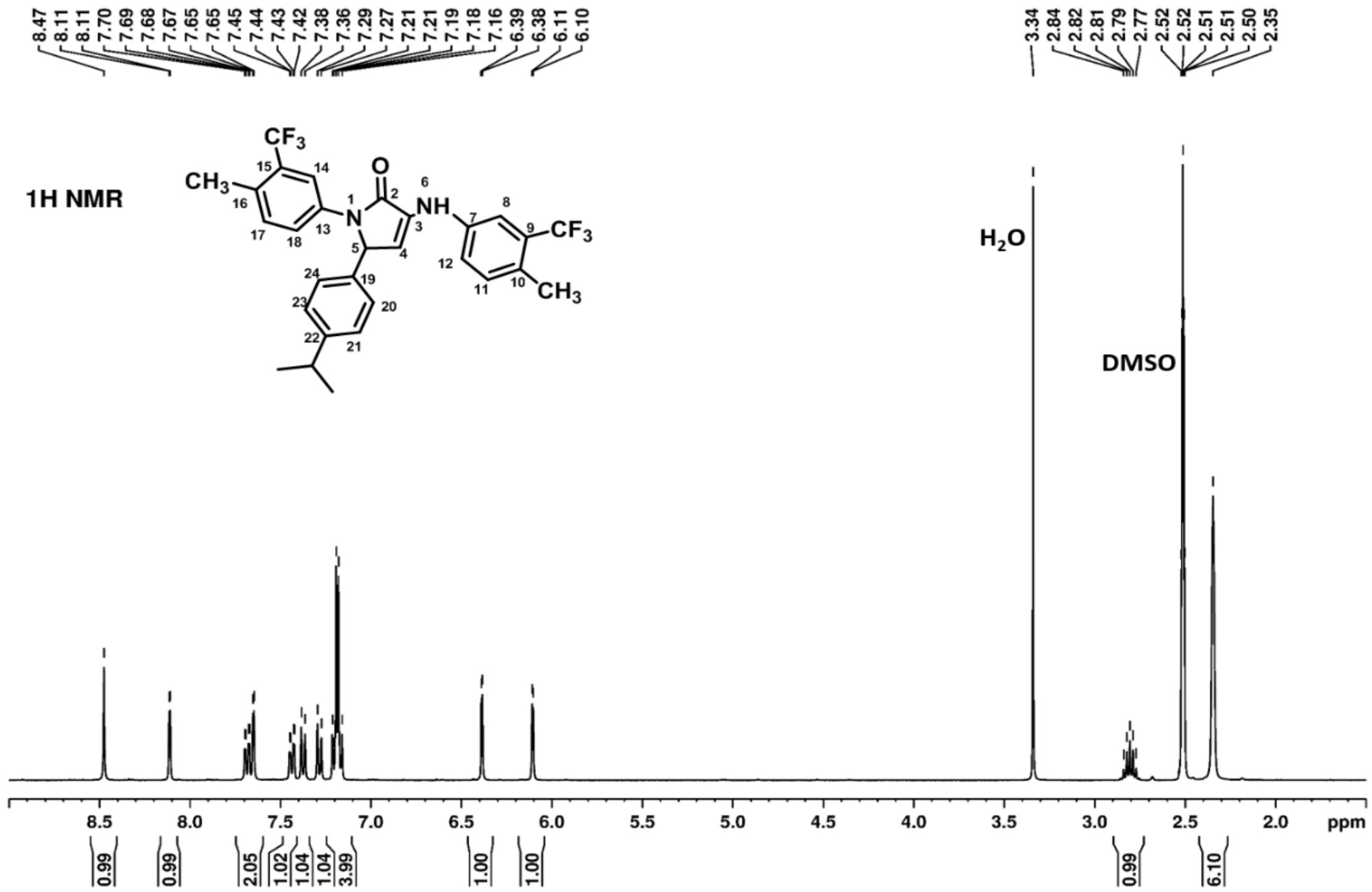

Figure 49S. ^{1}H-NMR spectrum **4k** recorded in DMSO-d6, at 400.1 MHz.

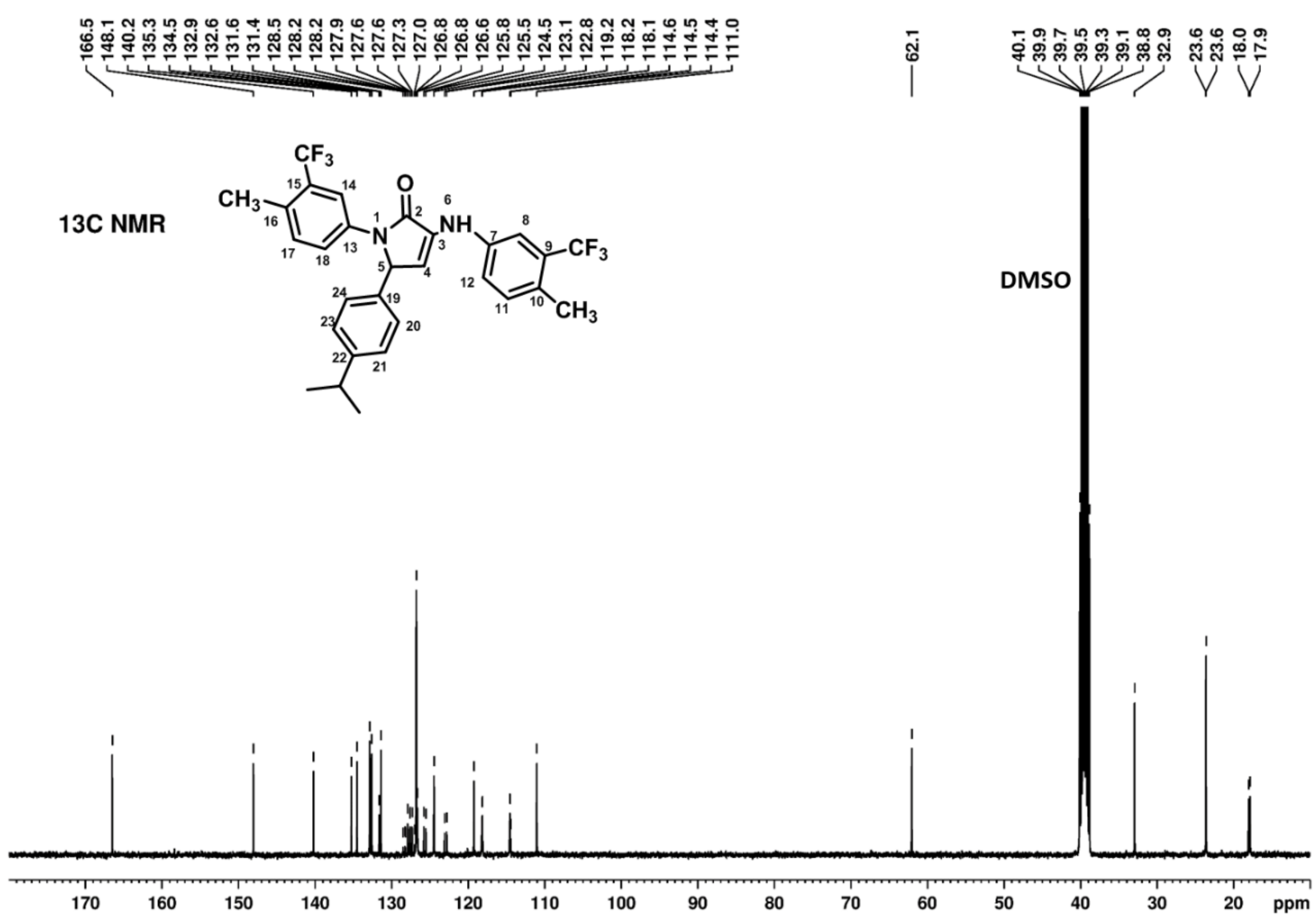

Figure 50S. ^{13}C-NMR spectrum **4k** recorded in DMSO-d$_6$, at 100.6 MHz.

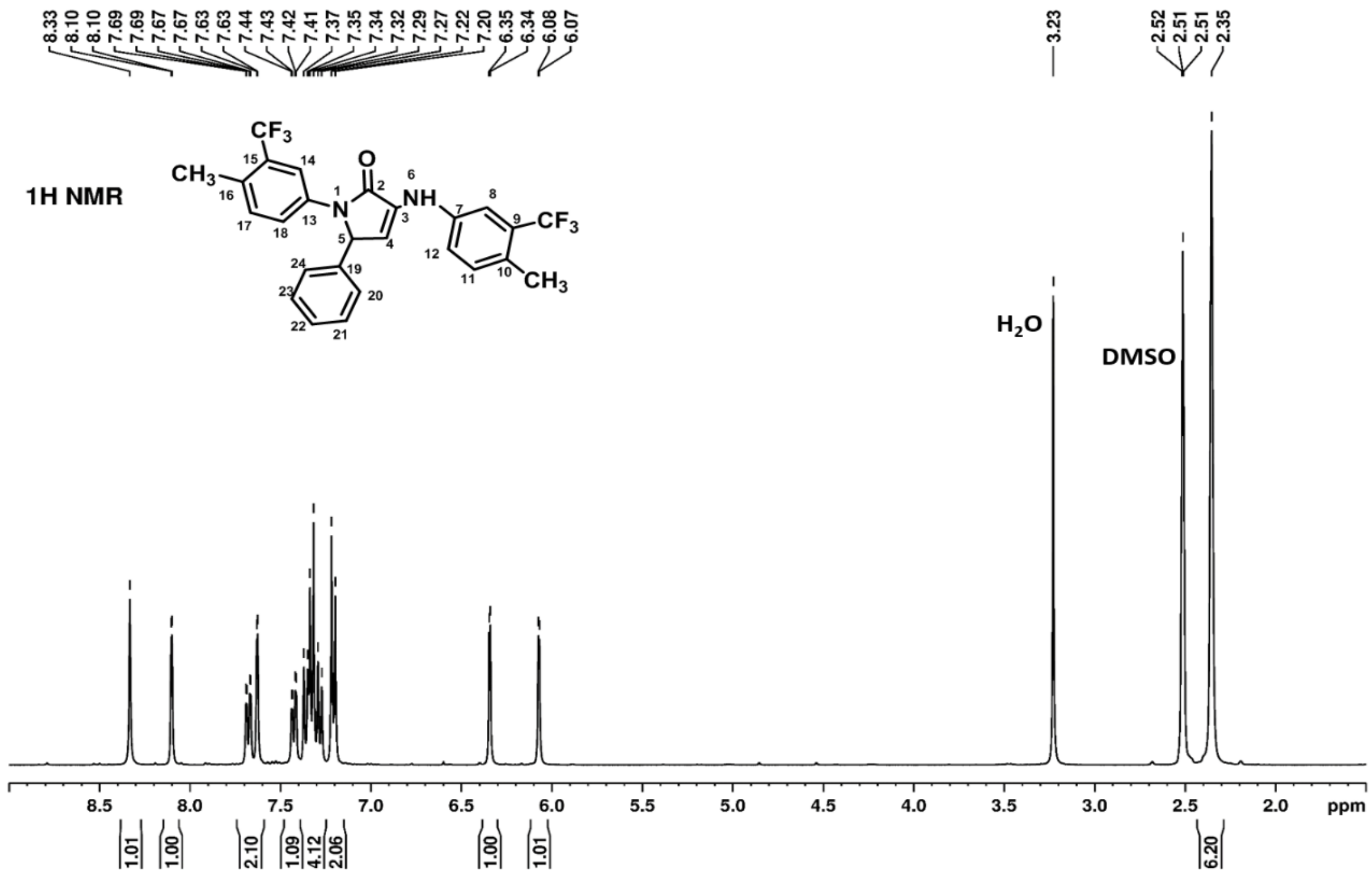

Figure 51S. ^{1}H-NMR spectrum **4m** recorded in DMSO-d6, at 400.1 MHz.

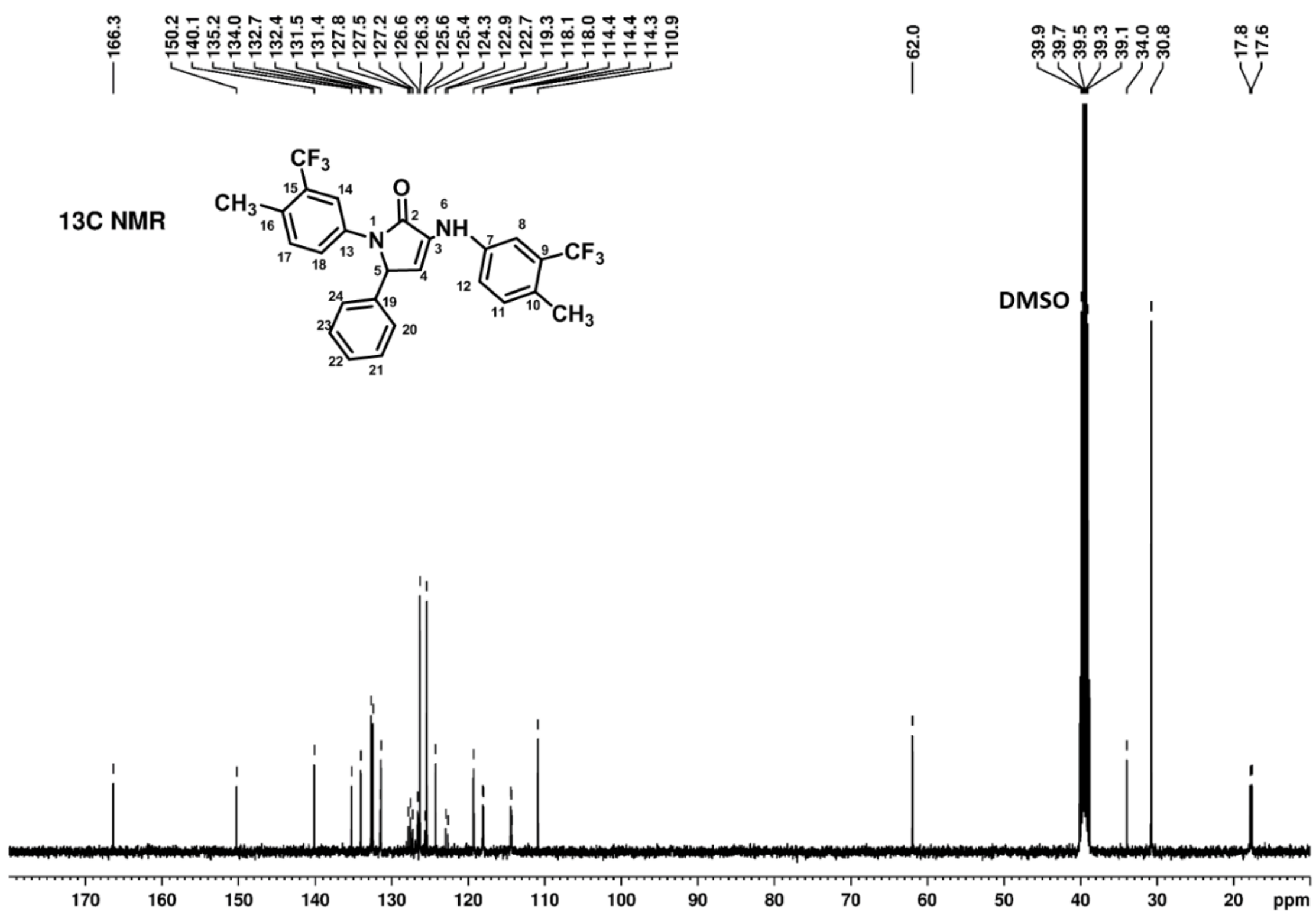

Figure 52S. ^{13}C-NMR spectrum **4m** recorded in DMSO-d_6, at 100.6 MHz.

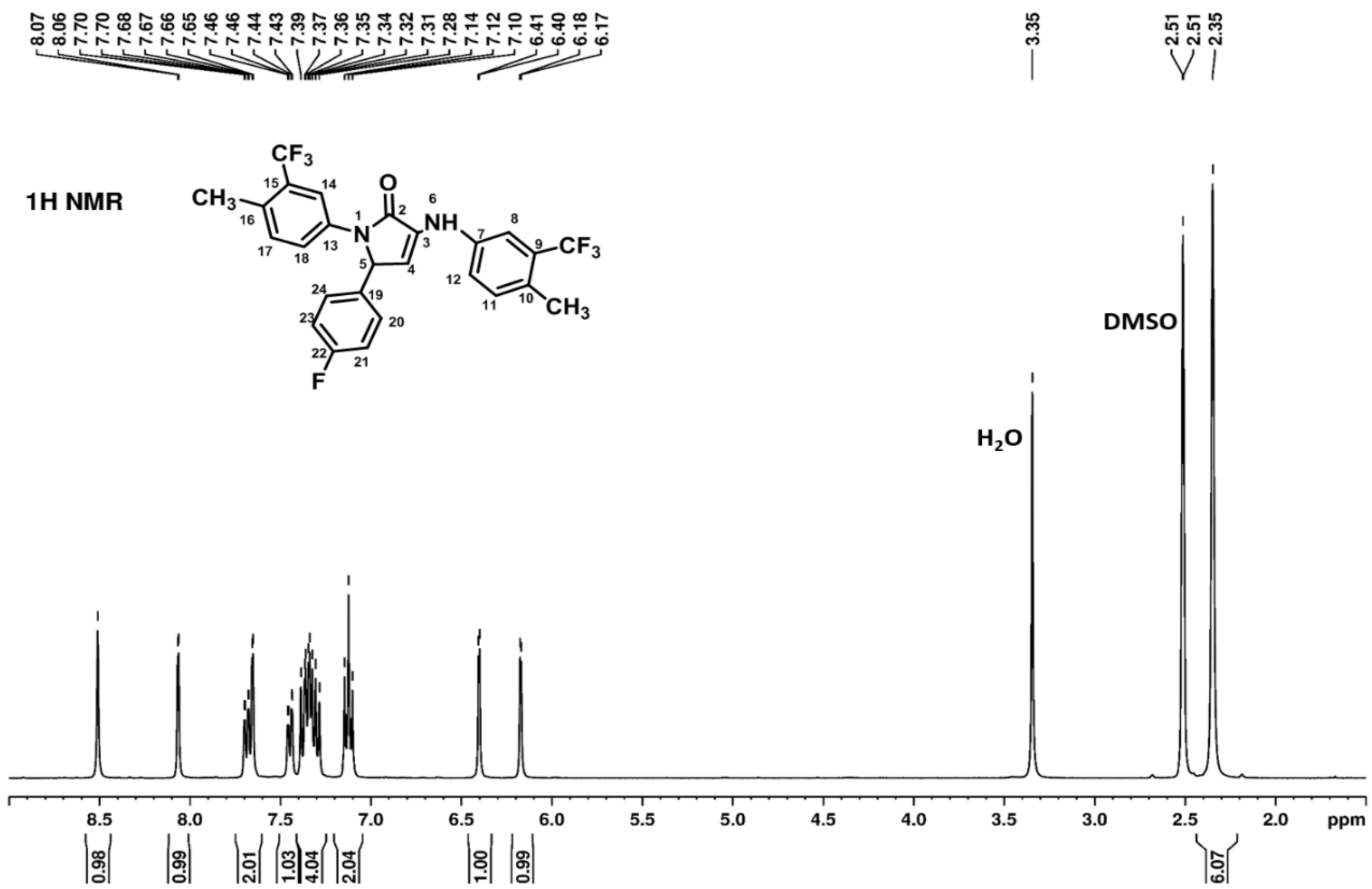

Figure 53S. ^{1}H-NMR spectrum **4n** recorded in DMSO-d6, at 400.1 MHz.

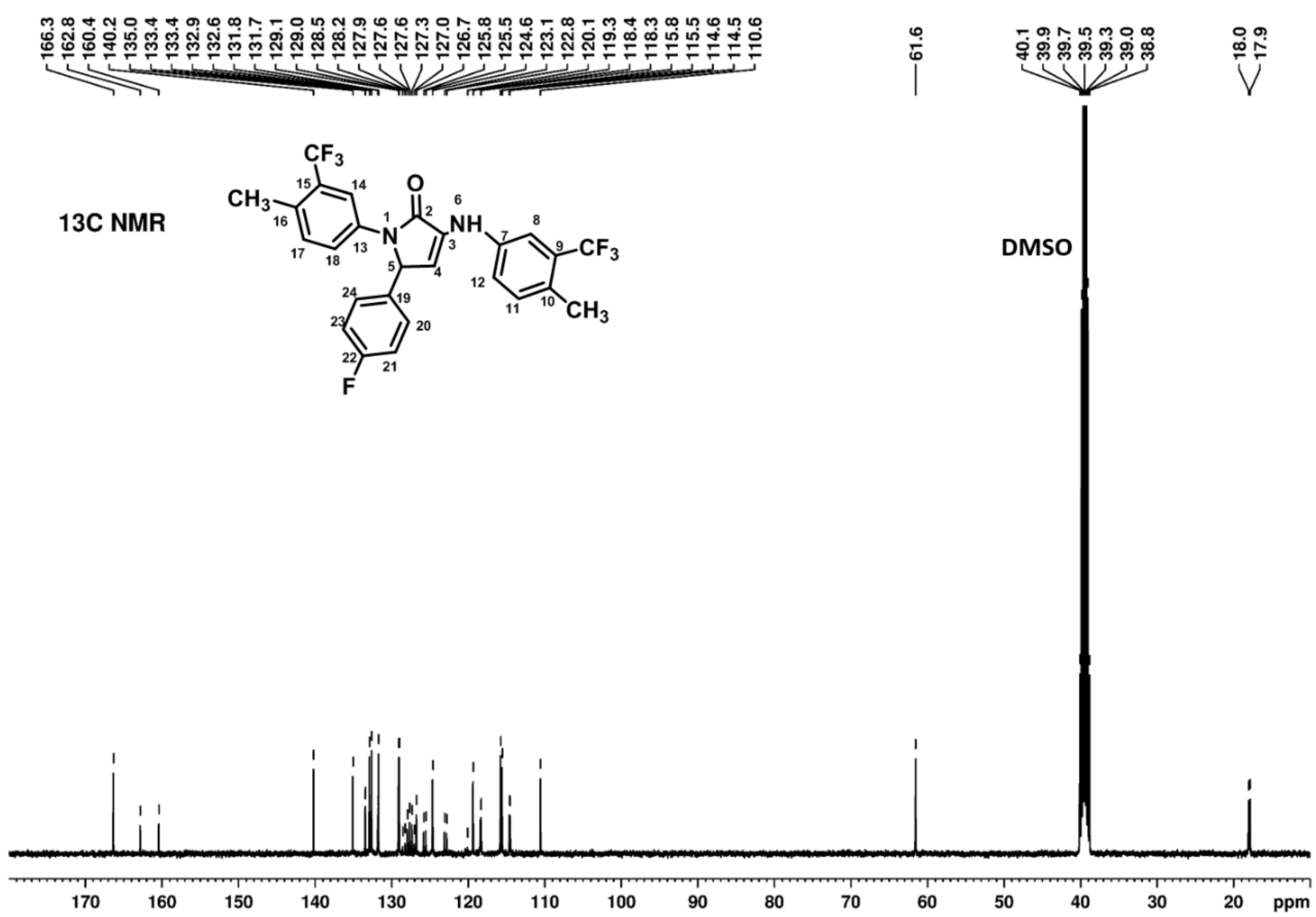

Figure 54S. ^{13}C-NMR spectrum **4n** recorded in DMSO-d$_6$, at 100.6 MHz.

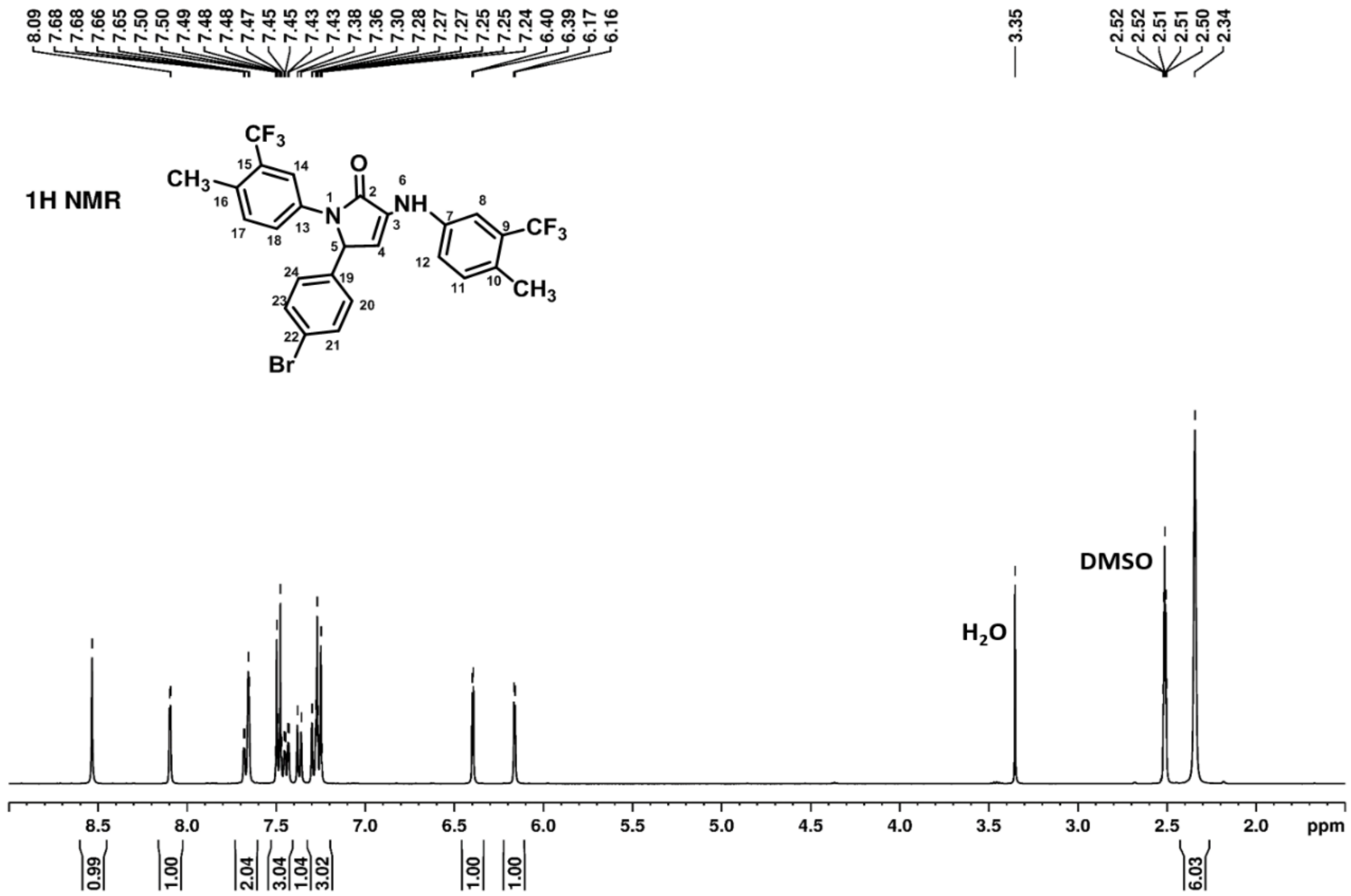

Figure 55S. ^{1}H-NMR spectrum **4p** recorded in DMSO-d6, at 400.1 MHz.

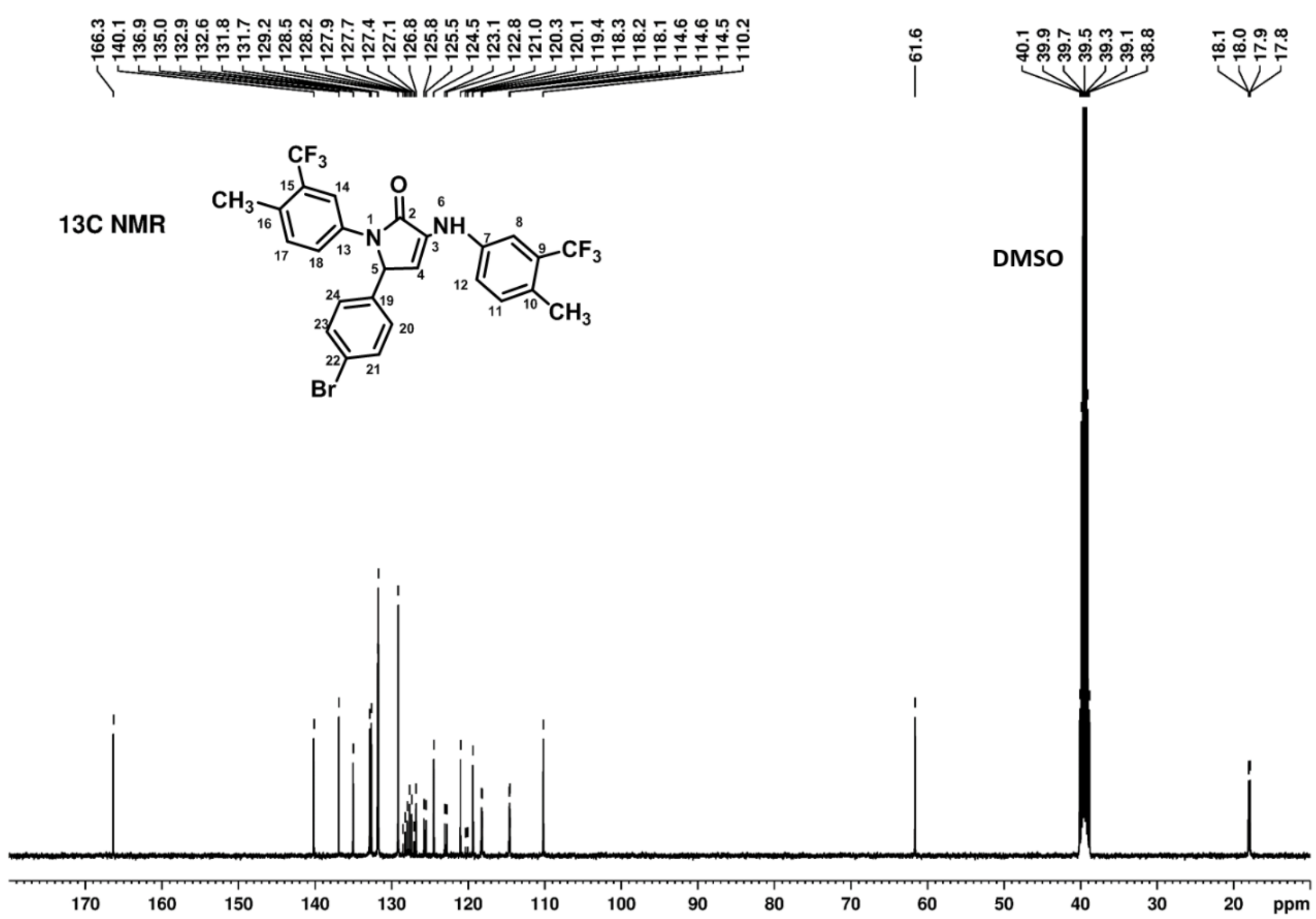

Figure 56S. ^{13}C-NMR spectrum **4p** recorded in DMSO-d_6, at 100.6 MHz.

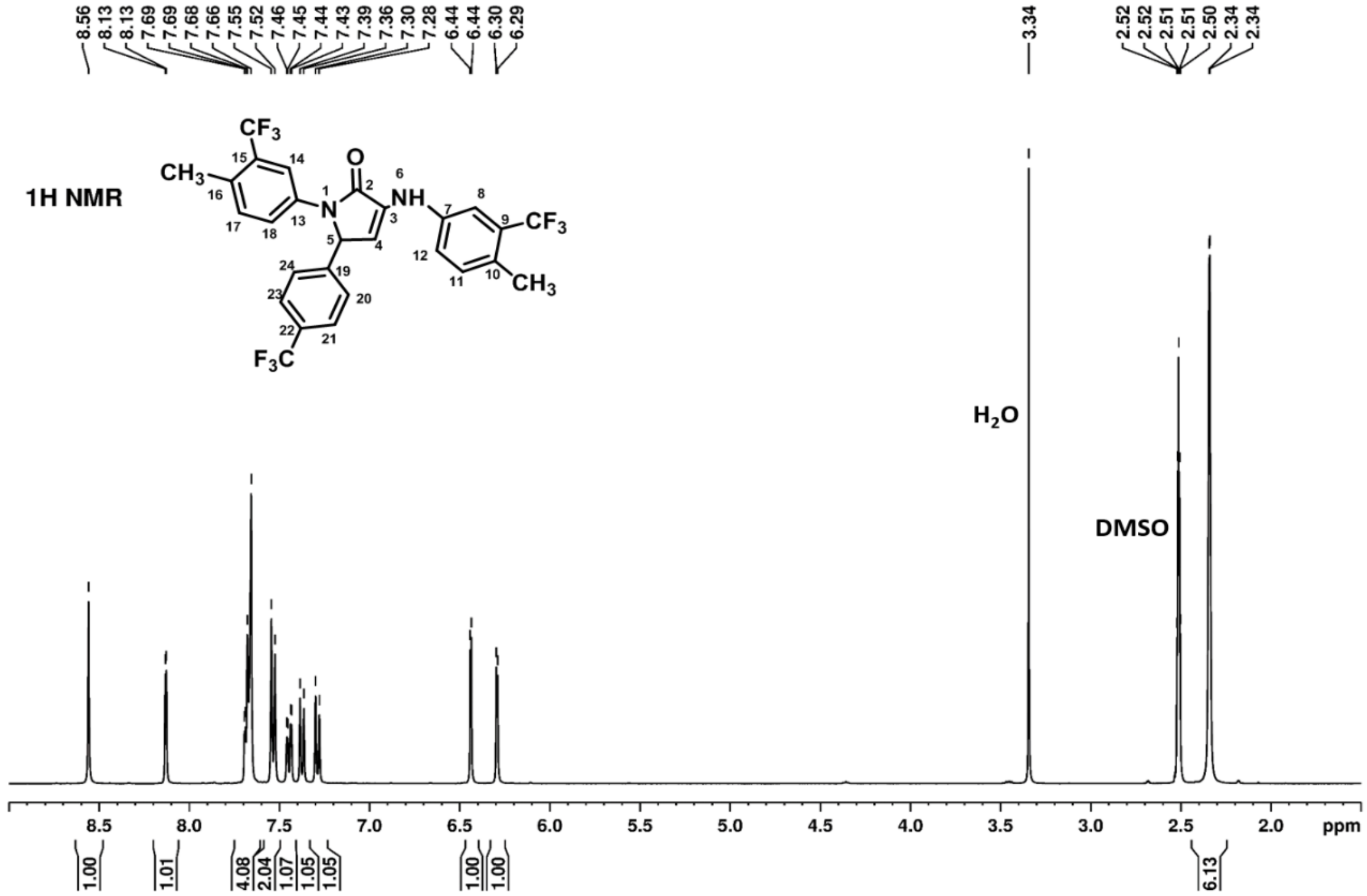

Figure 57S. ^{1}H-NMR spectrum **4r** recorded in DMSO-d6, at 400.1 MHz.

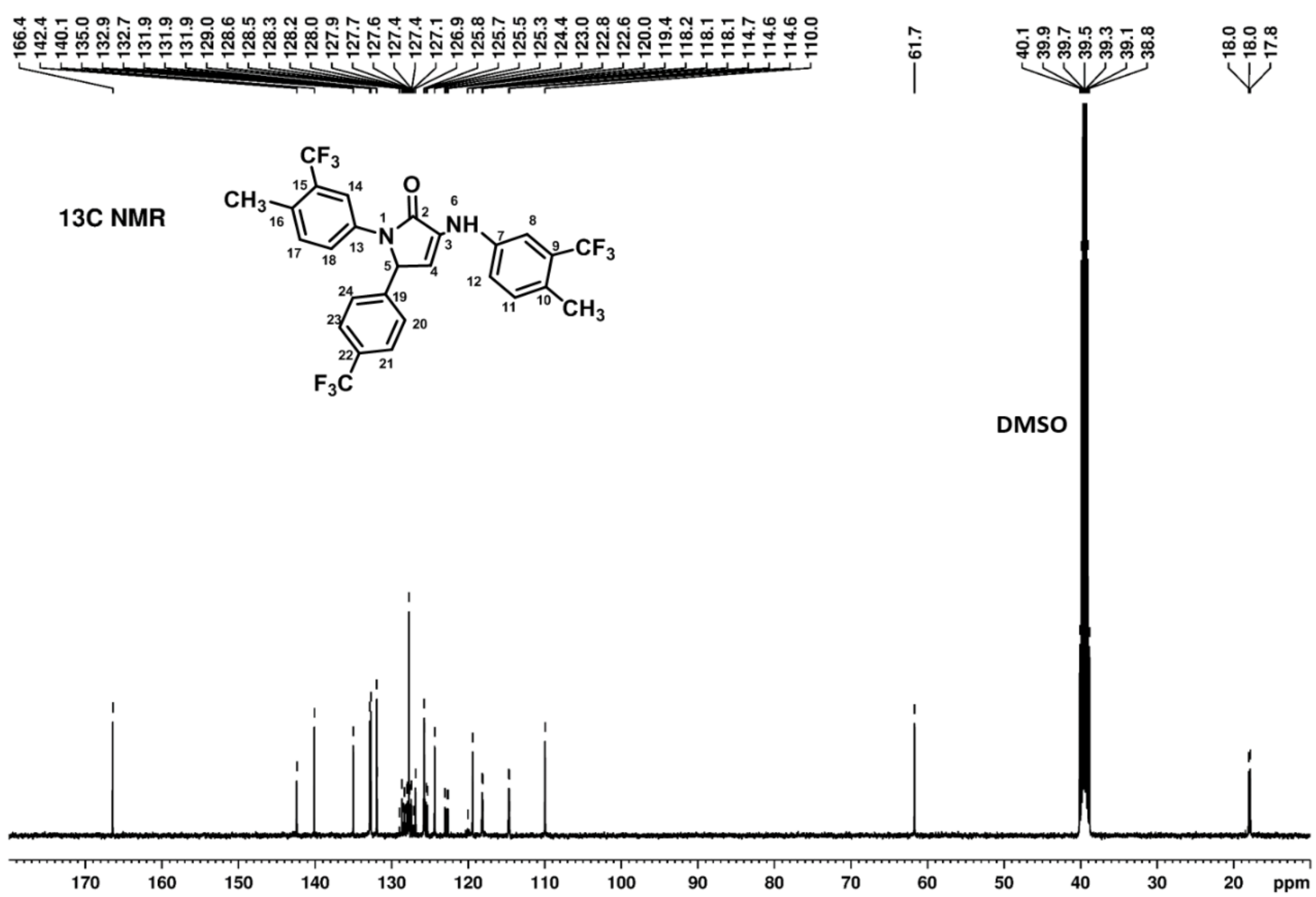

Figure 58S. ^{13}C-NMR spectrum **4r** recorded in DMSO-d_6, at 100.6 MHz.

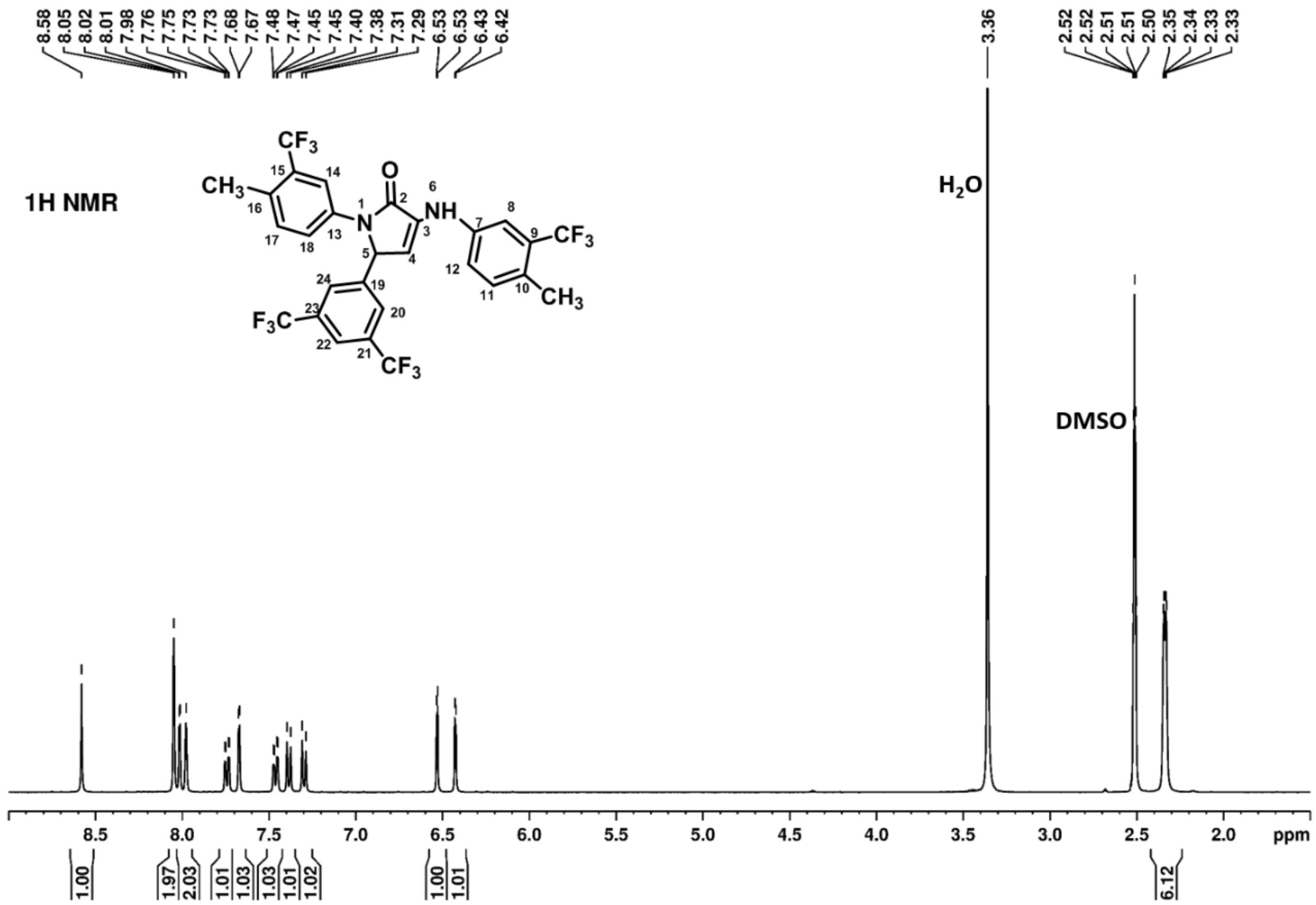

Figure 59S. ^{1}H-NMR spectrum **4s** recorded in DMSO-d6, at 400.1 MHz.

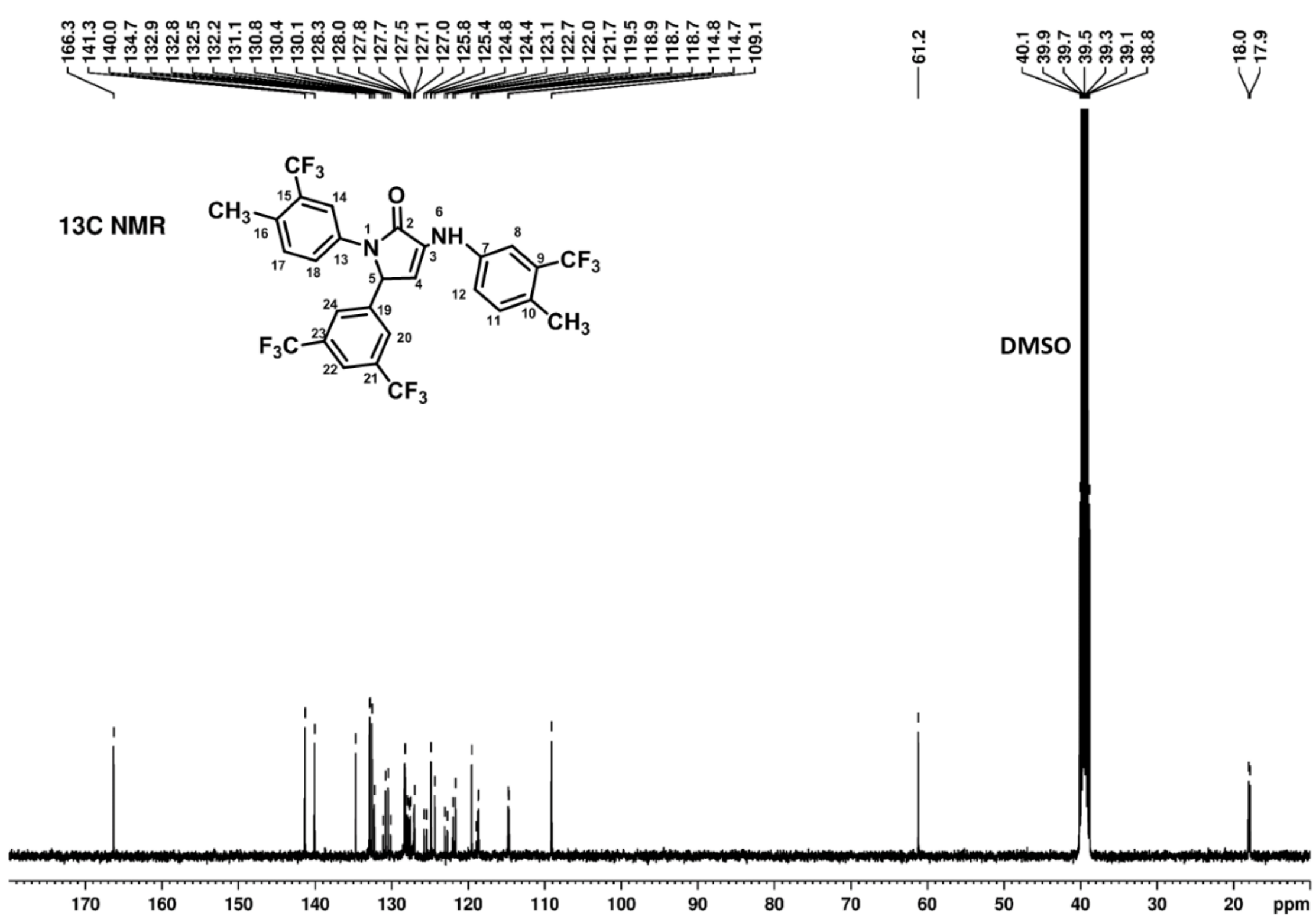

Figure 60S. ^{13}C-NMR spectrum **4s** recorded in DMSO-d_6, at 100.6 MHz.
